# Impact of Carboxyl Groups in Graphene Oxide on Chemoselective Alcohol Oxidation with Ultra-Low Carbocatalyst Loading

**DOI:** 10.1038/s41598-017-03468-4

**Published:** 2017-06-09

**Authors:** Yan Cui, Young Hee Lee, Jung Woon Yang

**Affiliations:** 1 Department of Energy Science, Sungkyunkwan University, Suwon, 16419 Republic of Korea; 2 Center for Integrated Nanostructure Physics, Institute for Basic Science (IBS), Sungkyunkwan University, Suwon, 16419 Republic of Korea

## Abstract

A highly efficient and simple chemoselective aerobic oxidation of primary alcohols to either aldehydes or carboxylic acids in the presence of nitric acid was developed, utilising 5 wt% graphene oxide as a carbocatalyst under ambient reaction conditions. Carboxylic acid functional groups on graphene oxides played a vital role in carbocatalyst activity, greatly influencing both the reactivity and selectivity. We also applied this protocol to a variant of the Knoevenagel condensation for primary alcohols and malonates with a secondary amine co-catalyst via cooperative catalysis.

## Introduction

**Figure 1:**
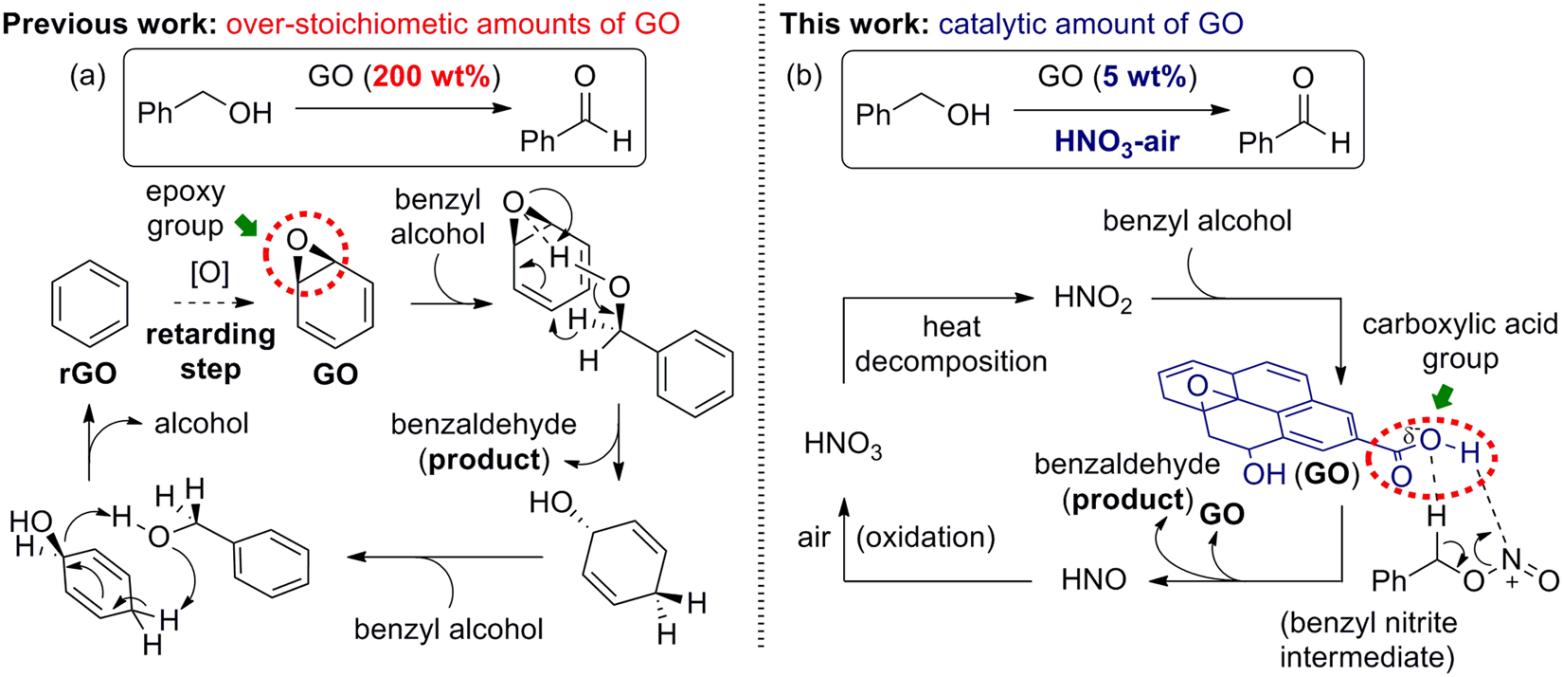
(**a**) Scarified epoxy functional group serving as a reagent. (**b**) The vital role of the carboxylic acid group in GO moieties as a catalyst for the oxidation of alcohols to aldehydes.

The development of green and sustainable chemistry utilising a transition metal-free approach has been connected with the use of non-toxic, robust catalysts with minimal or no adverse environmental or social impact. Recently, carbocatalysts, which incorporate fullerenes^1–3^, nanodiamonds^4–6^, carbon nanotubes^7–11^, and graphite/graphene^12–15^ have attracted great attention as potential sustainable catalysts. Among them, graphene oxide (GO) is emerging as a new class of heterogeneous catalyst due to its unique property of being easily manipulated with additional functional groups, such as epoxy, hydroxyl, and carboxylic acid, which are arbitrarily distributed in the carbon sheets^16, 17^. In particular, the design of the graphene functionalisation processes helps their utility in a wide range of synthetic transformations (e.g., oxidation of olefins^18^, alcohols^19, 20^, and sulfides^21^, and hydration of alkynes^22^). However, since the exposure of oxygen-containing functional groups on its surface allows GO to behave as an oxidant, it is required in over-stoichiometric quantities (200–800 wt%) in such reactions. Importantly, a detailed reaction mechanism for the oxidation of alcohol using GO or N-doped graphenes was recently proposed by the Subramanian group, based on density functional theory (DFT) calculations. It revealed that the epoxy group on GO moieties played a vital role as a hydride acceptor and base for the abstraction of protons from alcohols (Fig. 1a). Reduced graphene oxide (rGO) is formed after dehydration and regenerates to GO under aerobic conditions. The re-oxidation step was the most retarding process in this study and thus requiring over-stoichiometric amounts of GO for efficient conversions.

Recently, the Kakimoto and Peng’s research groups independently attempted to use HNO_3_ in combination with nanoshell carbon^23^ or carbon nanotubes^10^ as promoters for the selective oxidation of primary alcohols to aldehydes. Although this strategy gave a dramatic increase in both reactivity and selectivity, sub-stoichiometric quantities of carbon materials (20–50 wt%) were still required to maximise product yield and selectivity. Inspired by the use of HNO_3_ for oxidation of alcohol to aldehyde in combination with nanomaterials, we perform alcohol oxidation with GO as Brønsted acidic carbocatalyst and HNO_3_ as oxygen carrier leading to extremely high conversion and selectivity using ultra-low carbocatalyst loading (5 wt%). Excellent results are attributed to the decomposition of benzyl nitrite, which is formed by reacting benzyl alcohol and HNO_3_ in the presence of multi-purpose GO, by transferring acidic proton from GO to benzyl nitrite. In addition, aldehyde and carboxylic acid can be selectively obtained by adjustment of oxygen pressure. The most popular strategies for the chemoselective oxidation of alcohol to aldehyde or carboxylic acid rely on transition-metal catalysts (e.g., Ag, Au, and Pt)^24–31^, which can be activated by molecular oxygen to provide a satisfactory yield of the products.

**Figure 2:**
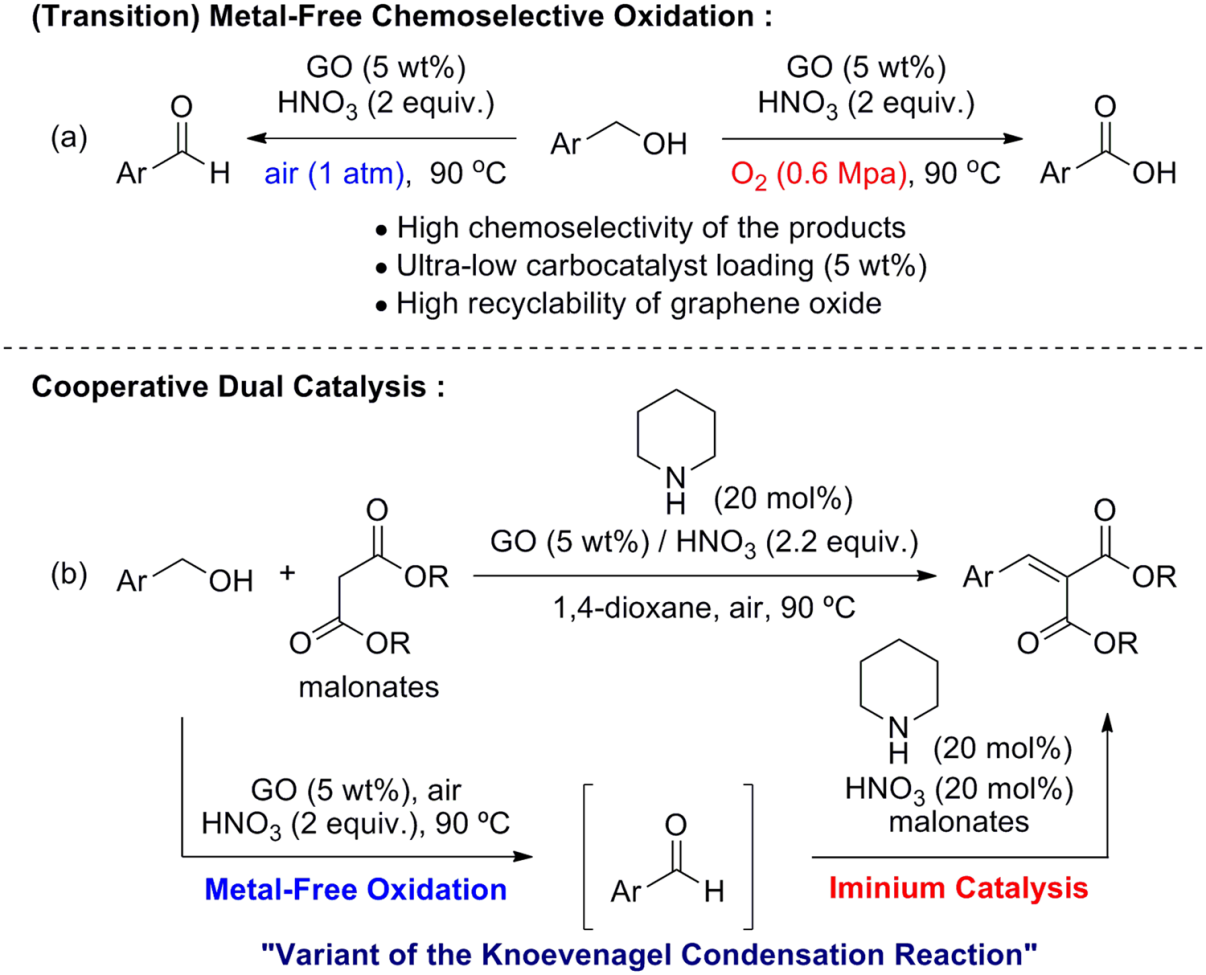
Chemoselective oxidations (**a**) and their application to a variant of the Knoevenagel condensation reaction (**b**).

Herein, we report a transition-metal-free, chemoselective aerobic oxidation of primary alcohols to either aldehydes or carboxylic acids in the presence of 5 wt% GO with HNO_3_ under ambient conditions (Figs 1b and 2a). We demonstrate that carboxylic acid functional groups on GO moieties play a vital role in the carbocatalyst, as identified by removal of the carboxylic acid group with a reducing agent (LiAlH_4_) and confirmed by FT-IR spectroscopy. Moreover, we demonstrate the utility of this method in a variant of the Knoevenagel condensation reaction^32–34^ between benzyl alcohols and malonates in combination with a secondary amine organocatalyst *via* cooperative catalysis^35–37^ (Fig. 2b).

## Results and Discussion

The investigation was commenced using benzyl alcohol **1a** as a model substrate with 67% HNO_3_ as an oxygen carrier at 90 °C under an oxygen atmosphere (Table 1). It was found that the reaction was sluggish and the desired product **2a** was obtained in 34% conversion with moderate selectivity (65%) (Table 1, entry 1). We then turned our attention to the use of GO as an activator and carbocatalyst in this reaction. In the absence of nitric acid, only a trace amount of desired product **2a** was detected after 10 h (Table 1, entry 2). Gratifyingly, a significant improvement in conversion (>99%) and selectivity (93%) was achieved when the reaction was carried out in 1,4-dioxane at 90 °C with 5 wt% of GO and 2 equiv. of 67% HNO_3_ under an oxygen atmosphere (1 atm). Meanwhile, the same reaction at 80 °C led to disappointing result. It is noteworthy that GO, HNO_3_, O_2_, and temperature appeared to all play vital roles in the oxidation. The selectivity for aldehyde decreased with a pressure increase from 1 atm to 0.5 MPa due to partial further oxidation of the aldehyde. From economical and applicability standpoints, air would constitute a more green terminal oxidant in this alcohol oxidation strategy than O_2_. Indeed, the GO-catalysed oxidation of alcohol with nitric acid took place in the presence of air, maintaining high conversion and selectivity compared with O_2_. Surprisingly, a higher catalyst loading (20 wt% GO) had a negative effect on both the aldehyde yield and dispersion behaviour of GO. We then examined solvent effects; however, other solvents, such as DMF, THF, and toluene, failed to achieve better performances than 1,4-dioxane.

**Table 1:** Optimisation of reaction conditions for graphene oxide-catalysed oxidation of benzyl alcohol to benzaldehyde using HNO_3_ under an oxygen/or air atmosphere^a^.


Entry	GO (mg)	HNO_3_ (equiv.)	Oxidant	Solvent	Temp (°C)	Time (h)	Conv. (%)^b^	Sel. (%)^b^
1	—	2	O_2_ (1 atm)	1,4-dioxane	90	5	34	65
2	5	—	O_2_ (1 atm)	1,4-dioxane	90	10	trace	—
3	5	2	O_2_ (1 atm)	1,4-dioxane	90	3	>99	93
4	5	2	O_2_ (1 atm)	1,4-dioxane	80	24	62	93
5	5	2	O_2_ (0.5 MPa)	1,4-dioxane	90	5	76	67
6	—	2	air	1,4-dioxane	90	5	33	65
7	5	—	air	1,4-dioxane	90	10	trace	—
**8**	**5**	**2**	**air**	**1,4-dioxane**	**90**	**3**	**>99**	**94**
9	20	2	air	1,4-dioxane	90	3	>99	84
10	5	2	air	DMF	90	3	65	56
11	5	2	air	THF	90	3	85	56
12	5	2	air	toluene	90	3	92	67
13	5	1	air	1,4-dioxane	90	3	64	98
14	5	3	air	1,4-dioxane	90	3	>99	27

^a^Reaction conditions: **1a** (1 mmol), graphene oxide (5–20 wt%), 67% nitric acid (2 mmol), solvent (2 mL), O_2_ (0.5 Mpa–1 atm) or air (1 atm), 80–90 °C. ^b^Determined by gas chromatography using an internal standard (biphenyl).

Finally, it was observed that the equivalents of nitric acid was also a crucial parameter for the reaction, and the desired product obtained in 64% conversion with 98% selectivity, and >99% conversion with 27% selectivity, when using 1 equiv. of 67% HNO_3_ and 3 equiv. of 67% HNO_3_, respectively. Thus, GO (5 wt%), 67% HNO_3_ (2 equiv.), and air (1 atm) in 1,4-dioxane at 90 °C were verified as the optimised reaction condition for the oxidation of alcohol to aldehyde (Table 1, entry 8).

**Figure 3:**
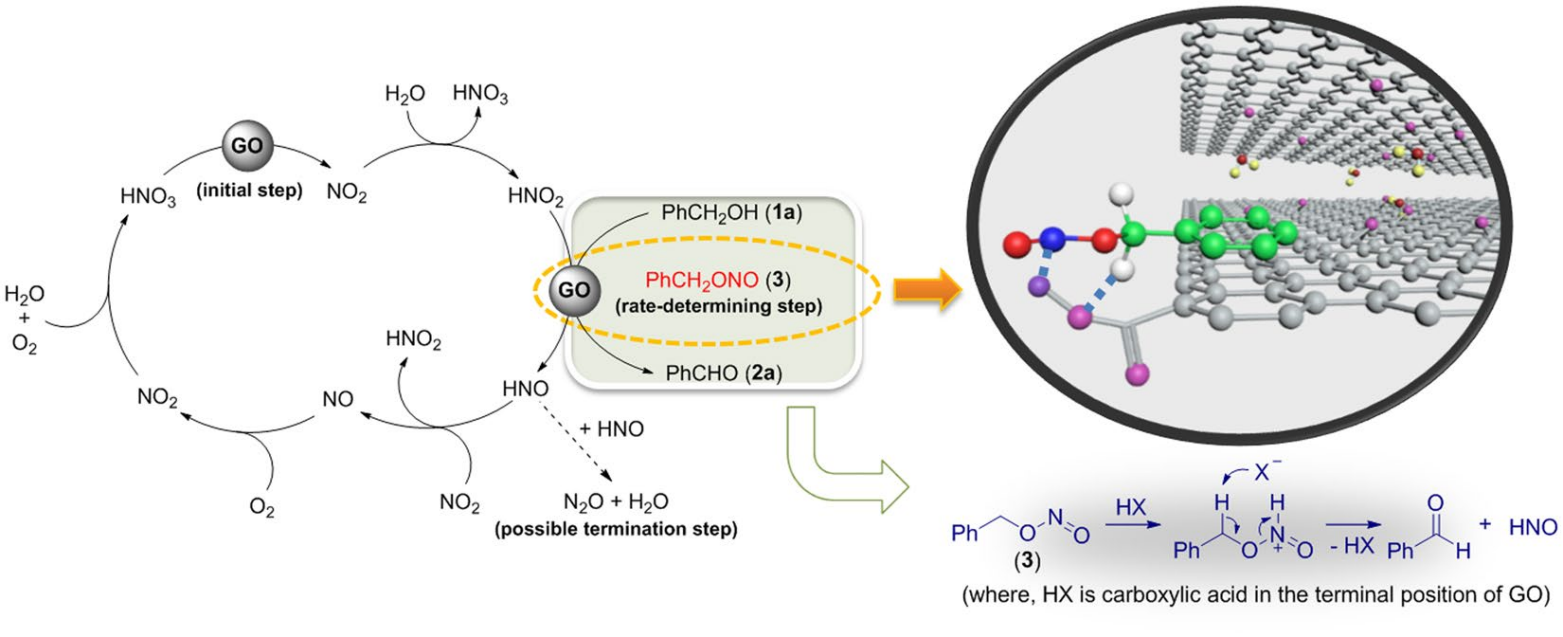
Proposed mechanism for the oxidation of primary alcohol to aldehyde.

We have proposed a reaction mechanism for the oxidation of primary alcohol to aldehyde using a GO-HNO_3_-O_2_ system in Fig. 3, as follows: (i) thermal decomposition of HNO_3_ to NO_2_ could be accelerated by GO, which is strongly related to its large specific surface area and thermal conductivity. The decomposed NO_2_ gas can easily be adsorbed by GO because of vacancies or small holes on the GO surface^38^ (see Supplementary Information). GO defects are usually considered as heterogeneous active sites for gas adsorption. The resulting NO_2_ quickly reacts with H_2_O to produce a mixture of HNO_3_ and HNO_2_; (ii) benzyl nitrite **3** is formed by reacting benzyl alcohol **1a** with HNO_2_ in the presence of a catalytic amount of GO, which is the rate-determining step of the overall reaction^39, 40^. Cleavage of N–O bond of benzyl nitrite could be accelerated by protonation of the nitrogen atom in the benzyl nitrite moiety and subsequent abstraction of the proton by a strong base at the benzylic position of benzyl nitrite, leading to the desired aldehyde product **2a** and HNO. In addition, the confined structural environments, made up of π-π interactions between mono/ few layered graphene sheets of GO and aromatics of the benzyl nitrite moiety, help to promote the cleavage reaction^10^; (iii) HNO is oxidised by NO_2_ to give a mixture of HNO_2_ and NO, wherein NO_2_ acts as an oxidising agent. Meanwhile, a small portion of HNO undergoes dimerisation to form nitrous oxide (N_2_O), terminating the HNO_3_ catalytic cycle. (iv) liberated NO is also re-oxidised to NO_2_ by O_2_, and then reacted with H_2_O to generate HNO_2_ and HNO_3_ species. Further oxidation residual HNO_2_ from the previous step to HNO_3_ could occur with the involvement of O_2_ and GO catalyst. Consequently, it transpired that four components played a vital role in this process; (i) O_2_, acting as the terminal oxidant; (ii) HNO_3_, acting as an initiator for oxidation; (iii) GO, acting as a multi-purpose activator for gas adsorption and regeneration of HNO_3_ with O_2_; (iv) *Pending carboxylic acid groups onto GO are one of the crucial roles in the cleavage of N-O single bond of benzyl nitrite, it makes the catalytic cycle more efficient*.

**Figure 4:**
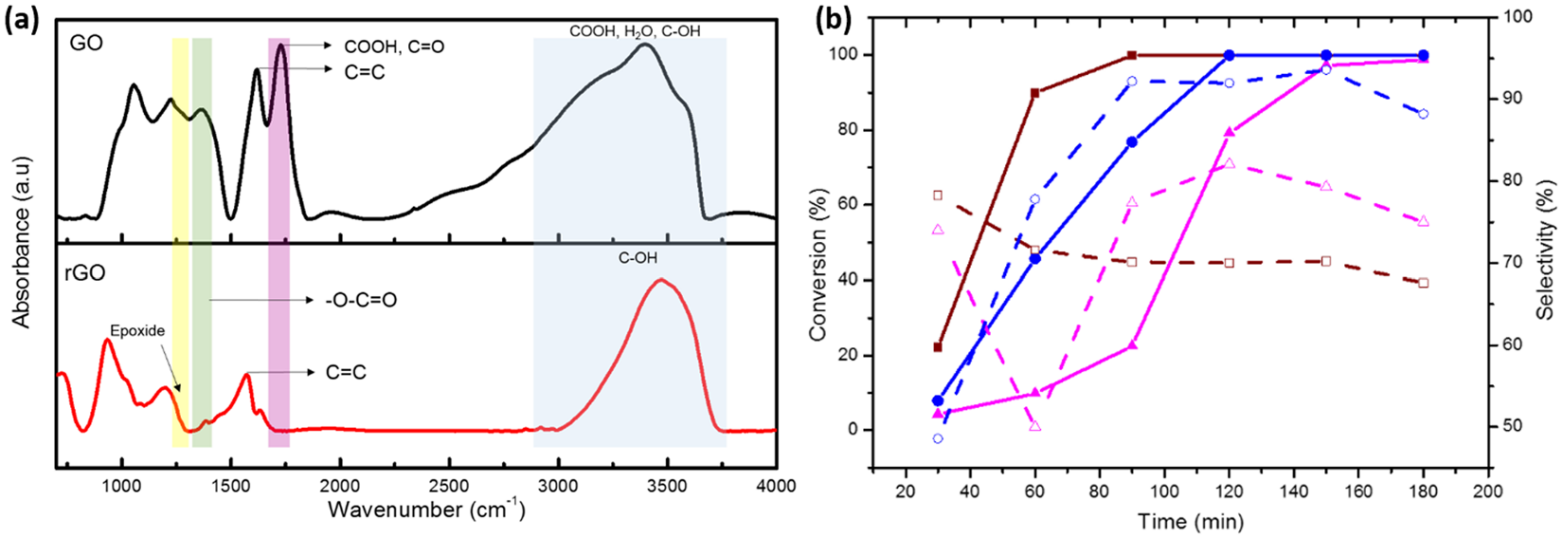
(**a**) FT-IR spectra of rGO and GO. (**b**) Solid line: conversion of alcohol; dotted line: selectivity for aldehyde (brown: rGO catalyst; blue: GO catalyst; pink: no carbocatalyst).

To confirm the vital role of carboxylic acid on GO, rGO was synthesised according to a literature procedure and characterised by FT-IR spectroscopy, as presented in Fig. 4. This revealed that the intensity of C=O stretching in the –CO_2_H group (1650–1740 cm^−1^)^41^ and the vibration band of epoxy group (1280–1330 cm^−1^) on rGO were significantly lower than those of on GO, which was attributed to reduction of carboxylic acid functional groups by LiAlH_4_, as strong reducing agent. Subsequently, the oxidation of benzyl alcohol to benzaldehyde was carried out separately in the presence of rGO and GO, and without carbocatalyst. rGO exhibited superior catalytic activity in the conversion of benzyl alcohol to benzaldehyde compared with GO and without carbocatalyst, which was presumably ascribed to the large specific surface area and high thermal conductivity. Specifically, a larger surface area and higher thermal conductivity in rGO, can be expected to produce faster decomposition of HNO_3_ to HNO_2_. Benzyl alcohol **1a** was quickly consumed within 90 min by reacting with the HNO_2_ generated in the presence of rGO, resulting in the complete formation of benzyl nitrite **6**. On the other hand, GO displayed the second-highest catalytic activity whereas the reaction without carbocatalyst showed the poorest activity in terms of substrate conversion. The conversion rates increased in the following order: rGO ≫ GO ≫ no carbocatalyst

*Conversion was related to the decomposition rate of HNO*_*3*_ *, whereas chemoselectivity was probably associated with the decomposition rate of benzyl nitrite*. It was postulated that the decomposition rate of benzyl nitrite would be highly dependent on –CO_2_H contents in the GO catalyst.

To verify this assumption, we attempted to use either GO or rGO in this study. Indeed, the use of GO bearing a high carboxylic acid content led to a gradual increase in the selectivity for product **2a**, in parallel with increasing conversion of **1a**, whereas the use of rGO with trace amounts of carboxylic acid content led to a decrease in product **2a** selectivity inversely proportional to substrate **1a** conversion (Fig. 4b). As shown in Fig. 3, these results clearly supported that carboxylic acid groups on GO remarkably accelerated the cleavage of N-O single bond in benzyl nitrite, increasing the turnover frequency (TOF) of the GO catalyst and leading to higher chemoselectivity. Without carbocatalyst, a lower chemoselectivity for product **2a** was observed within 60 min due to the slower decomposition rate of benzyl nitrite **3** and the lack of strong base to abstract the α-acidic proton from benzyl nitrite. However, once HNO was formed, product selectivity dramatically increased by accelerating deprotonation of the α-acidic proton in benzyl nitrite with conjugated base NO^−^ (pKa = 11.4).

**Figure 5:**
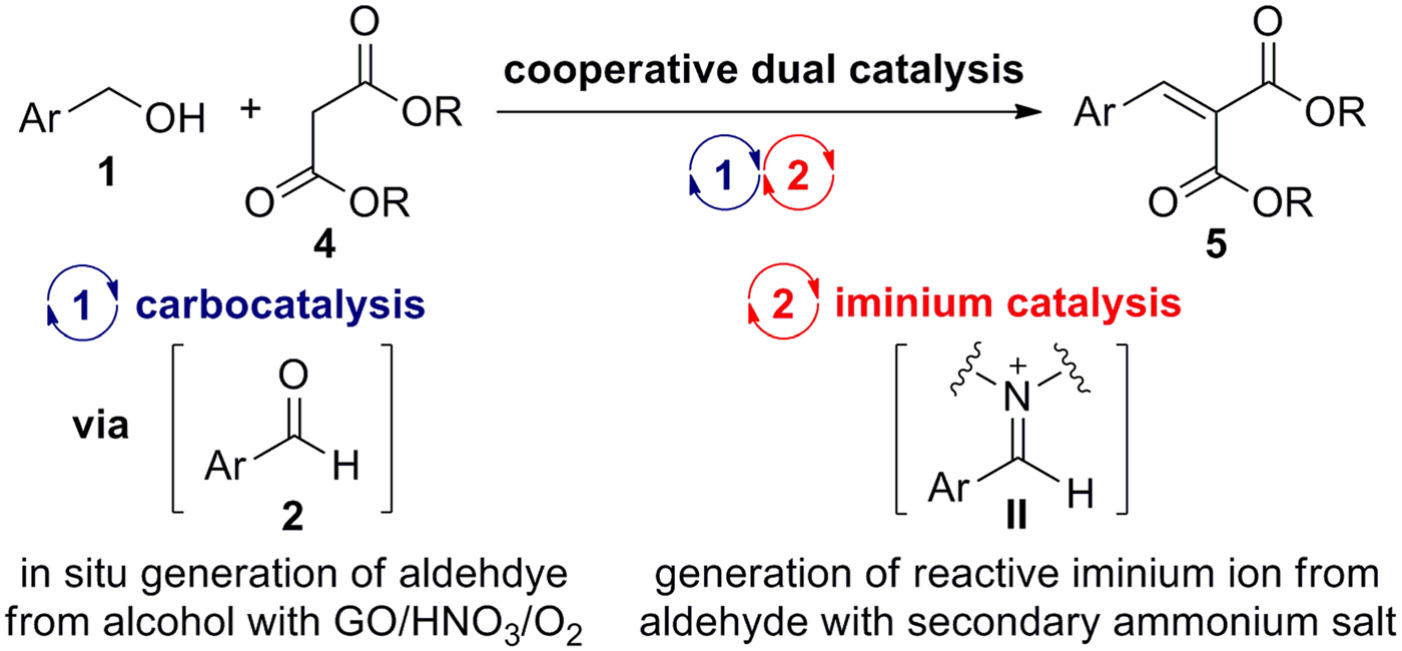
Strategy for a variant of the Knoevenagel condensation *via* cooperative catalysis.

Given recent advances in cooperative catalysis, we performed a preliminary investigation into the transition-metal-free variation of the Knoevenagel condensation using a cooperative dual catalytic system (Fig. 5).

The current system consisted of the following steps: (i) GO-catalysed direct oxidation of primary alcohol **1** to aldehyde **2** *via* carbocatalysis; (ii) activation of the resulting aldehyde by secondary ammonium salt **I** to produce iminium ion **II**, followed by subsequent nucleophilic attack by malonate **4** to give intermediate **III**; (iii) removal of an α-proton from the 1,3-dicarbonyl group by base furnishes the corresponding Knoevenagel adduct **5** and regenerates organocatalyst **I** (Fig. 5). *The Knoevenagel reaction of the primary alcohol with malonate does not occur in the absence of GO and the secondary amine*.

The selection of reciprocally compatible and cooperative catalysts (e.g., GO and secondary amine as a Lewis base) may be crucial to the success of this domino reaction^42, 43^. For this reason, we focused our studies on a series of secondary amines as organocatalyst in combination with GO (Table 2). The model reaction of benzyl alcohol **1a** with diethyl malonate **4a** was performed in the presence of both 20 mol% secondary amine catalyst and 5 wt% GO in 1,4-dioxane at 90 °C under oxygen atmosphere (1 atm). Among various types of organocatalysts, dibenzyl amine and morpholine exhibited no catalytic activity, while piperidine showed slightly better performance in terms of activity and yield (18%). After various trials, solvent selection was found to benefit the reaction. To our delight, the desired Knoevenagel product **5a** was obtained in 66% yield when DMF was used as solvent. However, no further improvement of chemical yield was achieved although the reaction time was prolonged to 24 h. We realised that individual reactions, such as oxidation and the Knoevenagel reaction, were highly solvent dependent. For instance, the metal-free oxidation of alcohol utilising GO/HNO_3_/O_2_ proceeded smoothly in 1,4-dioxane to give the corresponding aldehyde in excellent yield (>90% in most cases), whereas the second sequencing reaction (Knoevenagel reaction) using a secondary ammonium salt as organocatalyst was favoured in DMF solvent, which was associated with the dissociation of ammonium salt to enhance its reactivity dramatically. Therefore, it was necessary to find a suitable compromise in solvent ratios of 1,4-dioxane and DMF in order to improve the reaction rate and chemical yield. Gratifyingly, a higher yield (72% yield) of **5a** was achieved when the reaction was conducted in 1,4-dioxane/DMF (v/v = 1:3).

**Table 2:** Optimisation of a variant of the Knoevenagel condensation^a^.


Entry	Amine catalyst	Solvent	Time (h)	Yield (%)^b^
1	dibenzyl amine	1,4-dioxane	24	—
2	morpholine	1,4-dioxane	24	—
3	piperidine	1,4-dioxane	24	18
4	piperidine	CH_3_CN	24	17
5	piperidine	DMSO	24	20
6	piperidine	DMF	24	66
7	piperidine	1,4-dioxane:DMF (1:1, v/v)	24	33
**8**	**piperidine**	**1,4-dioxane:DMF (1:3, v/v)**	**24**	**72**
9	piperidine	1,4-dioxane:DMF (1:5, v/v)	24	53

^a^Reaction conditions: **1a** (1 mmol), **4a** (1 mmol), graphene oxide (5 wt%), 67% nitric acid (2.2 mmol), secondary amine catalyst (20 mol%), 4 Å molecular sieves, solvent (2 mL, v/v = 1:3), O_2_ (1 atm), 90 °C. ^b^Isolated product yield.

**Figure 6:**
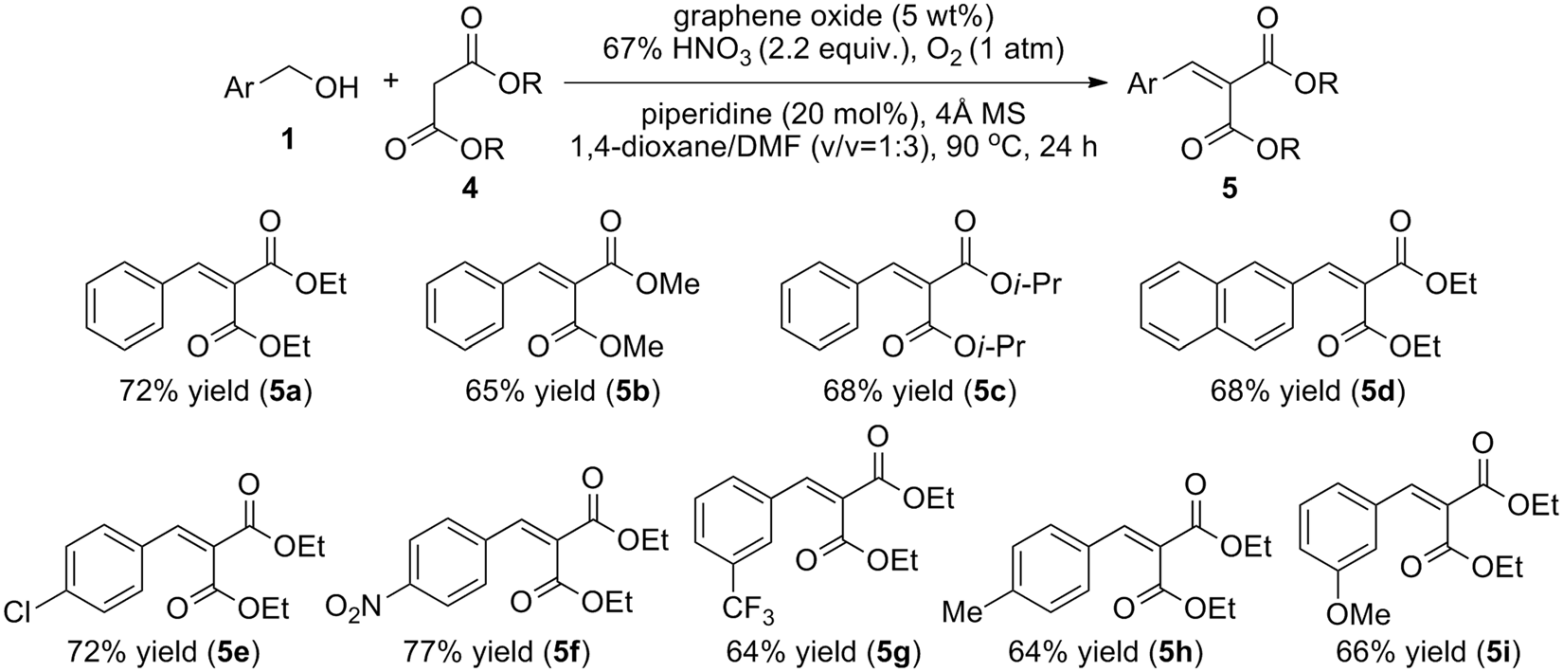
Reaction scope for a variant of the Knoevenagel condensation *via* cooperative catalysis.

With optimised reaction conditions in hand, we then investigated the reaction scope by varying the nature of substituents on the aryl ring and introducing malonate derivatives with varying ester substituents (Fig. 6).

It transpired that both the alkyl residues (e.g., methyl, ethyl, and iso-propyl) in the malonic acid esters, and various substituents (e.g., electron-withdrawing and electron-donating groups) on the aromatics influenced on the chemical yield. In general, dialkyl benzylidene malonate and its derivatives obtained in good yields (64–77%) through sequential GO-catalysed oxidation and piperidine-catalysed Knoevenagel condensation.

Encouraged by the possibility of further oxidation of the aldehyde under high pressure of O_2_ (Table 1, entry 5), we quickly performed a general optimisation of the direct oxidation of primary alcohols to carboxylic acids by varying the pressure (Table 3).

**Table 3:** Optimization of the reaction conditions for the graphene oxide-catalyzed oxidation of benzyl alcohol to carboxylic acid with HNO_3_ under variation of oxygen pressure^a^.


Entry	O_2_ [pressure (Mpa)]	Yield (%)^b^	Ratio of **2a**:**6a** ^c^
1	0.2	62	78:22
2	0.4	76	56:44
3	0.5	76	32:68
**4**	**0.6**	**>99**	**10:90**

^a^Reaction conditions: **1a** (1 mmol), graphene oxide (5 wt%), 67% nitric acid (2 mmol), 1,4-dioxane (2 mL), O_2_ (0.2–0.6 Mpa), 90 °C. ^b^Isolated overall yield of **2a** and **6a**. ^c^Determined by gas chromatography using an internal standard (biphenyl).

This study revealed that pressure strongly affected the ratio of aldehyde and carboxylic acid. At 0.6 MPa of O_2_, the highest yield and proportion of **6a** was observed (Table 3, entry 4).

Having established two successful and complementary chemoselective oxidations, the exploratory results are summarised in Table 4. In the course of either aldehyde formation or carboxylic acid formation, most of the aldehyde products (**2a**–**2f**) were afforded in excellent yields (83–93%) regardless of the substituents on the aryl group, whereas carboxylic acid products (**6a**–**6f**) were obtained in excellent yields (83–91%) except for bromo- and nitro-substituted benzoic acid derivatives.

**Table 4:** Scope of chemoselective oxidation reactions^a^.


Aldehyde Formation				Carboxylic Acid Formation			
Entry	Product	Time (h)	Yield (%)^b^	Entry	Product	Time (h)	Yield (%)^b^
1		3	93	7		5	90
2		2.5	93	8		24	83
3		2	83	9		24	77
4		2	89	10		24	73
5		2.5	93	11		5	91
6		2.5	91	12		5	90

^a^Reaction conditions for aldehyde formation: **1** (1 mmol), graphene oxide (5 wt%), 67% nitric acid (2 mmol), 1,4-dioxane (2 mL), air (1 atm), 90 °C; Reaction conditions for carboxylic acid formation: **1** (1 mmol), graphene oxide (5 wt%), 67% nitric acid (2 mmol), 1,4-dioxane (2 mL), O_2_ (0.6 MPa), 90 °C. ^b^Isolated product yield.

**Figure 7:**
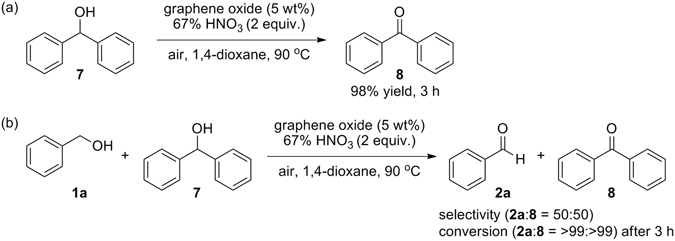
Aerobic oxidation of secondary alcohol (**a**) and chemoselective oxidation of primary alcohol in the presence of secondary alcohol (**b**).

We also investigated aerobic oxidation of secondary alcohol and chemoselective oxidation of primary alcohol in the presence of secondary alcohol under optimal conditions (Fig. 7). Benzhydrol **7** as secondary alcohol substrate smoothly oxidised to benzophenone **8** in almost perfect yield (98%) after 3 h (Fig. 7a). Different type of primary and secondary alcohol were subjected to the oxidation reaction under optimal conditions. 1:1 Mixture of the corresponding products (**2a** and **8**) were formed, indicating that no chemoselectivity was observed. In case of primary benzyl alcohol **1a**, however, the corresponding aldehyde **2a** was chemoselectively formed without over-oxidation to carboxylic acid **6a** (Fig. 7b).

Finally, we examined the recyclability of GO in the oxidation of benzyl alcohol to benzaldehyde. The results showed that the recovered catalyst exhibited high catalytic activity (>99% conversion) and could be reused 20 times for the chemoselective formation of aldehyde (see Supplementary Information).

## Summary

We have demonstrated that the chemoselective synthesis of aldehydes and carboxylic acids from the same alcoholic substrate catalysed by unmodified graphene oxide as carbocatalyst. Attractive features of our approach include (i) elucidating the function of carboxylic acid group on graphene oxide in carbocatalysis by measuring product chemoselectivity with different carboxylic acid contents; (ii) demonstrating their application to cooperative catalysis of a variant of the Knoevenagel condensation between benzyl alcohol and malonate; and (iii) showing the recyclability of the GO carbocatalyst without a significant decrease in product chemoselectivity. A further exploration of the GO-HNO_3_-O_2_ system is currently in progress in our laboratories.

## Methods

### General procedure for oxidation of alcohol to aldehyde

A 10-mL two necked, round-bottomed flask equipped with a magnetic stirring bar, a reflux condenser, and a glass stopper was charged with the corresponding primary alcohol (1 mmol) and graphene oxide (5 wt%) in 1,4-dioxane (2 mL). After sonication for 1 min, 67% nitric acid (2 mmol) was added dropwise into the reaction mixture, and the resulting mixture allowed to stir at 90 °C for 2–3 h under open-air conditions. After reaction completion, the resulting mixture was diluted with dichloromethane and filtered through a 0.45 µm syringe filter. The resulting solution was rinsed with deionised water and brine, dried over Na_2_SO_4_, and concentrated under reduced pressure. The residue was purified by column chromatography on silica gel with diethyl ether and pentane as the eluting solvents to afford the corresponding aldehyde.

### General procedure for oxidation of alcohol to carboxylic acid

Primary alcohol (1 mmol), graphene oxide (5 wt%), and 1,4-dioxane (2 mL) were added to a glass vessel. After sonication for 1 min, 67% nitric acid (2 mmol) was added dropwise into the reaction mixture, and then the glass vessel was placed into an autoclave. The autoclave was flushed three times with O_2_ (0.2 MPa), then pressurized to 0.6 MPa. The autoclave was placed into a preheated oil bath at 90 °C for the desired reaction time, then cooled to room temperature, and carefully depressurised. The reaction mixture was diluted with ethyl acetate and filtered through a 0.45 µm syringe filter. The resulting solution was rinsed with deionised water and brine, dried over Na_2_SO_4_, and concentrated under reduced pressure. The residue was purified by column chromatography on silica gel with ethyl acetate as the eluting solvent to afford the corresponding carboxylic acid.

### General procedure for a variant of the Knoevenagel condensation between alcohol and malonate

A 10-mL two necked, round-bottomed flask equipped with a magnetic stirring bar, a reflux condenser, and a glass stopper was added oven-dried 4 Å molecular sieves. The flask was then flame-dried under vacuum and subsequently filled with argon. The flask was charged with piperidine (20 mol%), 67% nitric acid (2.2 mmol), and 1,4-dioxane/DMF (v/v = 1:3) and stirred for 10 min. Primary alcohol (1 mmol), malonate (1 mmol), graphene oxide (5 wt%), and 1,4-dioxane (2 mL) were then added to the reaction mixture. After sonication for 1 min, the resulting mixture was stirred at 90 °C for 24 h under 1 atm of O_2_. Upon completion of the reaction, the mixture was cooled to room temperature, filtered through 0.45 µm membrane filter paper, and washed with deionised water and ethyl acetate. The organic layers were combined, dried over Na_2_SO_4_, and concentrated under reduced pressure. The residue was purified by flash column chromatography on silica gel with ethyl acetate and hexanes as the eluting solvents to give the corresponding Knoevenagel condensation product.

## Electronic supplementary material

Supplementary Information
