# Supplementary material for: Impact of Carboxyl Groups in Graphene Oxide on Chemoselective Alcohol Oxidation with Ultra-Low Carbocatalyst Loading
Source: Sci Rep. 2017 Jun 9;7:3146. doi: 10.1038/s41598-017-03468-4 (PMC5466679; doi:10.1038/s41598-017-03468-4)

Supplementary Information

Impact of Carboxyl Groups in Graphene Oxide on Chemoselective Alcohol Oxidation with Ultra-Low Carbocatalyst Loading

Yan Cui[1], Young Hee Lee[1,2*], and Jung Woon Yang[1*]

1. Department of Energy Science, Sungkyunkwan University, Suwon 440-746, Republic of Korea
2. Center for Integrated Nanostructure Physics, Institute for Basic Science (IBS), Sungkyunkwan University, Suwon 440-746, Republic of Korea

E-mail: leeyoung@skku.edu (Y. H. Lee), Tel: +82-31-299-6507; jwyang@skku.edu (J. W. Yang), Tel: +82-31-299-4276

Contents

General Remarks and Experimental Procedures S2
Characterization Data for Products S4
References S10
^{1}H NMR and ^{13}C NMR Spectra of Products S11
Analysis of Graphene Oxide (GO) and Reduced Graphene Oxide (rGO)
& Test for NO_2 Absorption using GO S34
Recyclability of the GO Carbocatalyst S35

General Remarks

Thin-layer chromatography (TLC) was performed on Merck silica gel 60 F254. ^{1}H NMR spectra were recorded on a Varian at 500 MHz in $CDCl_3$ (δ 7.26 ppm) or DMSO-d_6 (δ 2.50 ppm), ^{13}C NMR spectral measurements were performed at 125 MHz using $CDCl_3$ (δ 77.16 ppm) or DMSO-d_6 (δ 39.52 ppm). ^{19}F NMR spectra were recorded at 470.4 MHz using benzotrifluoride ($C_6H_5CF_3$) as the external standard. The terms m, s, d, t, and q. represent multiplet, singlet, doublet, triplet, and quadruplet, respectively. Commercial grade reagents and solvents were used without further purification. GC spectra were recorded with a Varian 450-GC. Mass analysis was performed using AB SCIEX 4800 PLUS MALDI-TOF/TOF analyzer.

General Procedure for Oxidation of Alcohol to Aldehyde (A). A 10-mL two necked, round-bottomed flask equipped with a magnetic stirring bar, a reflux condenser, and a glass stopper was charged with the corresponding primary alcohol (1 mmol) and graphene oxide (5 wt%) in 1,4-dioxane (2 mL). After sonication for 1 min, 67% nitric acid (2 mmol) was added dropwise into the reaction mixture, and the resulting mixture allowed to stir at 90 °C for 2-3 h under open-air conditions. After reaction completion, the resulting mixture was diluted with dichloromethane and filtered through a 0.45 µm syringe filter. The resulting solution was rinsed with deionised water and brine, dried over Na_2SO_4, and concentrated under reduced pressure. The residue was purified by column chromatography on silica gel with diethyl ether and pentane as the eluting solvents to afford the corresponding aldehyde.

General Procedure for the Variant of Knoevenagel Condensation Reaction between Alcohol and Malonate (B). A 10-mL two necked, round-bottomed flask equipped with a magnetic stirring bar, a reflux condenser, and a glass stopper was added oven-dried 4 Å molecular sieves. The flask was then flame-dried under vacuum and subsequently filled with argon. The flask was charged with piperidine (20 mol%), 67% nitric acid (2.2 mmol), and 1,4-dioxane/DMF (v/v = 1:3) and stirred for 10 min. Primary alcohol (1 mmol), malonate (1 mmol), graphene oxide (5 wt%), and 1,4-dioxane (2 mL) were then added to the reaction mixture. After sonication for 1 min, the resulting mixture was stirred at 90 °C for 24 h under 1 atm of O_2. Upon completion of the reaction, the mixture was cooled to room temperature, filtered through 0.45 µm membrane filter paper, and washed with deionised water and ethyl acetate. The organic layers were combined, dried over Na_2SO_4, and concentrated under reduced pressure. The residue was purified by flash column chromatography on silica gel with ethyl acetate and hexanes as the eluting solvents to give the corresponding Knoevenagel condensation product.

General Procedure for Oxidation of Alcohol to Carboxylic acid (C). Primary alcohol (1 mmol), graphene oxide (5 wt%), and 1,4-dioxane (2 mL) were added to a glass vessel. After sonication for 1 min, 67% nitric acid (2 mmol) was added dropwise into the reaction mixture, and then the glass vessel was placed into an autoclave. The autoclave was flushed three times with O_2 (0.2 MPa), then pressurized to 0.6 MPa. The autoclave was placed into a preheated oil bath at 90 °C for the desired reaction time, then cooled to room temperature, and carefully depressurised. The reaction mixture was diluted with ethyl acetate and filtered through a 0.45 µm syringe filter. The resulting solution was rinsed with deionised water and brine, dried over

Na_2SO_4, and concentrated under reduced pressure. The residue was purified by column chromatography on silica gel with ethyl acetate as the eluting solvent to afford the corresponding carboxylic acid.

Synthesis of Graphene Oxide: Graphene oxide (GO) was synthesised from graphite powder using a modified Hummer's method.[1] Concentrated H_2SO_4 (75 mL) was added to a mixture of graphite flakes (2.5 g) and $NaNO_3$ (1.25 g), and the mixture was cooled to 0 °C. $KMnO_4$ (20.0 g) was added slowly in portions to keep the reaction temperature below 20 °C. The reaction was warmed to 35 °C and stirred for 45 min, at which time water (50 mL) was added slowly, producing a large exotherm to 98 °C. External heating was introduced to maintain the reaction temperature at 98 °C for 1h, then the heat was removed and the reaction was cooled using a water bath for 10 min. Additional water (100 mL) and 30% H_2O_2 (15 mL) were added, producing another exotherm. After air cooling, the resulting mixture was washed with H_2O respectively, followed by filtration (membrane filter, 0.45 μm) and the graphene oxide sheets were dried at 60 °C under vacuum for 1 d.

Graphene Oxide by Lithium Aluminum Hydride ($LiAlH_4$): Dry graphite oxide powder (50 mg) was dispersed in dry THF (30 mL) and ultrasonicated (150 W) for 3 h. $LiAlH_4$ (190 mg, 5 mmol) dispersed in dry THF (10 mL) was added dropwise at 0 °C until bubbling was no longer observed. The mixture was then transferred into a solution of $LiAlH_4$ (760 mg, 20 mmol) in dry THF (20 mL) dropwise at 0 °C. The reaction mixture was then stirred at reflux for 24 h. The mixture was quenched with a saturated solution of Na_2SO_4, followed by 1 M hydrochloric acid, at 0 °C until a clear solution was obtained. The mixture was filtered (membrane filter, 0.45 μm), washed repeatedly with deionised water, and then dried at 60 °C under vacuum for 1 d.[2]

Characterization Data for Products

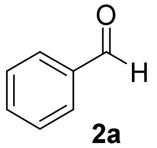

2a **Benzaldehyde (Table 4, entry 1):** The title compound was synthesized according to general procedure A. The physical and spectral data were identical to those previously reported for this compound.[3] **^{1}H NMR** (500 MHz, $CDCl_3$) δ 10.03 (s, 1H), 7.90 (d, J = 6.9 Hz, 2H), 7.64 (t, J = 7.4 Hz, 1H), 7.54 (t, J = 7.7 Hz, 2H) ppm; **^{13}C NMR** (125 MHz, $CDCl_3$) δ 192.4, 136.4, 134.5, 129.7, 129.0 ppm.

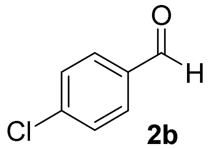

2b **4-Chlorobenzaldehyde (Table 4, entry 2):** The title compound was synthesized according to general procedure A. The physical and spectral data were identical to those previously reported for this compound.[3] **^{1}H NMR** (500 MHz, $CDCl_3$) δ 9.99 (s, 1H), 7.83 (d, J = 8.4 Hz, 2H), 7.52 (d, J = 8.4 Hz, 2H) ppm; **^{13}C NMR** (125 MHz, $CDCl_3$) δ 190.9, 140.9, 134.7, 130.9, 129.4 ppm.

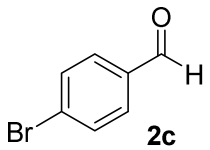

2c **4-Bromobenzaldehyde (Table 4, entry 3):** The title compound was synthesized according to general procedure A. The physical and spectral data were identical to those previously reported for this compound.[3] **^{1}H NMR** (500 MHz, $CDCl_3$) δ 9.98 (s, 1H), 7.76 (d, J = 8.5 Hz, 2H), 7.69 (d, J = 8.4 Hz, 2H) ppm; **^{13}C NMR** (125 MHz, $CDCl_3$) δ 191.1, 135.0, 132.4, 130.9, 129.8 ppm.

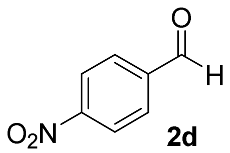

2d **4-Nitrobenzaldehyde (Table 4, entry 4):** The title compound was synthesized according to general procedure A. The physical and spectral data were identical to those previously reported for this compound.[3] **^{1}H NMR** (500 MHz, $CDCl_3$) δ 10.19 (s, 1H), 8.42 (d, J = 8.5 Hz, 2H), 8.11 (d, J = 8.5 Hz, 2H) ppm; **^{13}C NMR** (125 MHz, $CDCl_3$) δ 190.3, 151.1, 140.0, 130.5, 124.3 ppm.

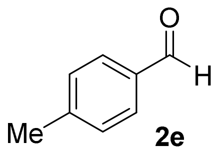

2e **4-Methylbenzaldehyde (Table 4, entry 5):** The title compound was synthesized according to general procedure A. The physical and spectral data were identical to those previously reported for this compound.[3] **^{1}H NMR** (500 MHz, $CDCl_3$) δ 9.89 (s, 1H),

7.69 (d, J = 7.9 Hz, 2H), 7.22 (d, J = 7.9 Hz, 2H), 2.32 (s, 3H) ppm; **^{13}C NMR** (125 MHz, $CDCl_3$) δ 191.4, 145.1, 134.1, 129.5, 129.5, 21.4 ppm.

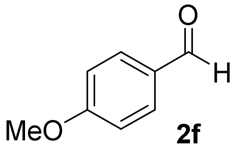

2f **4-Methoxybenzaldehyde (Table 4, entry 6):** The title compound was synthesized according to general procedure A. The physical and spectral data were identical to those previously reported for this compound.[3] **^{1}H NMR** (500 MHz, $CDCl_3$) δ 9.84 (s, 1H), 7.79 (d, J = 8.7 Hz, 2H), 6.96 (d, J = 8.7 Hz, 2H), 3.81 (s, 3H) ppm; **^{13}C NMR** (125 MHz, $CDCl_3$) δ 190.4, 164.4, 131.7, 129.8, 114.1, 55.3 ppm.

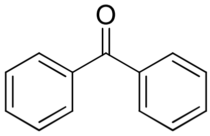

8 **Benzophenone (8):** The title compound was synthesized according to general procedure A. The physical and spectral data were identical to those previously reported for this compound.[4] **^{1}H NMR** (500 MHz, $CDCl_3$) δ 7.81–7.79 (d, J = 7.93 Hz, 4H,), 7.59–7.56 (t, J = 7.13 Hz, 2H), 7.48–7.45 (t, J = 7.4 Hz, 2H) ppm; **^{13}C NMR** (125 MHz, $CDCl_3$) δ 196.8, 137.6, 132.4, 130.1, 128.3 ppm.

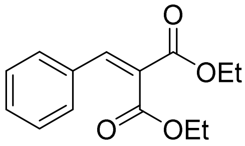

5a **Diethyl 2-benzylidenemalonate (5a):** The title compound was synthesized according to general procedure B. The physical and spectral data were identical to those previously reported for this compound.[5] Colorless oil; yield 72%; **^{1}H NMR** (500 MHz, $CDCl_3$) δ 7.74 (s, 1H), 7.47–7.45 (m, 2H), 7.40–7.37 (m, 3H), 4.36–4.29 (m, 4H), 1.34 (t, J = 7.1 Hz, 3H), 1.29 (t, J = 7.1 Hz, 3H) ppm; **^{13}C NMR** (125 MHz, $CDCl_3$) δ 166.7, 164.1, 142.1, 132.9, 130.5, 129.4, 128.8, 126.3, 61.7, 61.6, 14.1, 13.9 ppm; IR (Neat) ν_{max} 1064, 1201, 1259, 1726 cm^{-1}.

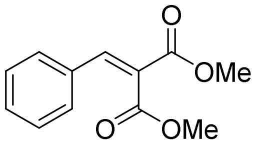

5b **Dimethyl 2-benzylidenemalonate (5b):** The title compound was synthesized according to general procedure B. The physical and spectral data were identical to those previously reported for this compound.[6] White solid; yield 65%; **^{1}H NMR** (500 MHz, $CDCl_3$) δ 7.78 (s, 1H), 7.44–7.38 (m, 5H), 3.85 (s, 6H) ppm; **^{13}C NMR** (125 MHz, $CDCl_3$) δ 167.1, 164.5, 142.9, 132.7, 130.7, 129.4, 128.9, 125.4, 52.7, 52.7 ppm; m.p. = 35-38°C; IR (Neat) ν_{max} 1064, 1201, 1222, 1265, 1436, 1730 cm^{-1}.

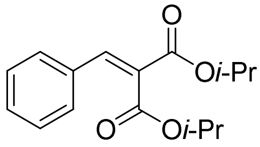

5c **Diisopropyl 2-benzylidenemalonate (5c):** The title compound was synthesized according to general procedure B. The physical and spectral data were identical to those previously reported for this compound.[7] Colorless oil; yield 68%; ^{1}**H NMR** (500 MHz, $CDCl_3$) δ 7.69 (s, 1H), 7.48–7.46 (m, 2H), 7.36–7.35 (m, 3H), 5.25 (heptet, J = 6.3 Hz, 1H), 5.15 (heptet, J = 6.3 Hz, 1H), 1.31 (d, J = 6.3 Hz, 6H), 1.29 (d, J = 6.3 Hz, 6H) ppm; ^{13}**C NMR** (125 MHz, $CDCl_3$) δ 166.2, 163.6, 141.3, 133.0, 130.3, 129.4, 128.7, 127.1, 69.2, 69.1, 21.7, 21.4 ppm; IR (Neat)ν_{max} 1054, 1107, 1199, 1222, 1261, 1722 cm^{-1}.

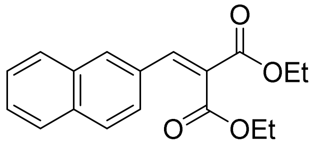

5d **Diethyl 2-(naphthalen-2-ylmethylene)malonate (5d):** The title compound was synthesized according to general procedure B. White solid; yield 68%; ^{1}**H NMR** (500 MHz, $CDCl_3$) δ 7.96 (s, 1H), 7.90 (s, 1H), 7.84–7.80 (m, 3H), 7.54–7.48 (m, 3H), 4.38 (q, J = 7.1 Hz, 2H), 4.33 (q, J = 7.1 Hz, 2H), 1.35 (t, J = 7.1 Hz, 3H), 1.30 (t, J = 7.1 Hz, 3H) ppm; ^{13}**C NMR** (125 MHz, $CDCl_3$) δ 166.8, 164.2, 142.2, 134.0, 133.0, 130.9, 130.4, 128.7, 128.5, 127.7, 127.6, 126.7, 126.3, 125.2, 61.7, 61.7, 14.2, 13.9 ppm. HRMS (MALDI-TOF) m/z $[M + Na]^+$ calcd for $C_{18}H_{18}NaO_4$ 321.1098, found 321.1092; m.p. = 37–39 °C; IR (Neat) ν_{max} 1064, 1176, 1211, 1242, 1436, 1627, 1724 cm^{-1}.

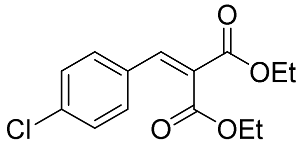

5e **Diethyl 2-(4-chlorobenzylidene)malonate (5e):** The title compound was synthesized according to general procedure B. The physical and spectral data were identical to those previously reported for this compound.[8] Colorless oil; yield 72%; ^{1}**H NMR** (500 MHz, $CDCl_3$) δ 7.67 (s, 1H), 7.40–7.34 (m, 4H), 4.36–4.29 (m, 4H), 1.33 (t, J = 7.1 Hz, 3H), 1.30 (t, J = 7.1 Hz, 3H) ppm; ^{13}**C NMR** (125 MHz, $CDCl_3$) δ 166.41, 163.91, 140.62, 136.61, 131.40, 130.67, 129.10, 126.87, 61.83, 61.77, 14.13, 13.91 ppm; IR (Neat) ν_{max} 1014, 1064, 1211, 1255, 1436, 1629, 1728 cm^{-1}.

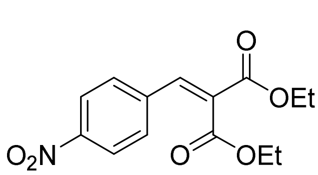

5f **Diethyl 2-(4-nitrobenzylidene)malonate (5f):** The title compound was synthesized according to general procedure B. The physical and spectral data were identical to those previously reported for this compound.[9] Pale yellow solid; yield 77%; ^{1}**H**

NMR (500 MHz, $CDCl_3$) δ 8.24 (d, J = 8.8 Hz, 2H), 7.76 (s, 1H), 7.62 (d, J = 8.6 Hz, 2H), 4.34 (q, J = 7.1 Hz, 4H) 1.36 (t, J = 7.1 Hz, 3H), 1.29 (t, J = 7.1 Hz, 3H) ppm; ^{13}**C NMR** (125 MHz, $CDCl_3$) δ 165.6, 163.3, 148.4, 139.2, 139.1, 130.0, 129.9, 123.9, 62.2, 62.1, 14.1, 13.9 ppm; m.p. = 90–91 °C; IR (Neat) ν_{max} 856, 1012, 1064, 1201, 1215, 1259, 1344, 1519, 1716, 1728 cm^{-1}.

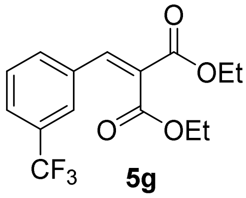

5g **Diethyl 2-(3-(trifluoromethyl)benzylidene)malonate (5g):** The title compound was synthesized according to general procedure B. White solid; yield 64%; ^{1}**H NMR** (500 MHz, $CDCl_3$) δ 7.75 (s, 1H), 7.71 (s, 1H), 7.66–7.62 (m, 2H), 7.54–7.50 (m, 1H), 4.36–4.31 (m, 4H), 1.35 (t, J = 7.1 Hz, 3H), 1.29 (t, J = 7.1 Hz, 3H) ppm; ^{13}**C NMR** (125 MHz, $CDCl_3$) δ 166.0, 163.6, 140.1, 133.7, 132.4, 131.3 (q, $J_{C\text{-}F}$ = 32.4 Hz), 129.3, 128.3, 126.8 (q, $J_{C\text{-}F}$ = 3.7 Hz), 125.8 (q, $J_{C\text{-}F}$ = 3.8 Hz), 123.6 (q, $J_{C\text{-}F}$ = 270.8 Hz), 61.9, 61.9, 14.1, 13.8 ppm. ^{19}**F NMR** (470.4 MHz, $CDCl_3$) δ -63.07 ppm. HRMS (MALDI-TOF) m/z $[M + Na]^+$ calcd for $C_{15}H_{15}F_3NaO_4$ 339.0815, found 339.0818; m.p. = 29–31 °C; IR (Neat) ν_{max} 1066, 1128, 1168, 1193, 1255, 1330, 1637, 1728 cm^{-1}.

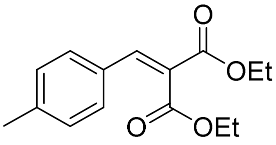

5h **Diethyl 2-(4-methylbenzylidene)malonate (5h):** The title compound was synthesized according to general procedure B. The physical and spectral data were identical to those previously reported for this compound.[10] Colorless solid; yield 64%; ^{1}**H NMR** (500 MHz, $CDCl_3$) δ 7.70 (s, 1H), 7.36 (d, J = 8.1 Hz, 2H), 7.18 (d, J = 8.1Hz, 2H), 4.35 (q, J = 7.1 Hz, 2H), 4.30 (q, J = 7.1 Hz, 2H), 2.37 (s, 3H), 1.33 (t, J = 7.1 Hz, 3H), 1.30 (t, J = 7.1 Hz, 3H) ppm; ^{13}**C NMR** (125 MHz, $CDCl_3$) δ 166.9, 164.3, 142.1, 141.1, 130.0, 129.6, 129.5, 125.1, 61.6, 61.5, 21.5, 14.1, 13.9 ppm; m.p. = 47–49 °C; IR (Neat) ν_{max} 813, 1064, 1184, 1205, 1257, 1608, 1629, 1726 cm^{-1}.

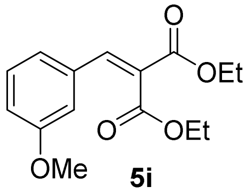

5i **Diethyl 2-(3-methoxybenzylidene)malonate (5i):** The title compound was synthesized according to general procedure B. Colorless oil; yield 66%; ^{1}**H NMR** (500 MHz, $CDCl_3$) δ 7.70 (s, 1H), 7.30–7.27 (m, 1H), 7.05–7.04 (m, 1H), 6.99 (br, 1H), 6.95–6.93 (m,1H), 4.36–4.28 (m, 4H), 3.79 (s, 3H), 1.33 (t, J = 7.1 Hz, 3H), 1.29 (t, J = 7.1 Hz, 3H) ppm; ^{13}**C NMR** (125 MHz, $CDCl_3$) δ 166.6, 164.0, 159.7, 141.9, 134.1, 129.8, 126.5, 121.9, 116.5, 114.3, 61.7, 61.6, 55.2, 14.1, 13.9 ppm. HRMS (MALDI-TOF) m/z $[M + Na]^+$ calcd for $C_{15}H_{18}NaO_5$

301.1047, found 301.1047; IR (Neat) ν_{max} 752, 1066, 1236, 1276, 1728 cm^{-1}.

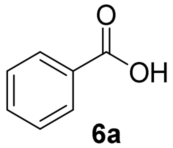

6a **Benzoic acid (Table 4, entry 7):** The title compound was synthesized according to general procedure C. The physical and spectral data were identical to those previously reported for this compound.[11] **^{1}H NMR** (500 MHz, DMSO-d_6) δ 12.99 (s, 1H), 7.97–7.96 (m, 2H), 7.65–7.62 (m, 1H), 7.53–7.50 (m, 2H) ppm; **^{13}C NMR** (125 MHz, DMSO-d_6) δ 167.8, 133.3, 131.2, 129.7, 129.0 ppm.

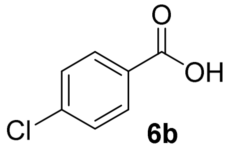

6b **4-Chlorobenzoic acid (Table 4, entry 8):** The title compound was synthesized according to general procedure C. The physical and spectral data were identical to those previously reported for this compound.[11] **^{1}H NMR** (500 MHz, DMSO-d_6) δ 13.21 (s, 1H), 7.94 (d, J = 8.5 Hz, 2H), 7.56 (d, J = 8.5 Hz, 2H) ppm; **^{13}C NMR** (125 MHz, DMSO-d_6) δ 166.4, 137.8, 131.1, 129.6, 128.7 ppm.

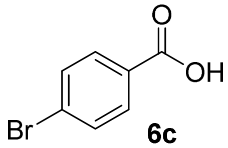

6c **4-Bromobenzoic acid (Table 4, entry 9):** The title compound was synthesized according to general procedure C. The physical and spectral data were identical to those previously reported for this compound.[11] **^{1}H NMR** (500 MHz, DMSO-d_6) δ 13.21 (s, 1H), 7.87 (d, J = 8.4 Hz, 2H), 7.72 (d, J = 8.4 Hz, 2H) ppm; **^{13}C NMR** (125 MHz, DMSO-d_6) δ 166.6, 131.7, 131.3, 130.0, 126.9 ppm.

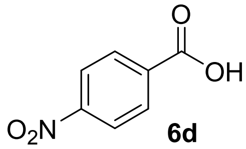

6d **4-Nitrobenzoic acid (Table 4, entry 10):** The title compound was synthesized according to general procedure C. The physical and spectral data were identical to those previously reported for this compound.[11] **^{1}H NMR** (500 MHz, DMSO-d_6) δ 13.70 (s, 1H), 8.33 (d, J = 8.9 Hz, 2H), 8.18 (d, J = 8.9 Hz, 2H) ppm; **^{13}C NMR** (125 MHz, DMSO-d_6) δ 165.8, 150.0, 136.4, 130.7, 123.7 ppm.

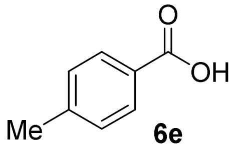

6e **4-Methylbenzoic acid (Table 4, entry 11):** The title compound was synthesized according to general procedure C. The physical and spectral data were identical to those previously reported for this compound.[11] **^{1}H NMR** (500 MHz, DMSO-d_6) δ 12.81 (s,

1H), 7.84 (d, J = 8.1 Hz, 2H), 7.31 (d, J = 8.0 Hz, 2H), 2.37 (s, 3H) ppm; **^{13}C NMR** (125 MHz, DMSO-d_6) δ 167.3, 143.0, 129.3, 129.1, 128.0, 21.1 ppm.

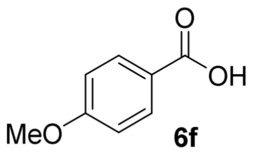

6f

4-Methoxybenzoic acid (Table 4, entry 12): The title compound was synthesized according to general procedure C. The physical and spectral data were identical to those previously reported for this compound.[11] **^{1}H NMR** (500 MHz, DMSO-d_6) δ 12.65 (s, 1H), 7.90 (d, J = 8.7 Hz, 2H), 7.02 (d, J = 8.7 Hz, 2H), 3.82 (s, 3H) ppm; **^{13}C NMR** (125 MHz, DMSO-d_6) δ 167.0, 162.8, 131.3, 122.9, 113.8, 55.4 ppm.

References

1. Hummers, W. S. & Offeman, R. E. Preparation of Graphitic Oxide. *J. Am. Chem. Soc.* **80,** 1339 (1958).
2. Ambrosi, A., Chua, C. K., Bonanni, A. & Pumera, M. Lithium Aluminum Hydride as Reducing Agent for Chemically Reduced Graphene Oxides. *Chem. Mater.* **24**, 2292-2298 (2012).
3. Iinuma, M., Moriyama, K. & Togo, H. Various oxidative reactions with novel ion-supported (diacetoxyiodo)benzenes. *Tetrahedron* **69**, 2961-2970 (2013).
4. Yuan, Y., Shi, X. & Liu, W. Transition-Metal-Free, Chemoselective Aerobic Oxidations of Sulfides and Alcohols with Potassium Nitrate and Pyridinium Tribromide or Bromine. *Synlett* **4**, 559-564 (2011).
5. Richter, H. & Mancheño, O. G. Dehydrogenative Functionalization of C(sp^3)-H Bonds Adjacent to a Heteroatom Mediated by Oxoammonium Salts. *Eur. J. Org. Chem*. 4460-4467 (2010).
6. Qiu, R. *et al.* Facile separation catalyst system: direct diastereoselective synthesis of (*E*)-α,β-unsaturated ketones catalyzed by an air-stable Lewis acidic/basic bifunctional organobismuth complex in ionic liquids. *Green Chem.* **12**, 1767-1771 (2010).
7. Yamashita, K., Tanaka, T. & Hayashi, M. Use of isopropyl alcohol as a solvent in Ti(O-*i*-Pr)$_4$-catalyzed Knöevenagel reactions. *Tetrahedron* **61**, 7981-7985 (2005).
8. Moussaoui, Y. & Ben Salem, R. C. R. Catalyzed Knoevenagel reactions on inorganic solid supports: Application to the synthesis of coumarine compounds. *Chim.* **10**, 1162-1169 (2007).
9. Leelavathi, P. & Kumar, S. R. Niobium (V) chloride catalyzed Knoevenagel condensation: An efficient protocol for the preparation of electrophilic alkenes. *J. Mol. Catal. A-Chem.* **240**, 99-102 (2005).
10. Kaumanns, O., Lucius, R. & Mayr, H. Determination of the Electrophilicity Parameters of Diethyl Benzylidenemalonates in Dimethyl Sulfoxide: Reference Electrophiles for Characterizing Strong Nucleophiles. *Chem-Eur. J.* **14**, 9675-9682 (2008).
11. Kim, S. M., Kim, D. W. & Yang, J. W. Transition-Metal-Free and Chemoselective NaO^{t}Bu-O_2-Mediated Oxidative Cleavage Reactions of *vic*-1,2-Diols to Carboxylic Acids and Mechanistic Insight into the Reaction Pathways. *Org. Lett.* **16**, 2876-2879 (2014).

^{1}H NMR and ^{13}C NMR Spectra of Products

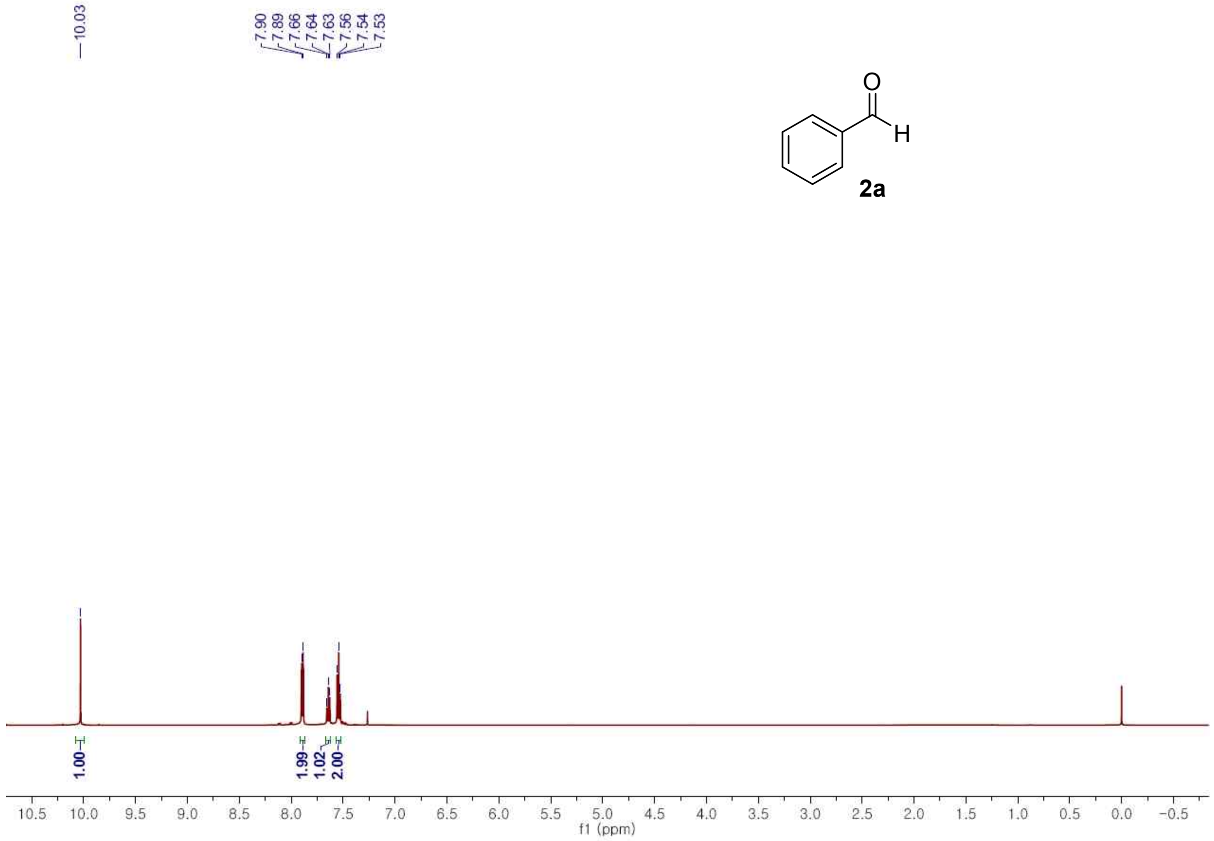

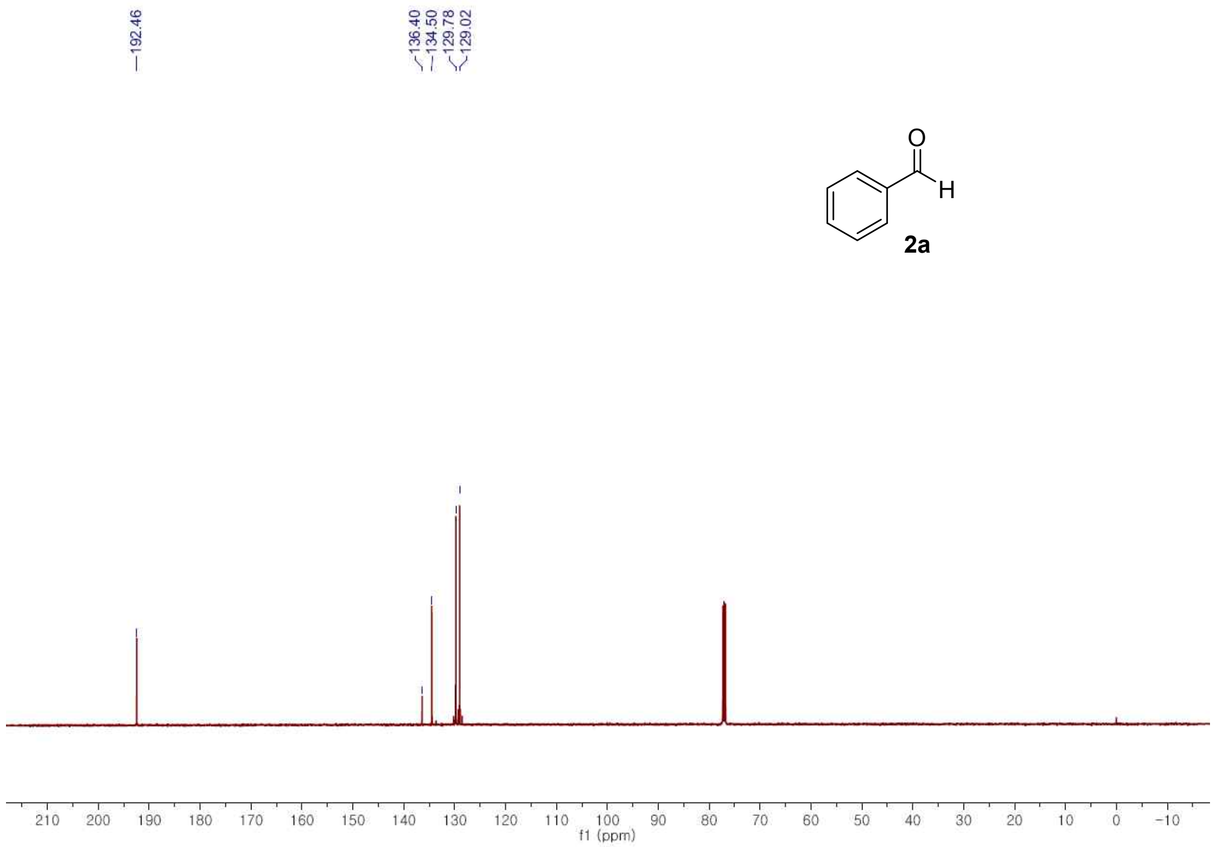

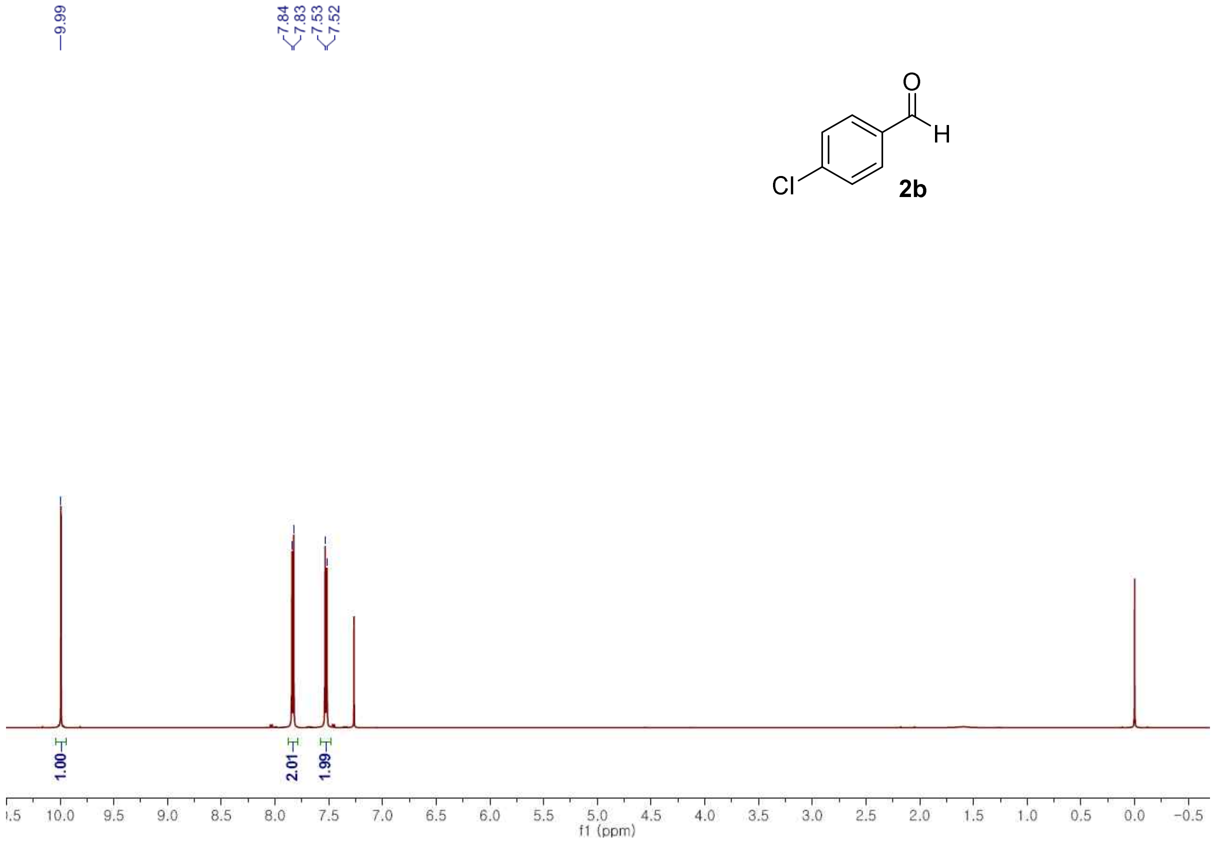

9.99
7.84
7.83
7.53
7.52
O
H
Cl
2b
1.00
2.01
1.99
f1 (ppm)

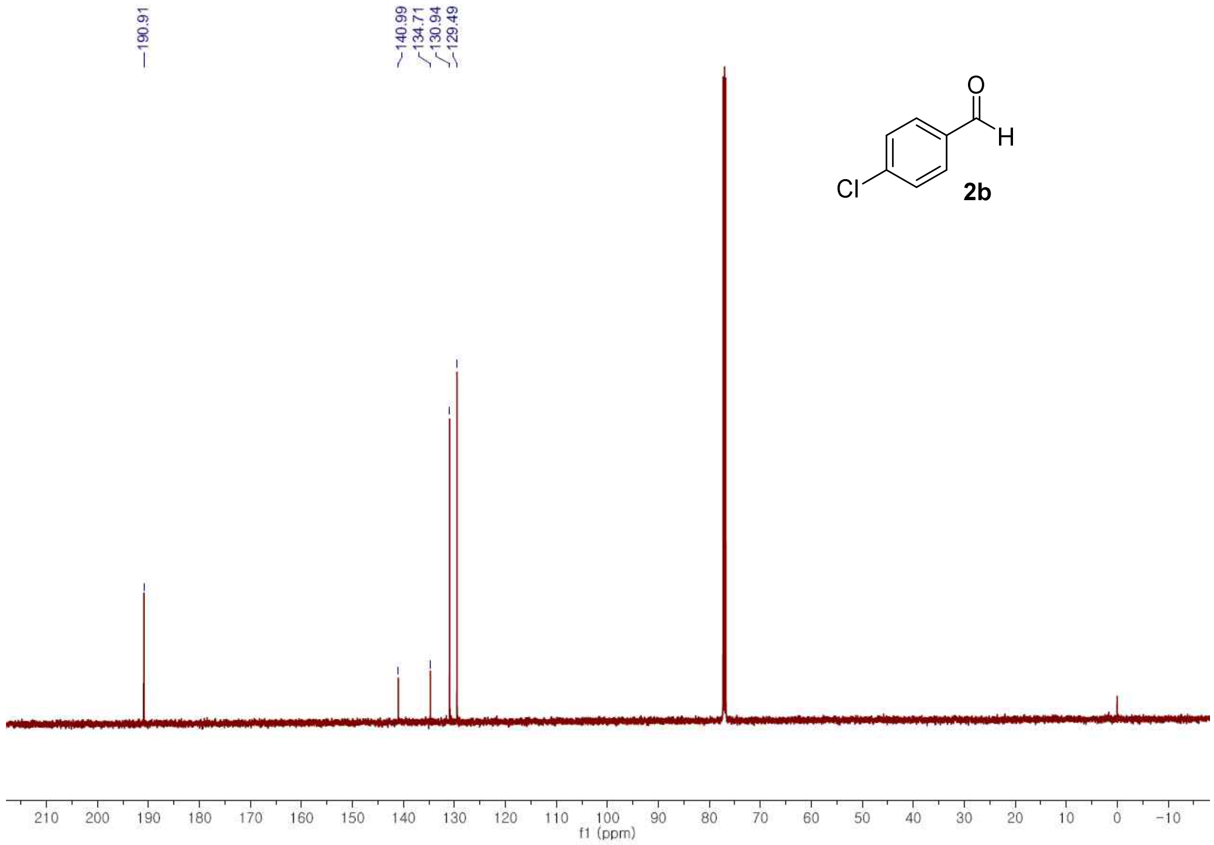

190.91
140.99
134.71
130.94
129.49
O
H
Cl
2b
f1 (ppm)

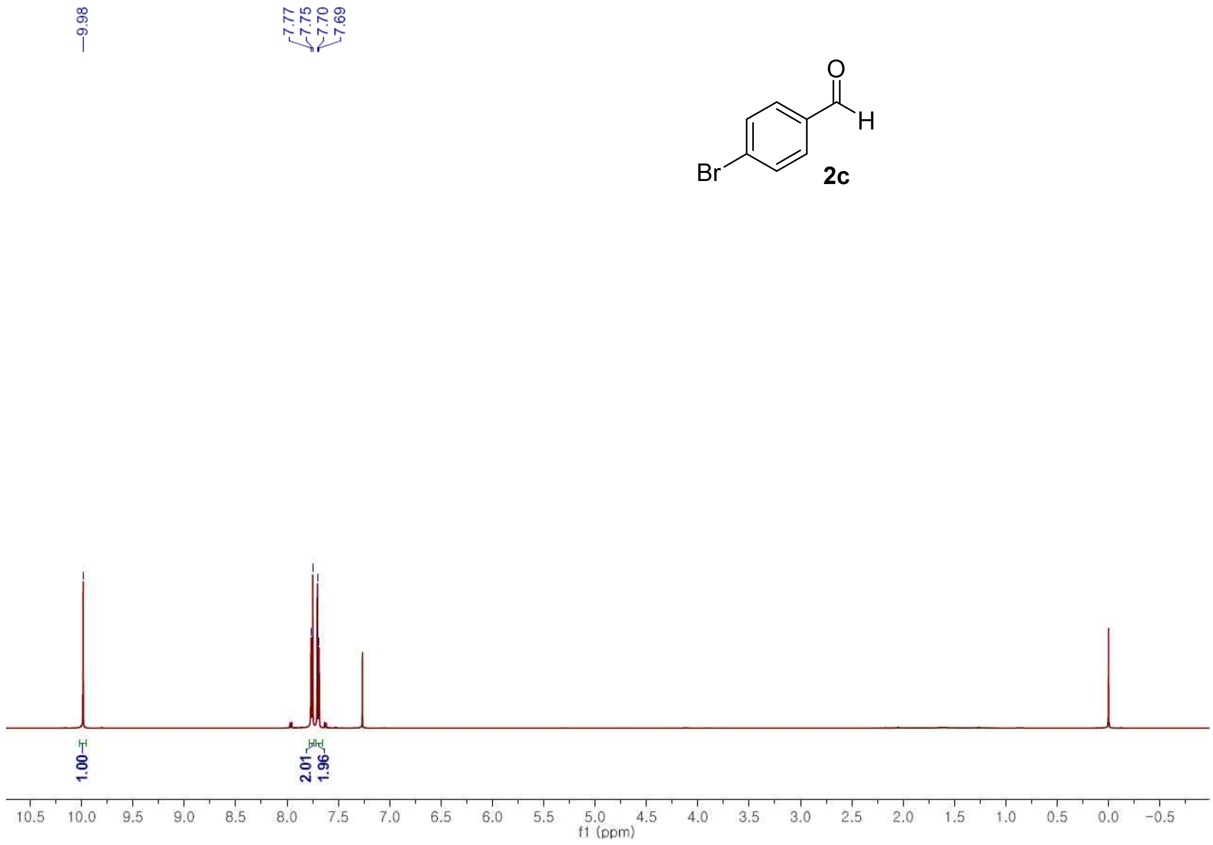

9.98
7.77
7.75
7.70
7.69
Br
O
H
2c
1.00
2.01
1.96
10.5
10.0
9.5
9.0
8.5
8.0
7.5
7.0
6.5
6.0
5.5
5.0
4.5
4.0
3.5
3.0
2.5
2.0
1.5
1.0
0.5
0.0
-0.5
f1 (ppm)

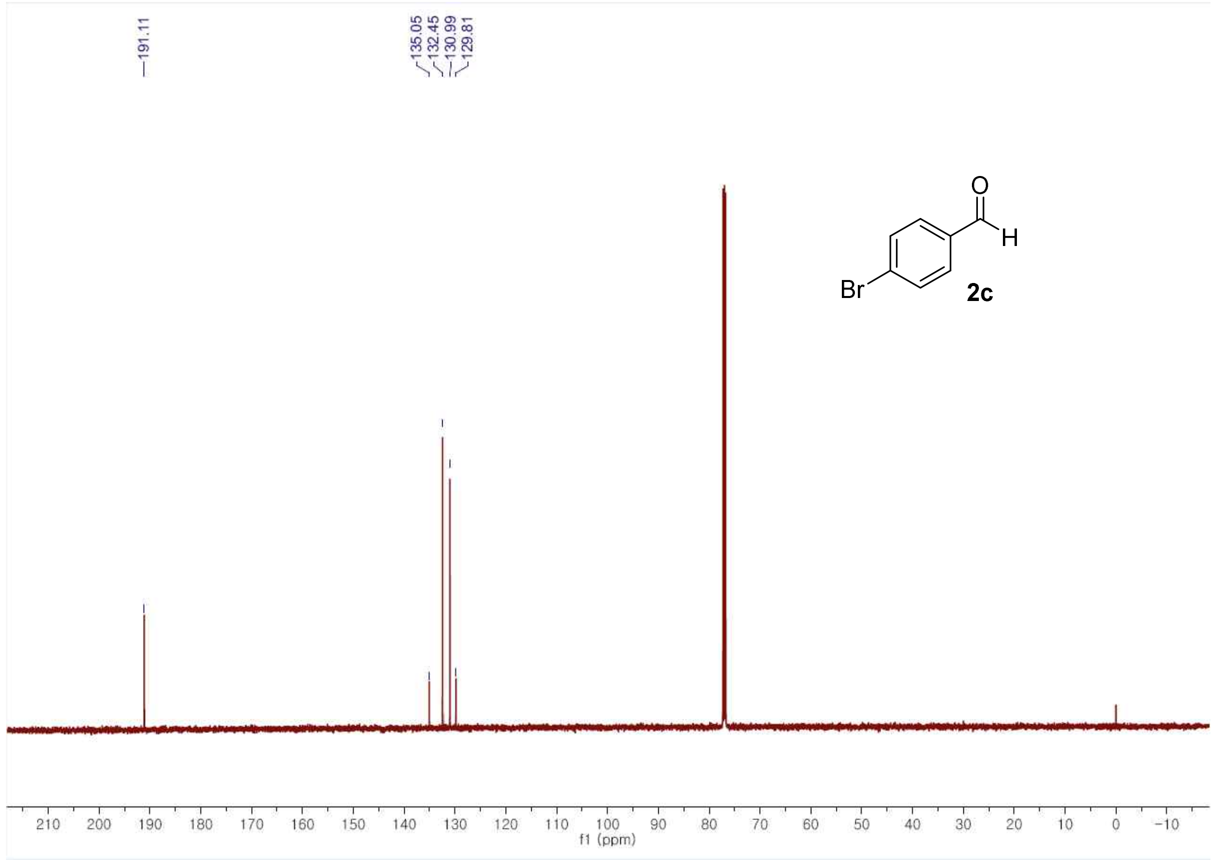

191.11
135.05
132.45
130.99
129.81
Br
O
H
2c
210
200
190
180
170
160
150
140
130
120
110
100
90
80
70
60
50
40
30
20
10
0
-10
f1 (ppm)

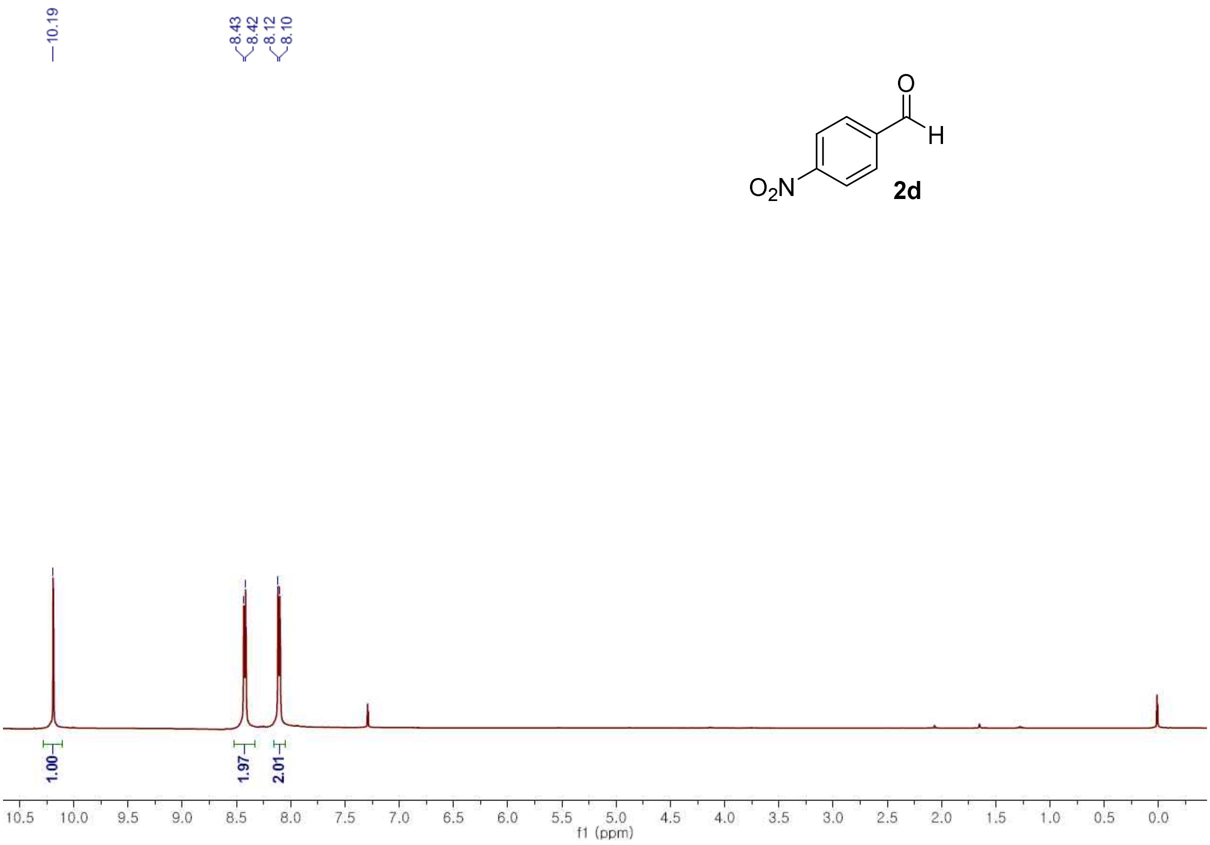
10.19
8.43
8.42
8.12
8.10
O
H
O2N
2d
1.00
1.97
2.01
10.5 10.0 9.5 9.0 8.5 8.0 7.5 7.0 6.5 6.0 5.5 5.0 4.5 4.0 3.5 3.0 2.5 2.0 1.5 1.0 0.5 0.0
f1 (ppm)

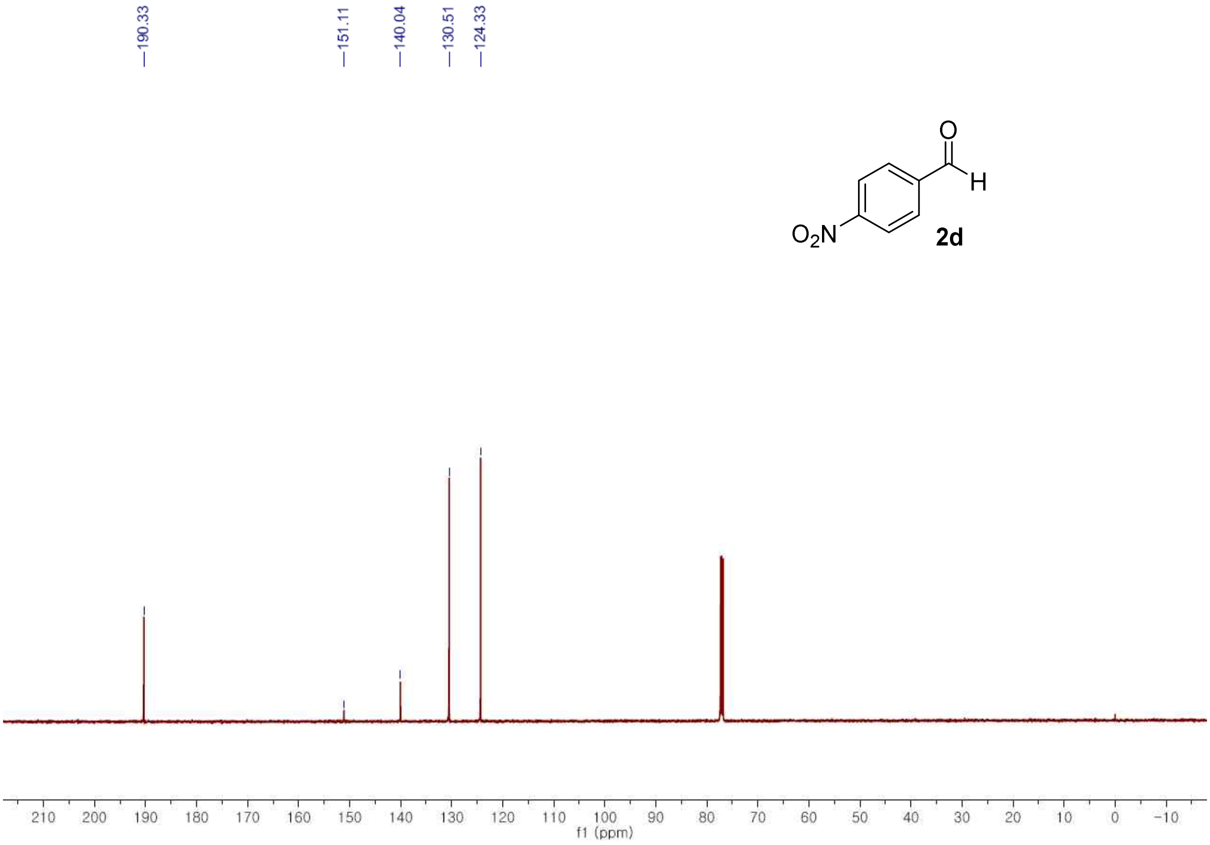
190.33
151.11
140.04
130.51
124.33
O
H
O2N
2d
210 200 190 180 170 160 150 140 130 120 110 100 90 80 70 60 50 40 30 20 10 0 -10
f1 (ppm)

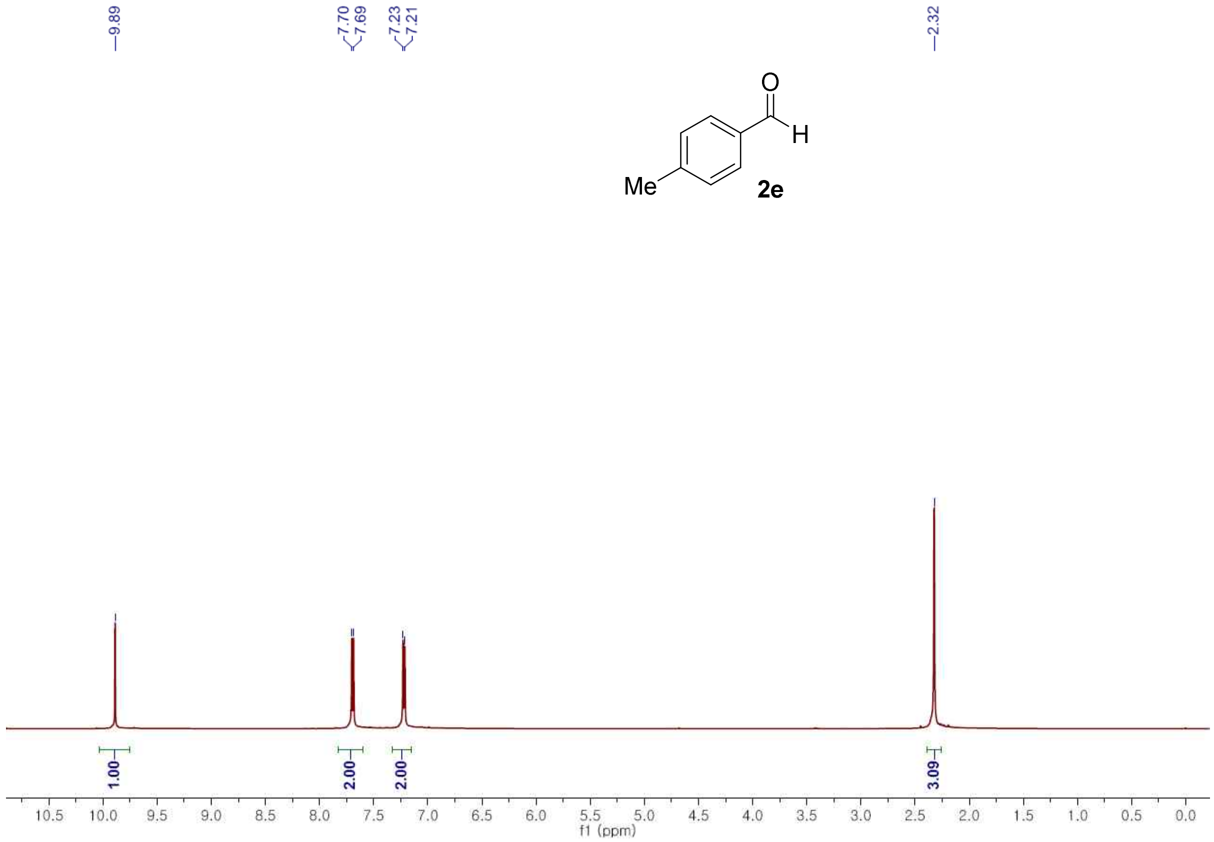
9.89
7.70
7.69
7.23
7.21
2.32
O
H
Me
2e
1.00
2.00
2.00
3.09
10.5 10.0 9.5 9.0 8.5 8.0 7.5 7.0 6.5 6.0 5.5 5.0 4.5 4.0 3.5 3.0 2.5 2.0 1.5 1.0 0.5 0.0
f1 (ppm)

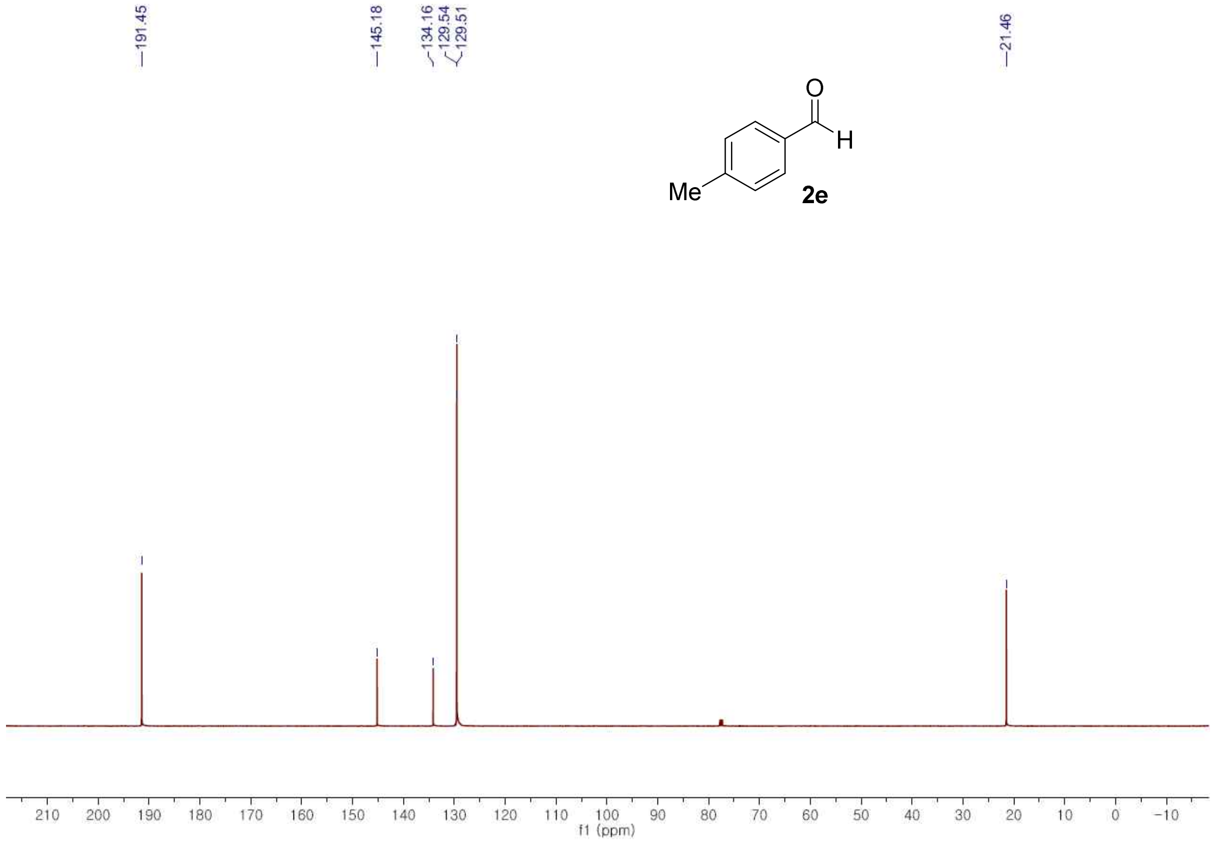
191.45
145.18
134.16
129.54
129.51
21.46
O
H
Me
2e
210 200 190 180 170 160 150 140 130 120 110 100 90 80 70 60 50 40 30 20 10 0 -10
f1 (ppm)

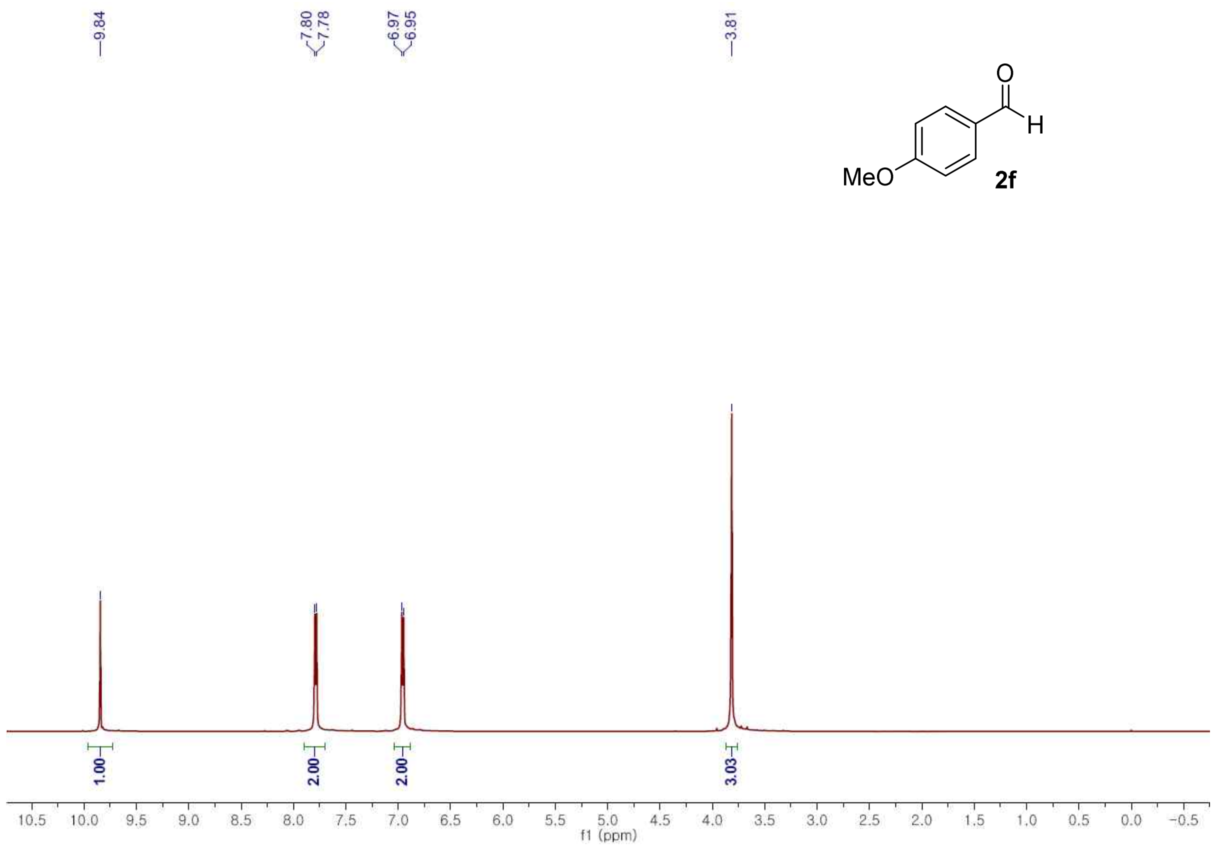
9.84
7.80
7.78
6.97
6.95
3.81
MeO
O
H
2f
1.00
2.00
2.00
3.03
10.5 10.0 9.5 9.0 8.5 8.0 7.5 7.0 6.5 6.0 5.5 5.0 4.5 4.0 3.5 3.0 2.5 2.0 1.5 1.0 0.5 0.0 -0.5
f1 (ppm)

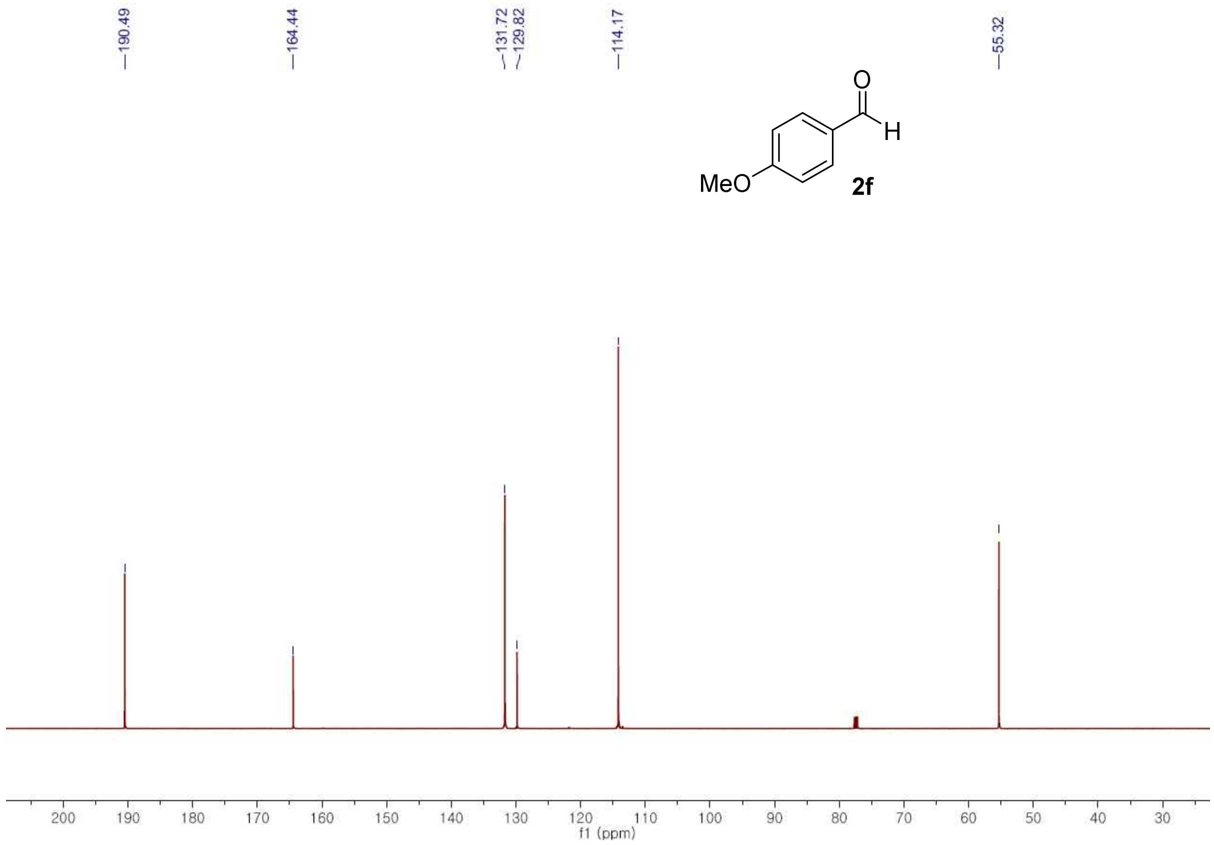
190.49
164.44
131.72
129.82
114.17
55.32
MeO
O
H
2f
200 190 180 170 160 150 140 130 120 110 100 90 80 70 60 50 40 30
f1 (ppm)

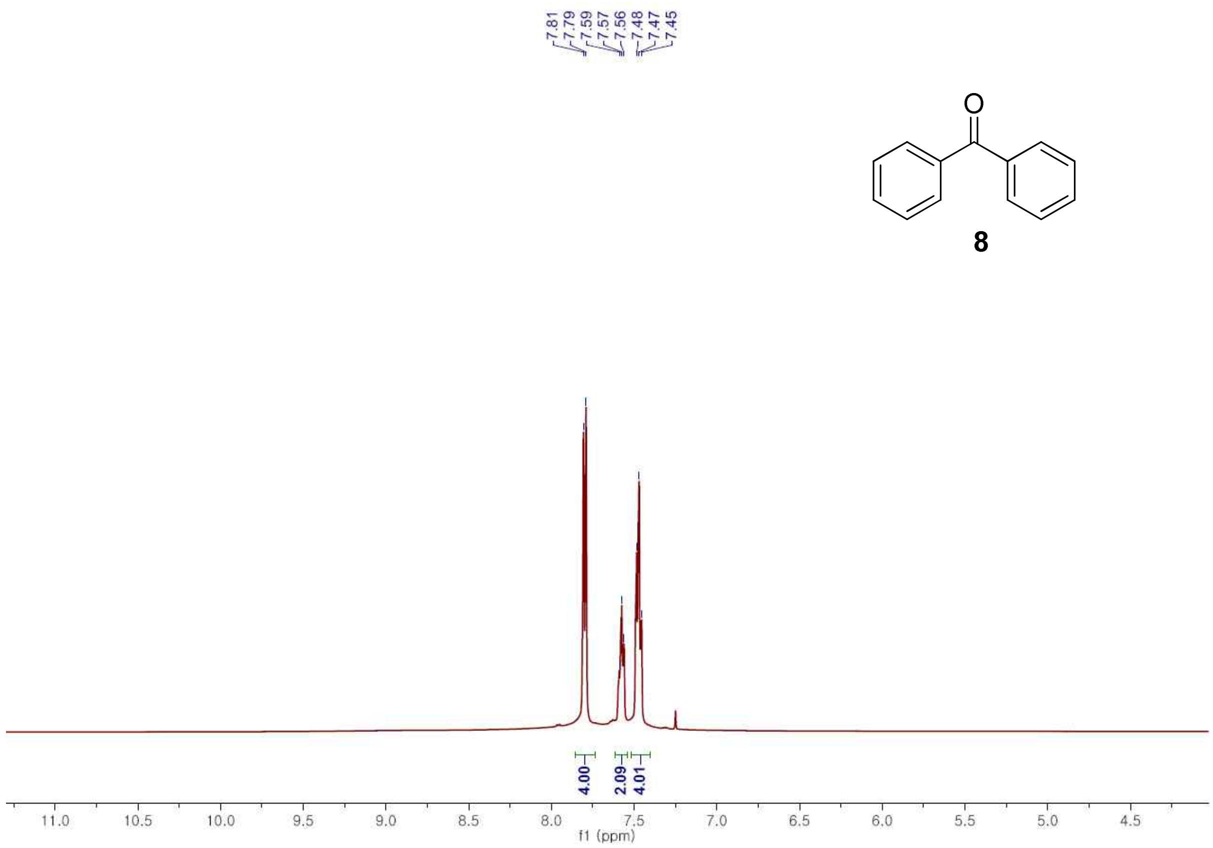
7.81
7.79
7.59
7.57
7.56
7.48
7.47
7.45
O
8
4.00
2.09
4.01
11.0 10.5 10.0 9.5 9.0 8.5 8.0 7.5 7.0 6.5 6.0 5.5 5.0 4.5
f1 (ppm)

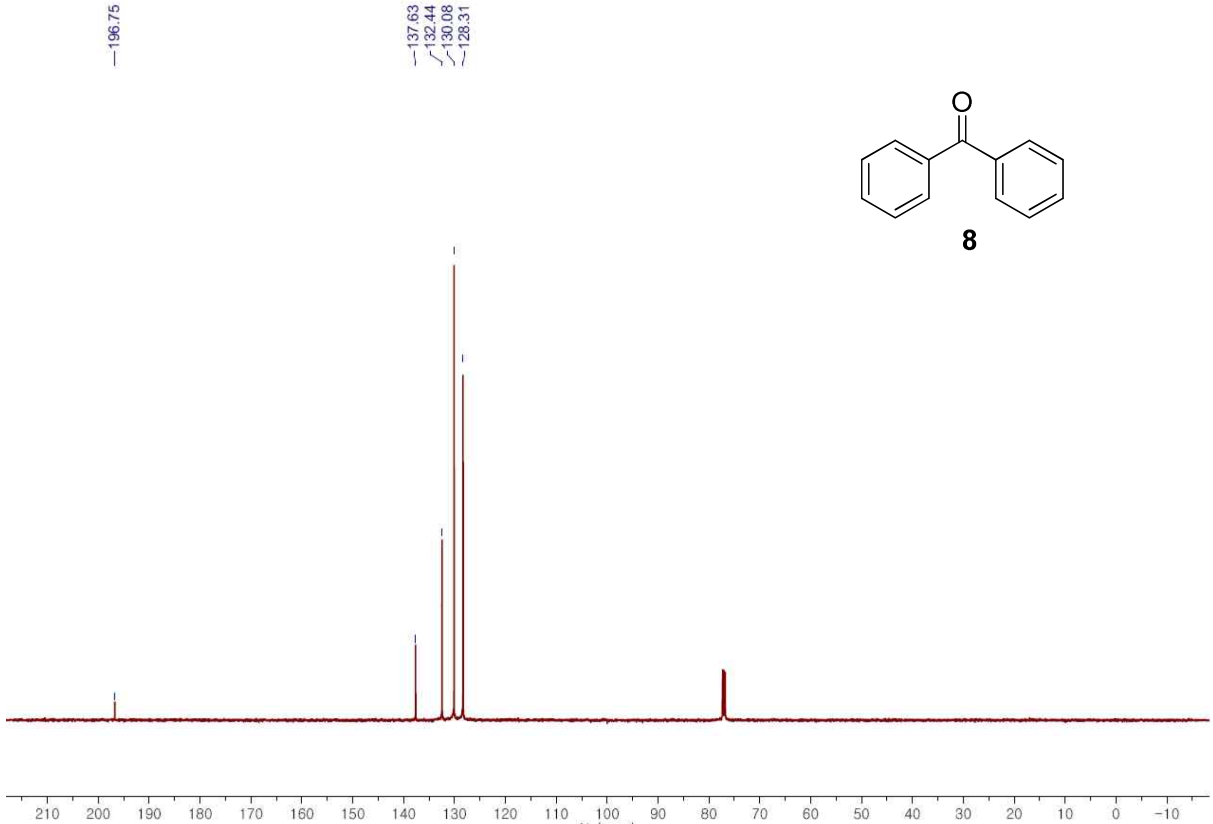
196.75
137.63
132.44
130.08
128.31
O
8
210 200 190 180 170 160 150 140 130 120 110 100 90 80 70 60 50 40 30 20 10 0 -10
f1 (ppm)

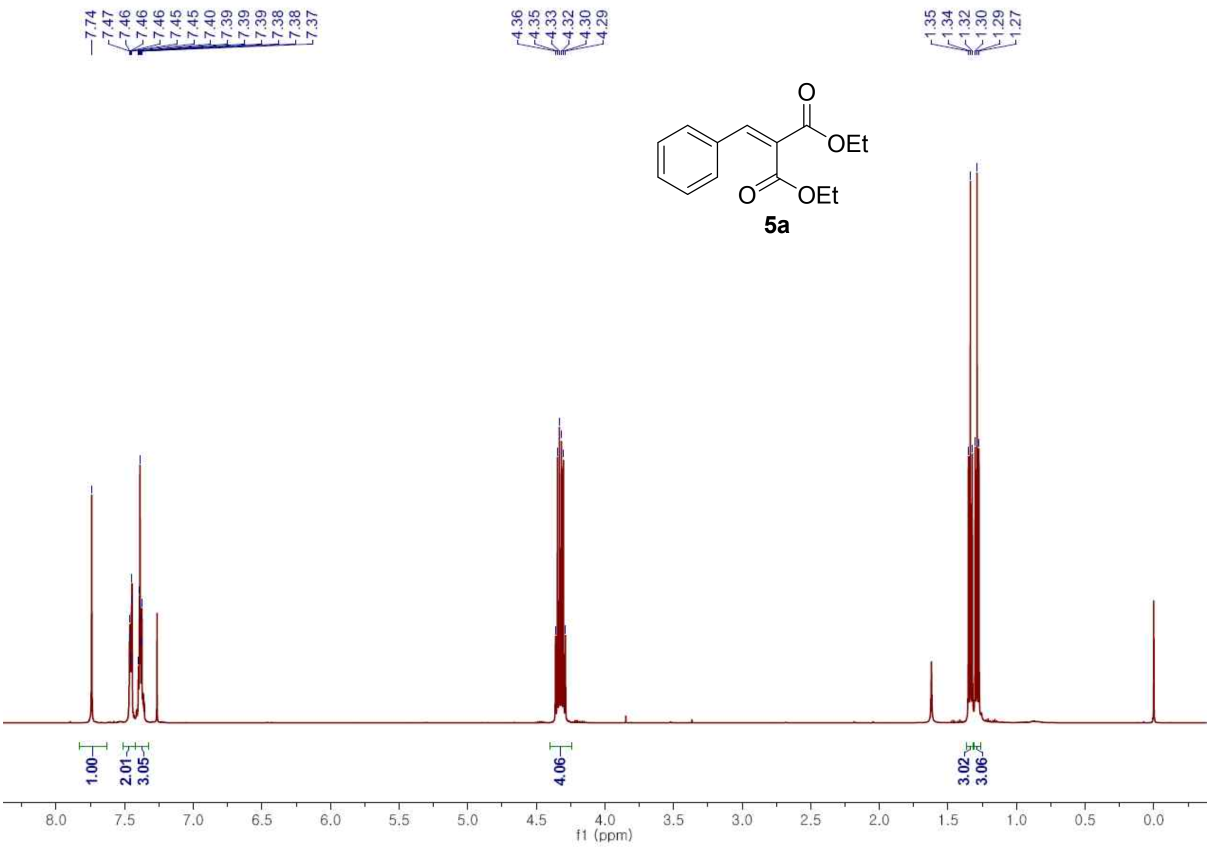
7.74
7.47
7.46
7.46
7.46
7.45
7.45
7.40
7.39
7.39
7.39
7.38
7.38
7.37
4.36
4.35
4.33
4.32
4.30
4.29
1.35
1.34
1.32
1.30
1.29
1.27
O
OEt
O
OEt
5a
1.00
2.01
3.05
4.06
3.02
3.06
8.0 7.5 7.0 6.5 6.0 5.5 5.0 4.5 4.0 3.5 3.0 2.5 2.0 1.5 1.0 0.5 0.0
f1 (ppm)

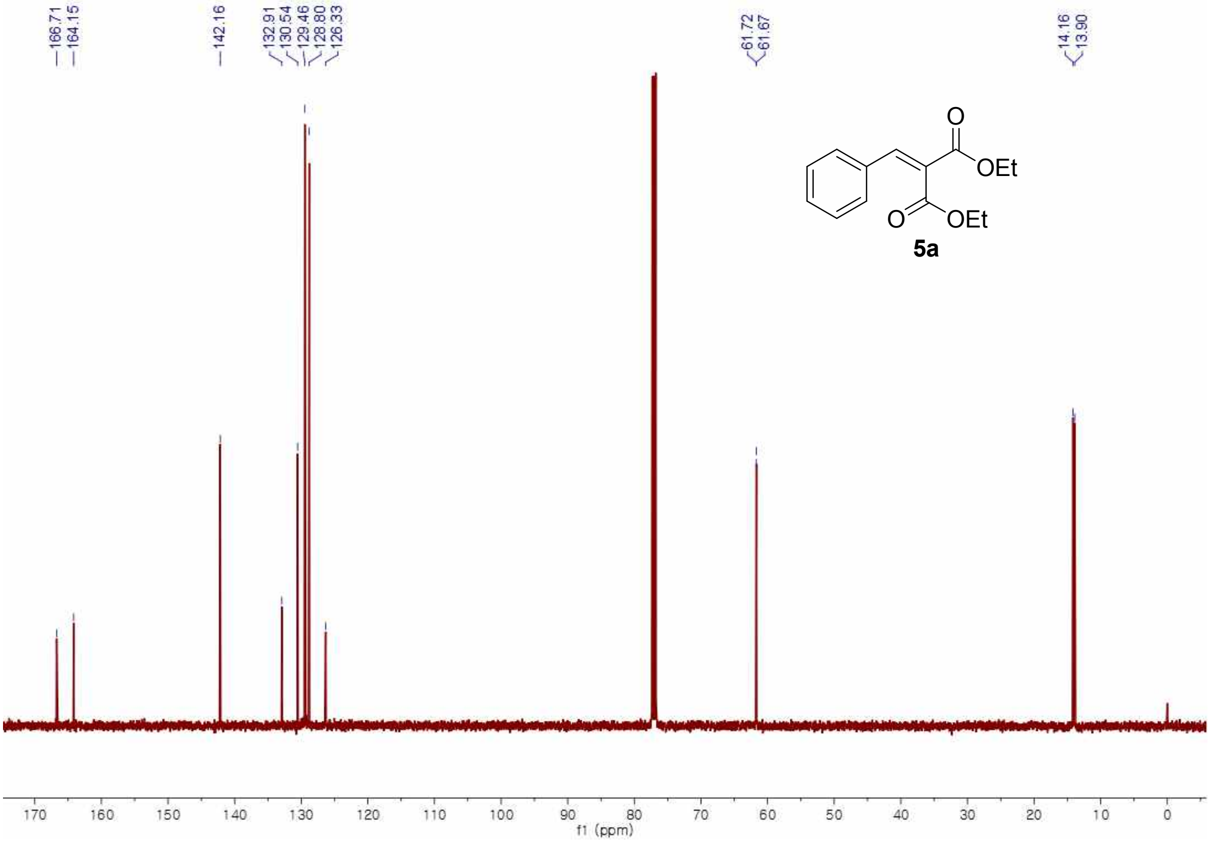
166.71
164.15
142.16
132.91
130.54
129.46
128.80
126.33
61.72
61.67
14.16
13.90
O
OEt
O
OEt
5a
170 160 150 140 130 120 110 100 90 80 70 60 50 40 30 20 10 0
f1 (ppm)

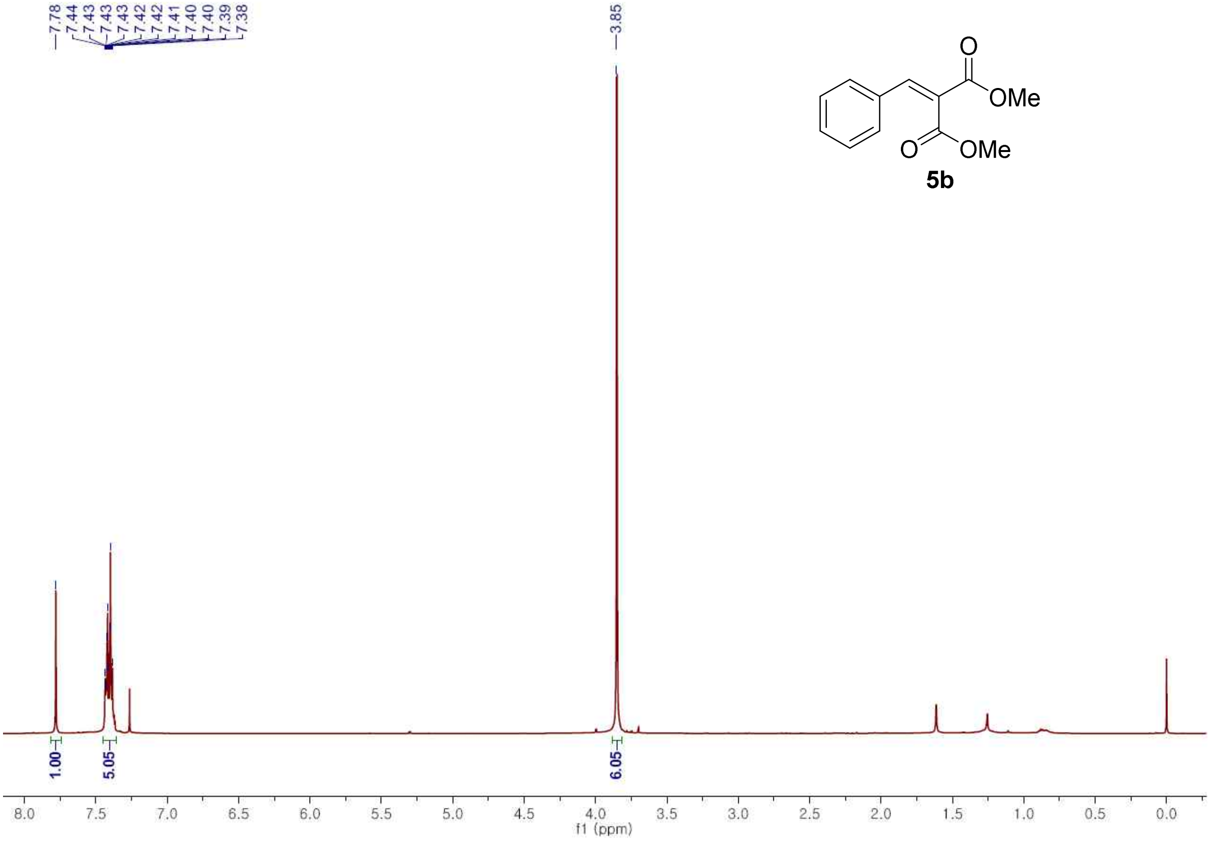

7.78
7.44
7.43
7.43
7.43
7.42
7.42
7.41
7.40
7.40
7.39
7.38
3.85
O
OMe
O
OMe
5b
1.00
5.05
6.05
8.0 7.5 7.0 6.5 6.0 5.5 5.0 4.5 4.0 3.5 3.0 2.5 2.0 1.5 1.0 0.5 0.0
f1 (ppm)

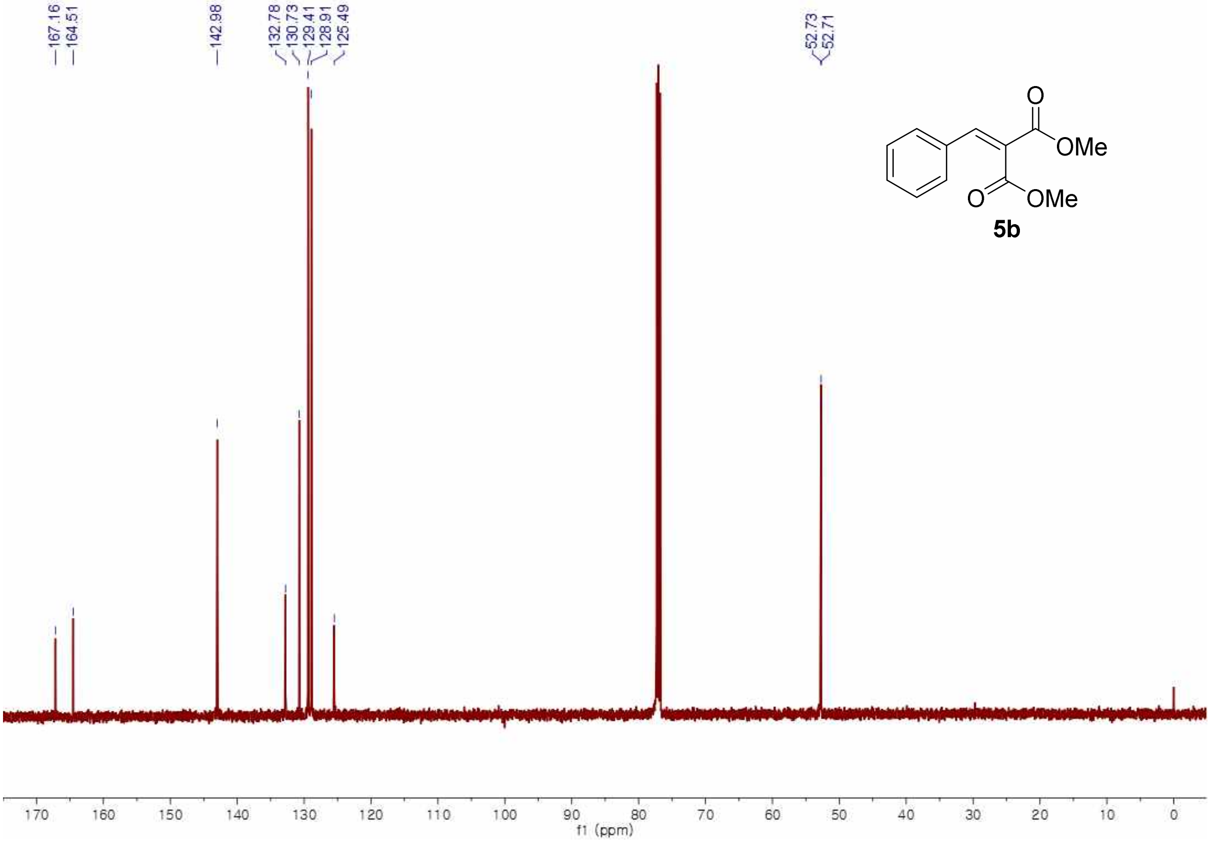

167.16
164.51
142.98
132.78
130.73
129.41
128.91
125.49
52.73
52.71
O
OMe
O
OMe
5b
170 160 150 140 130 120 110 100 90 80 70 60 50 40 30 20 10 0
f1 (ppm)

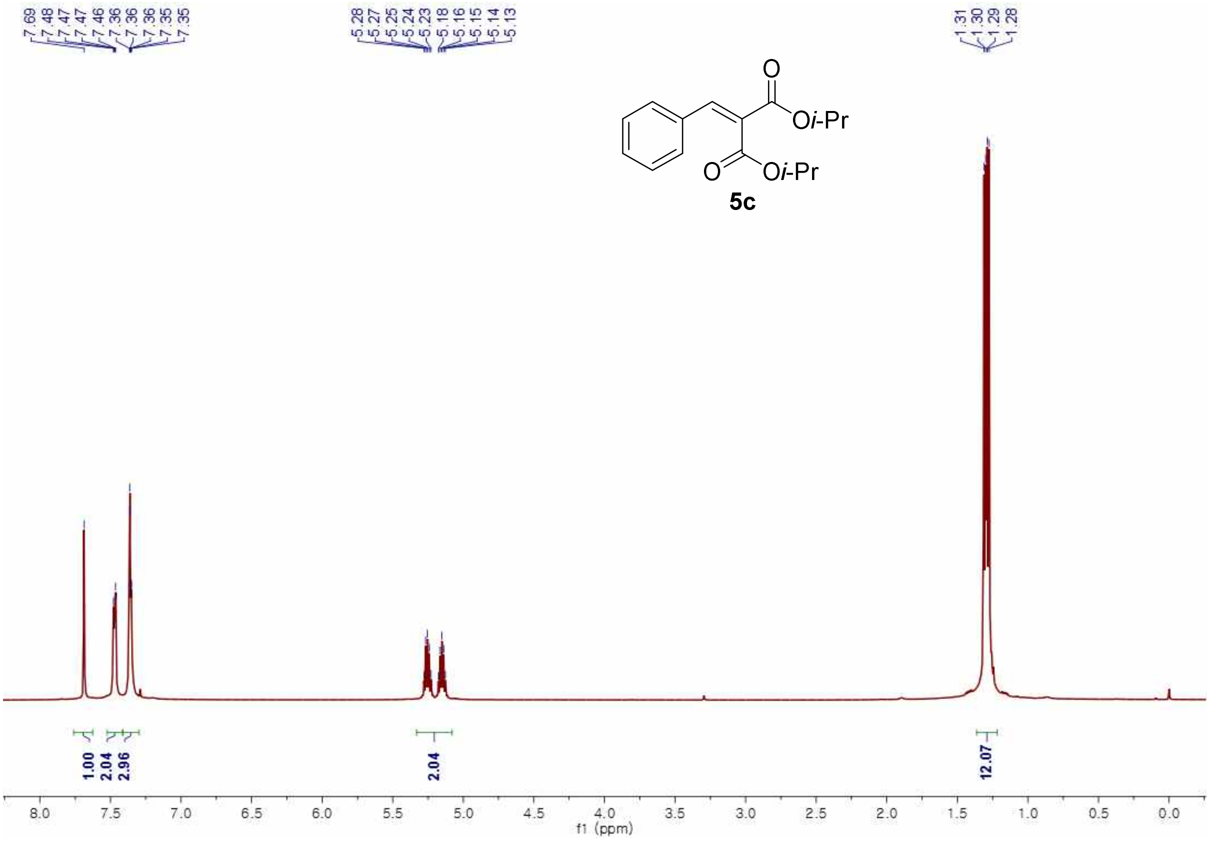
7.69
7.48
7.47
7.47
7.46
7.36
7.36
7.36
7.35
7.35
5.28
5.27
5.25
5.24
5.23
5.18
5.16
5.15
5.14
5.13
1.31
1.30
1.29
1.28
O
O*i*-Pr
O
O*i*-Pr
5c
1.00
2.04
2.96
2.04
12.07
f1 (ppm)

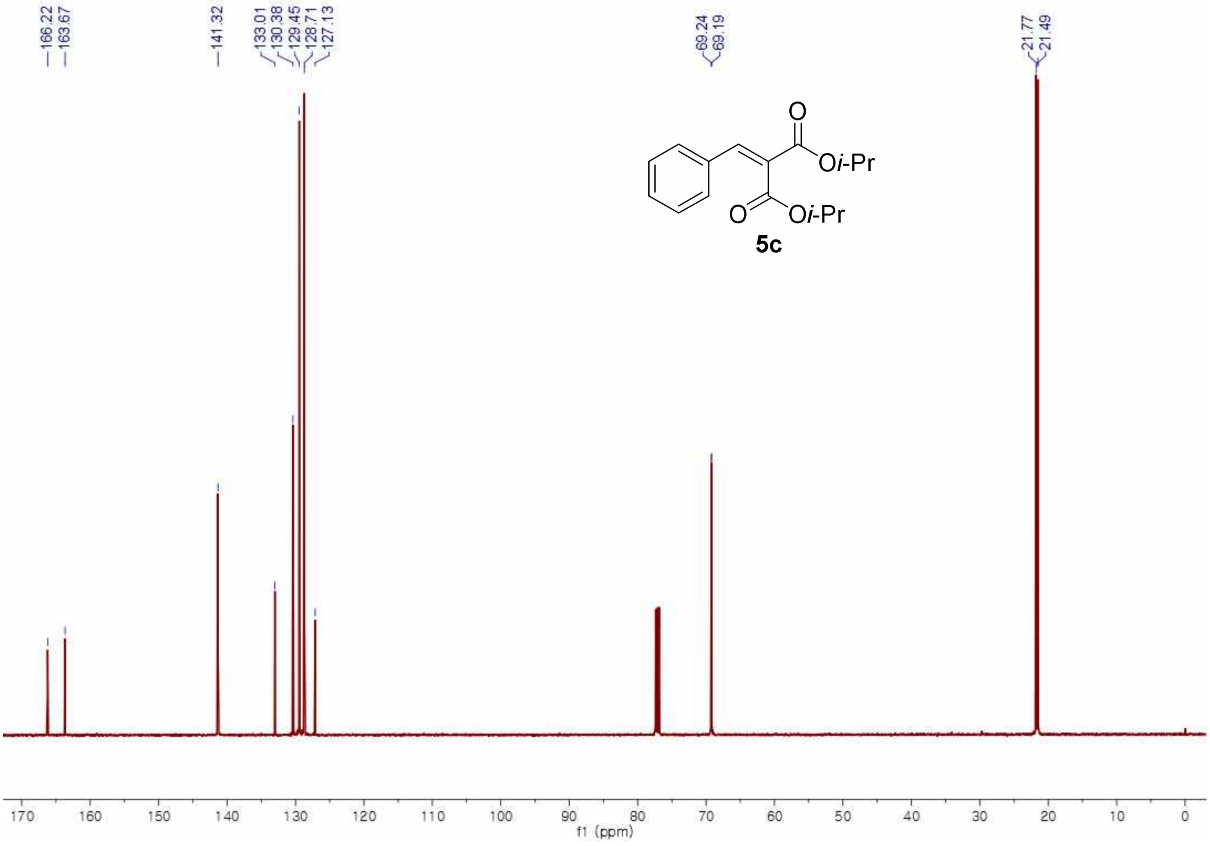
166.22
163.67
141.32
133.01
130.38
129.45
128.71
127.13
69.24
69.19
21.77
21.49
O
O*i*-Pr
O
O*i*-Pr
5c
f1 (ppm)

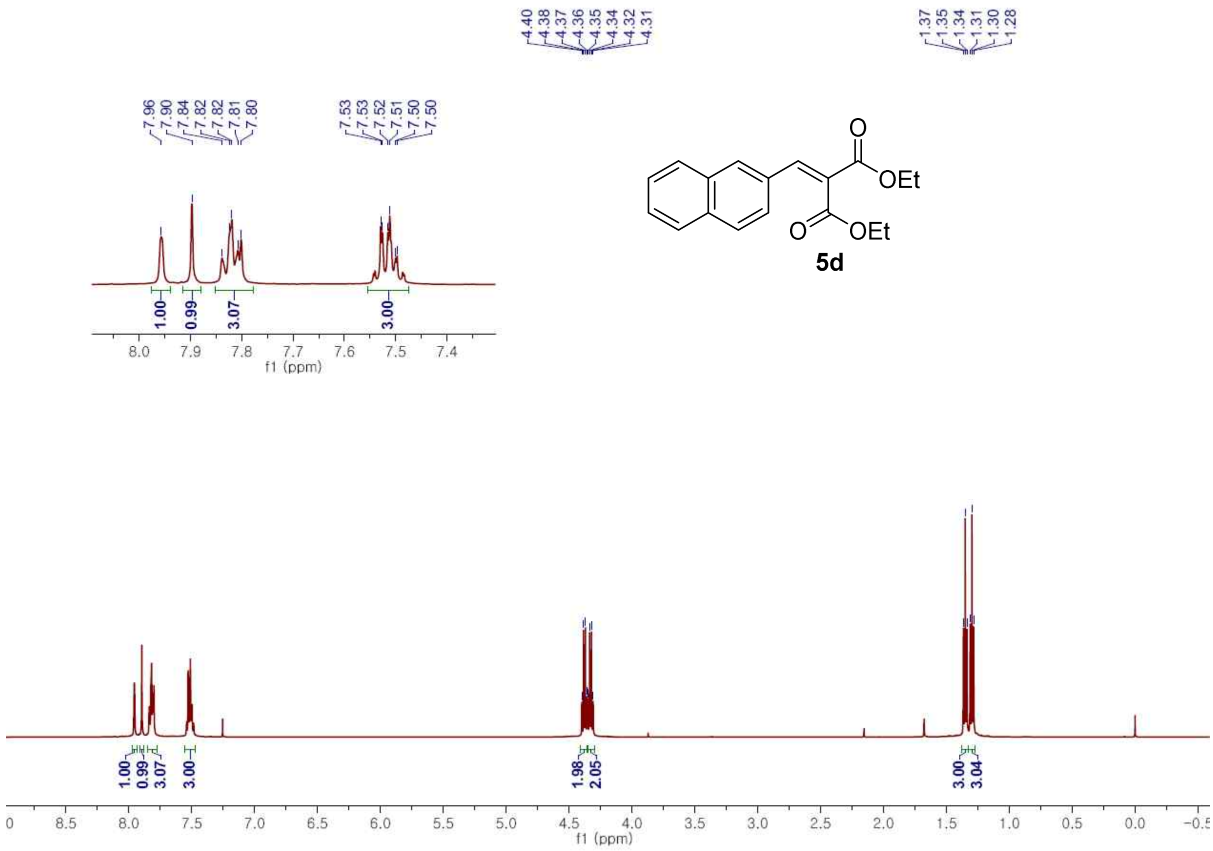

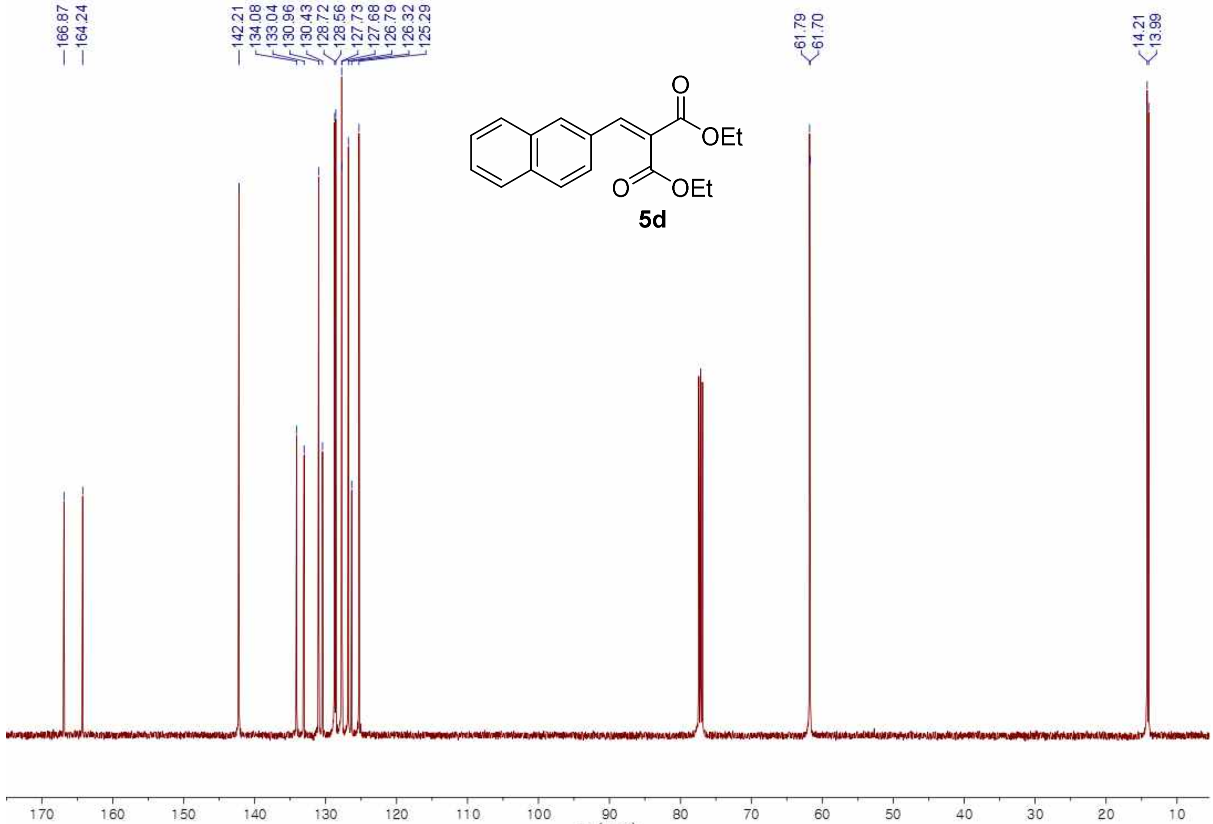

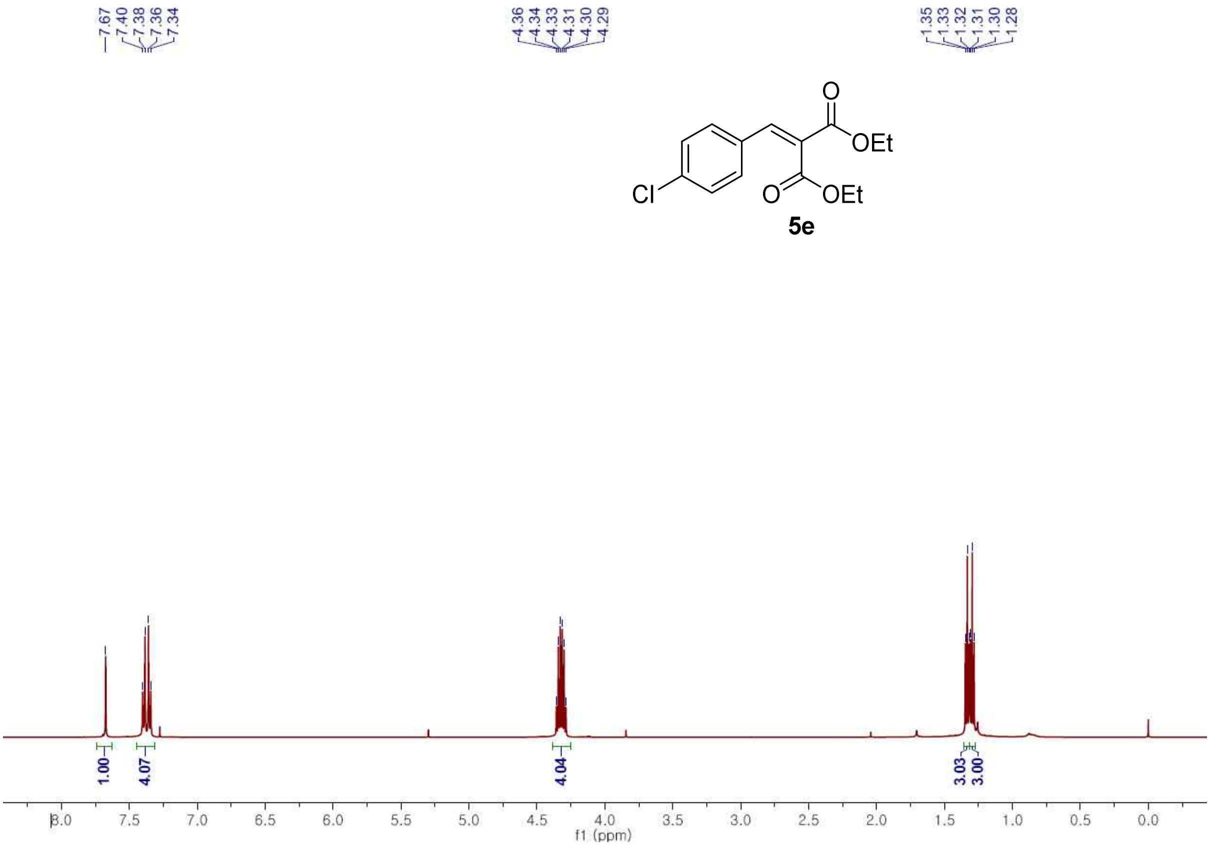
7.67
7.40
7.38
7.36
7.34
4.36
4.34
4.33
4.31
4.30
4.29
1.35
1.33
1.32
1.31
1.30
1.28
O
OEt
Cl
O
OEt
5e
1.00
4.07
4.04
3.03
3.00
f1 (ppm)

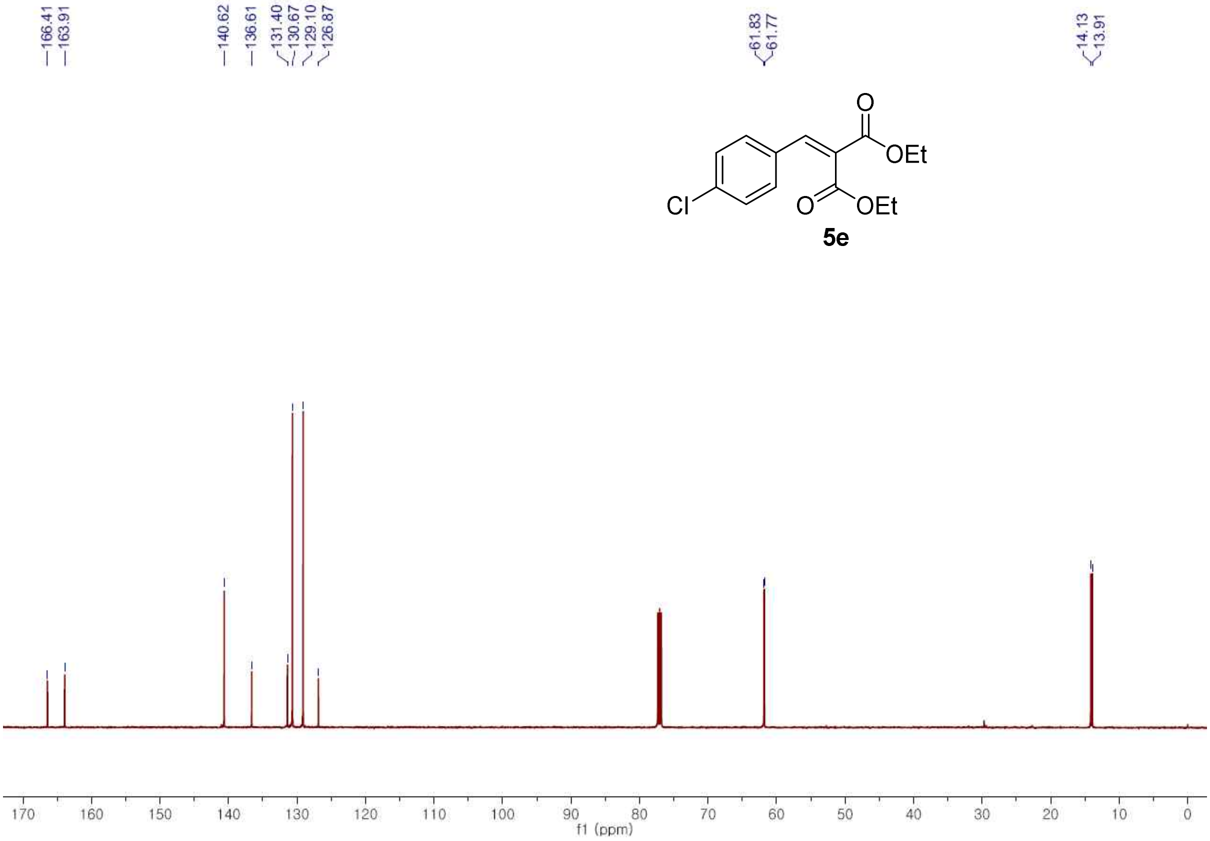
166.41
163.91
140.62
136.61
131.40
130.67
129.10
126.87
61.83
61.77
14.13
13.91
O
OEt
Cl
O
OEt
5e
f1 (ppm)

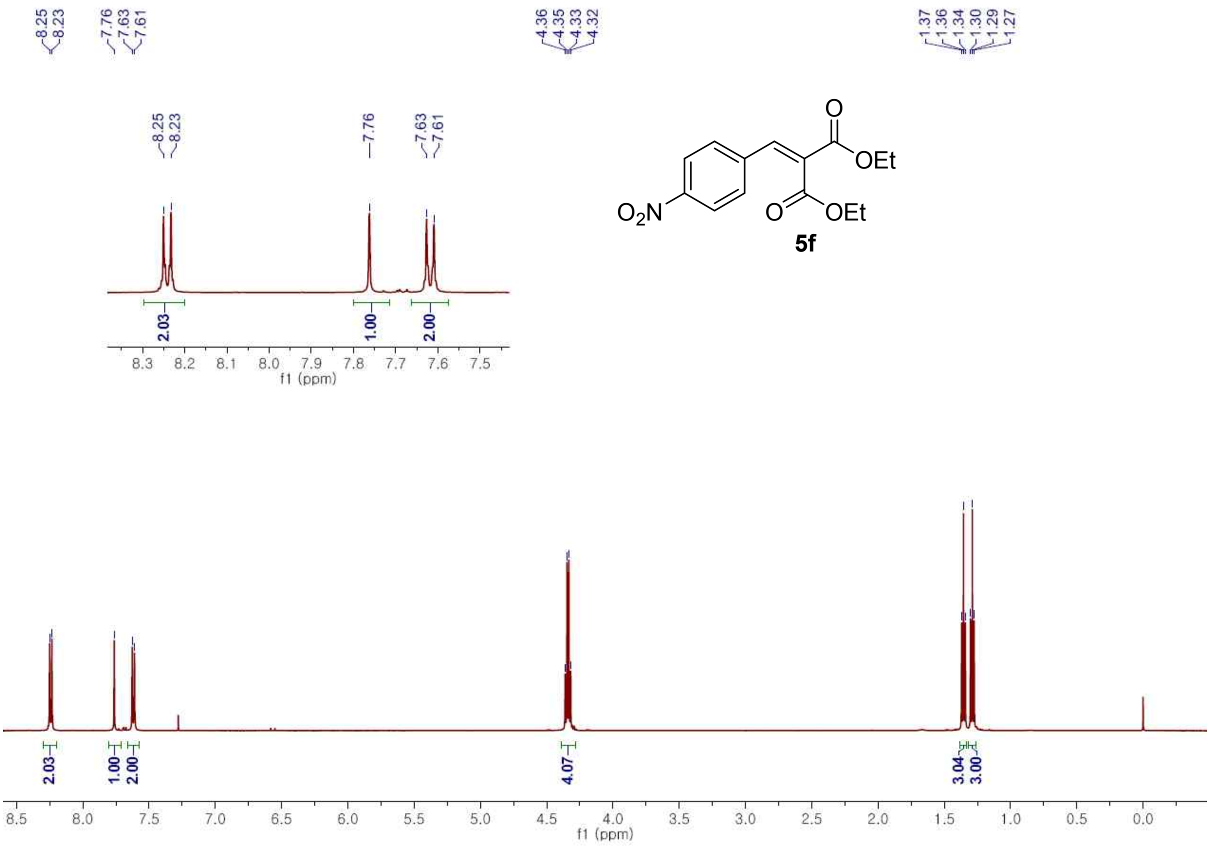
8.25
8.23
7.76
7.63
7.61
4.36
4.35
4.33
4.32
1.37
1.36
1.34
1.30
1.29
1.27
O
OEt
O₂N
O
OEt
2.03
1.00
2.00
4.07
3.04
3.00
f1 (ppm)

5f

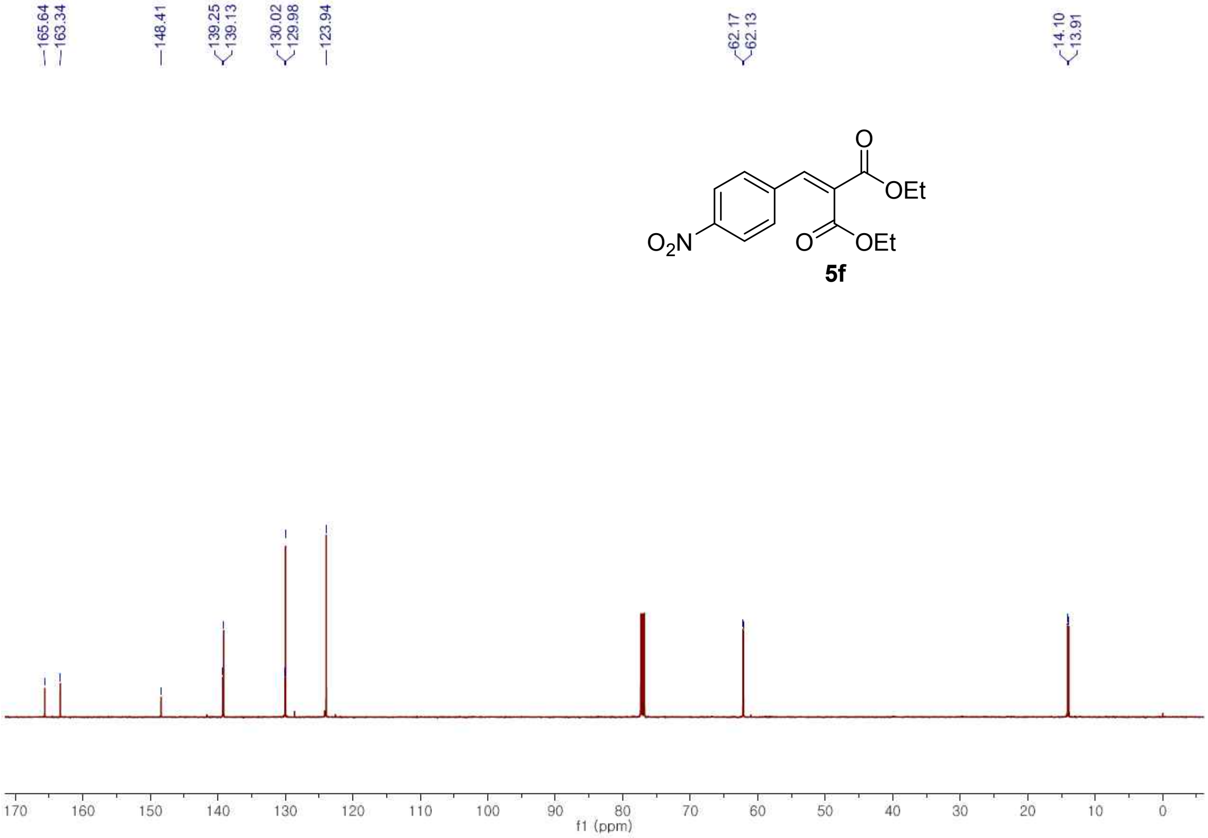
165.64
163.34
148.41
139.25
139.13
130.02
129.98
123.94
62.17
62.13
14.10
13.91
O
OEt
O₂N
O
OEt
f1 (ppm)

5f

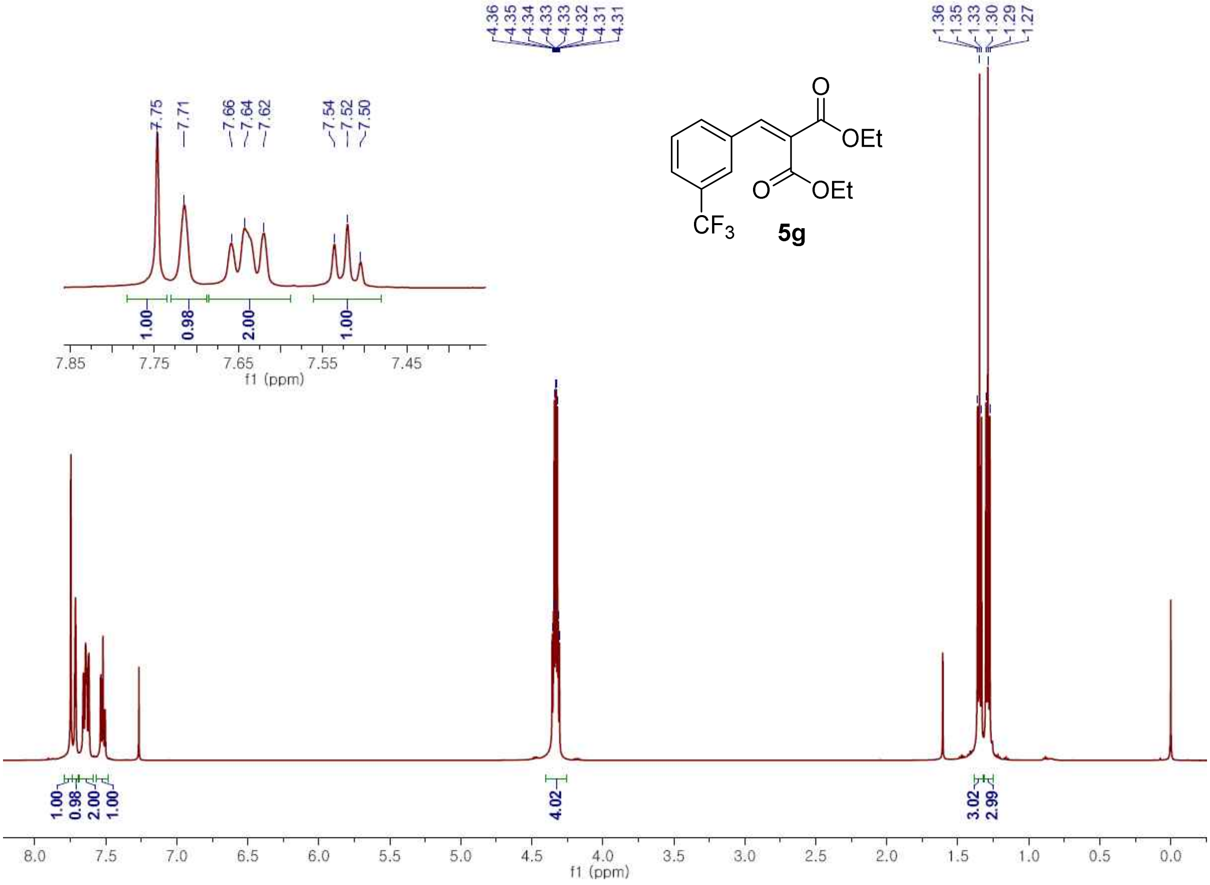
4.36 4.35 4.34 4.33 4.33 4.32 4.31 4.31
1.36 1.35 1.33 1.30 1.29 1.27
7.75 7.71 7.66 7.64 7.62 7.54 7.52 7.50
1.00 0.98 2.00 1.00
O
OEt
O
OEt
CF3
5g
4.02
3.02 2.99
f1 (ppm)

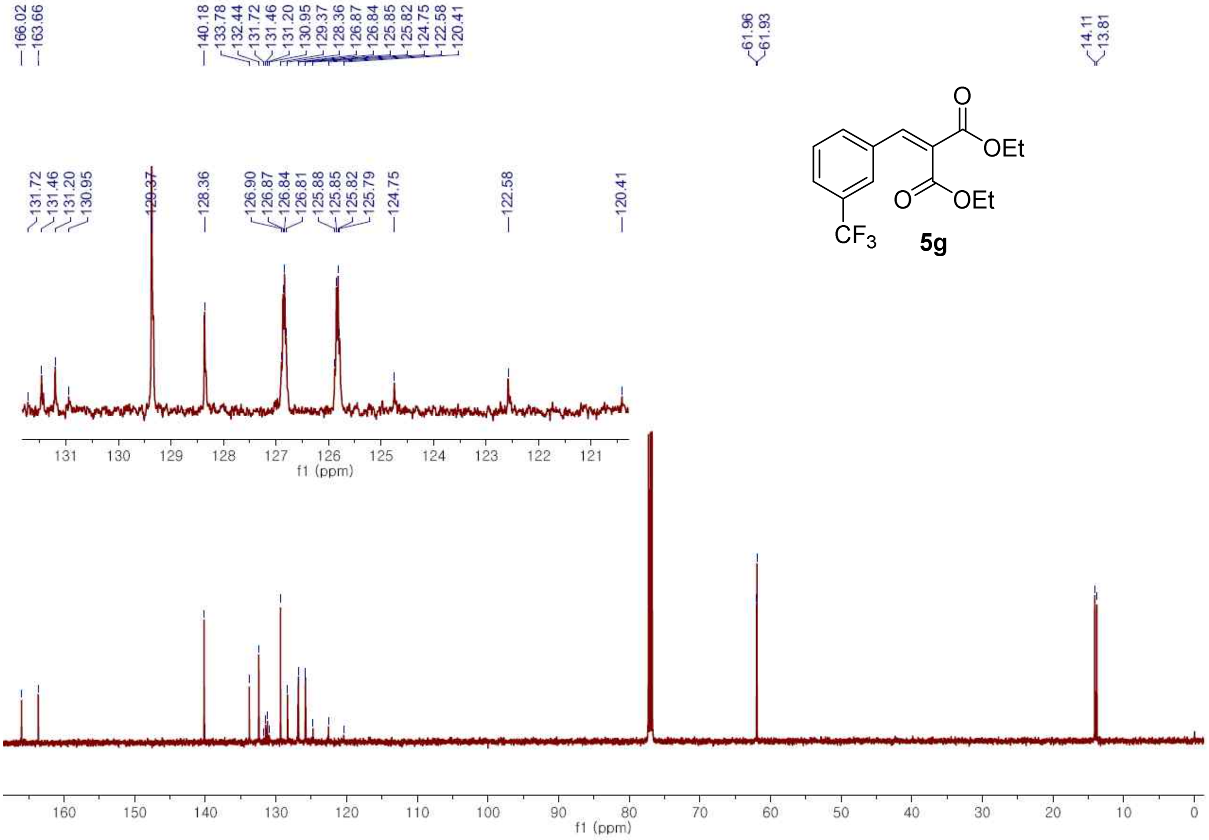
166.02 163.66
140.18 133.78 132.44 131.72 131.46 131.20 130.95 129.37 128.36 126.87 126.84 125.85 125.82 124.75 122.58 120.41
61.96 61.93
14.11 13.81
131.72 131.46 131.20 130.95
129.37
128.36
126.90 126.87 126.84 126.81 125.88 125.85 125.82 125.79
124.75
122.58
120.41
O
OEt
O
OEt
CF3
5g
f1 (ppm)

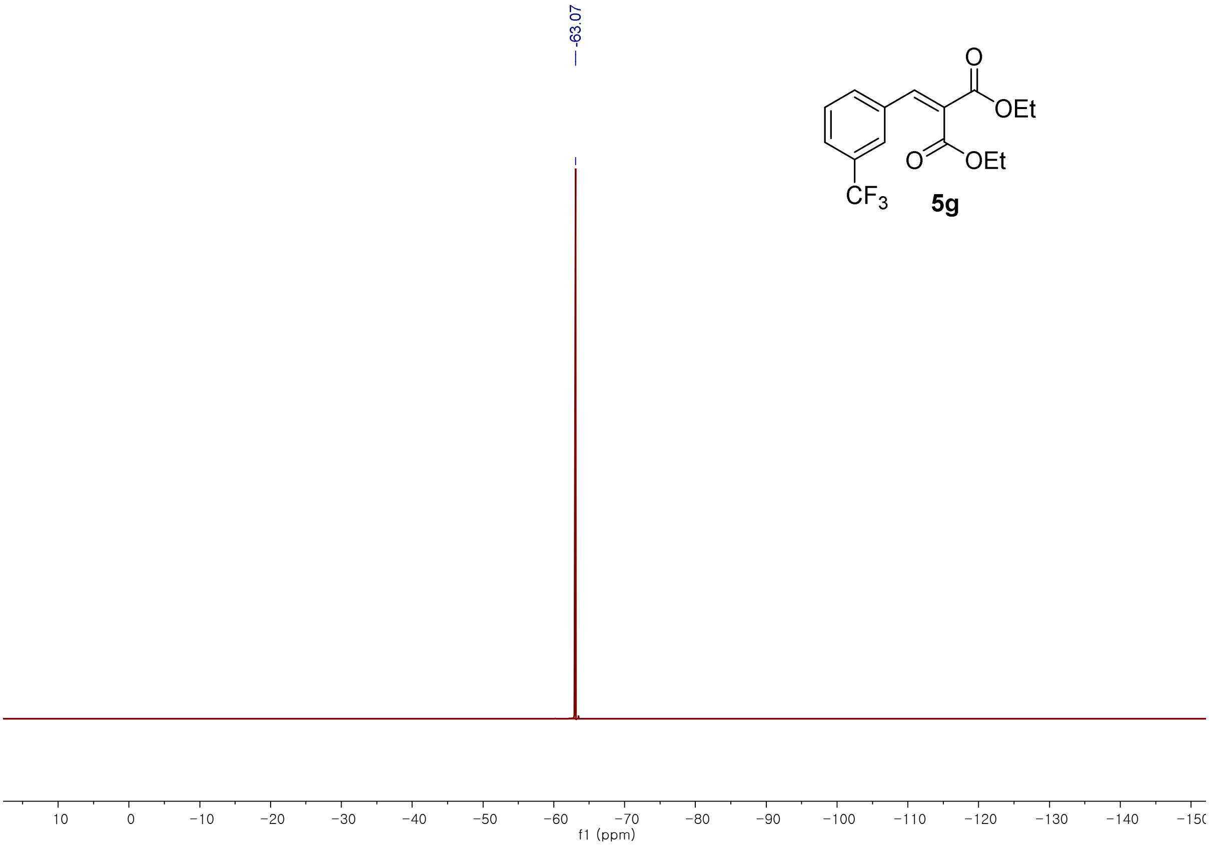
-63.07
O
OEt
O
OEt
CF3
5g
10
0
−10
−20
−30
−40
−50
−60
−70
−80
−90
−100
−110
−120
−130
−140
−150
f1 (ppm)

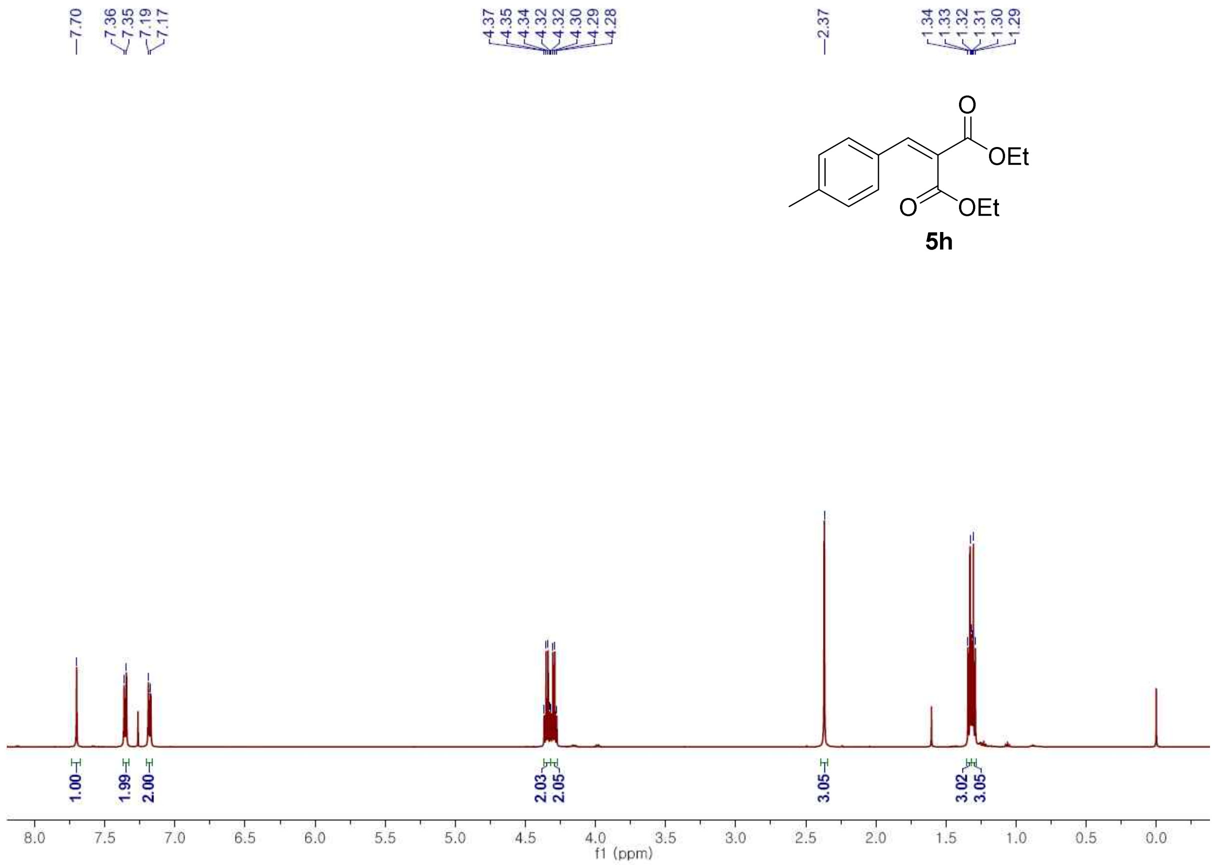
7.70
7.36
7.35
7.19
7.17
4.37
4.35
4.34
4.32
4.32
4.30
4.29
4.28
2.37
1.34
1.33
1.32
1.31
1.30
1.29
OEt
OEt
5h
1.00
1.99
2.00
2.03
2.05
3.05
3.02
3.05
f1 (ppm)

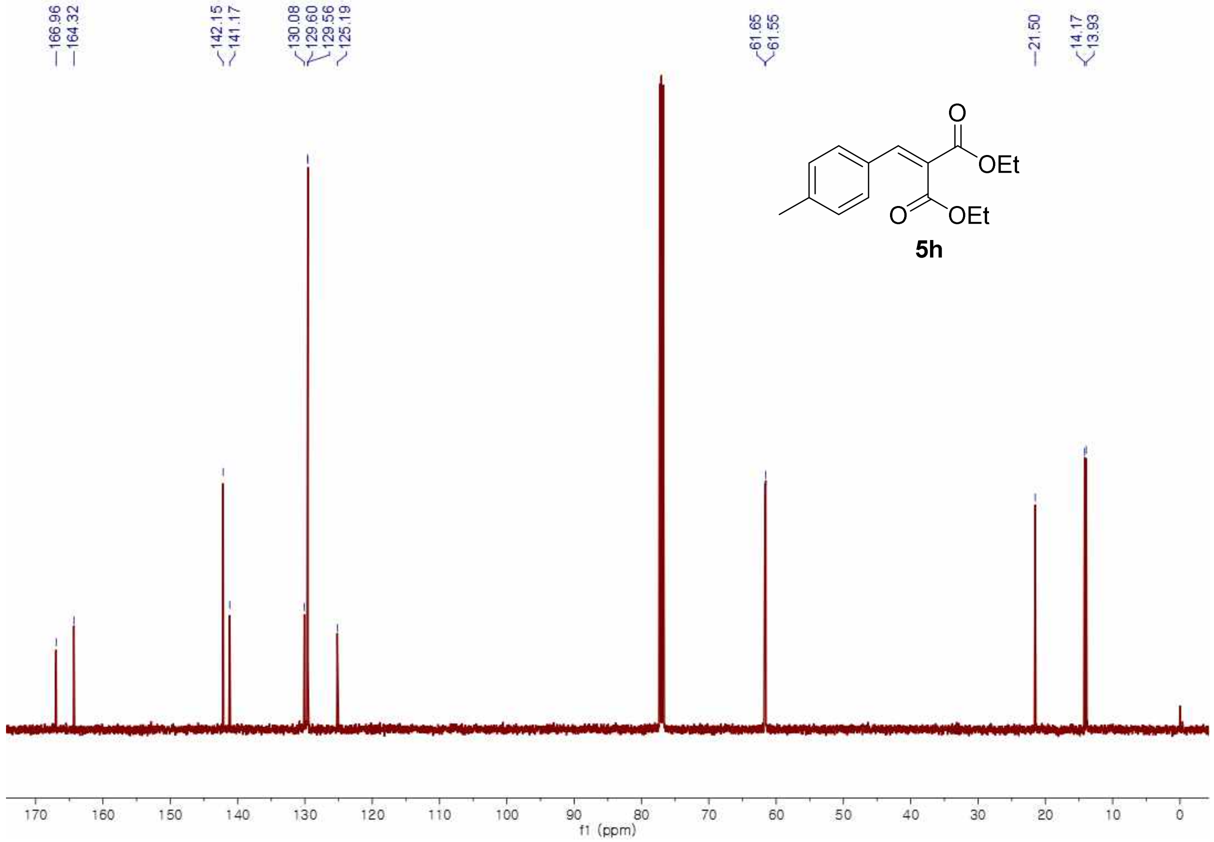
166.96
164.32
142.15
141.17
130.08
129.60
129.56
125.19
61.65
61.55
21.50
14.17
13.93
OEt
OEt
5h
f1 (ppm)

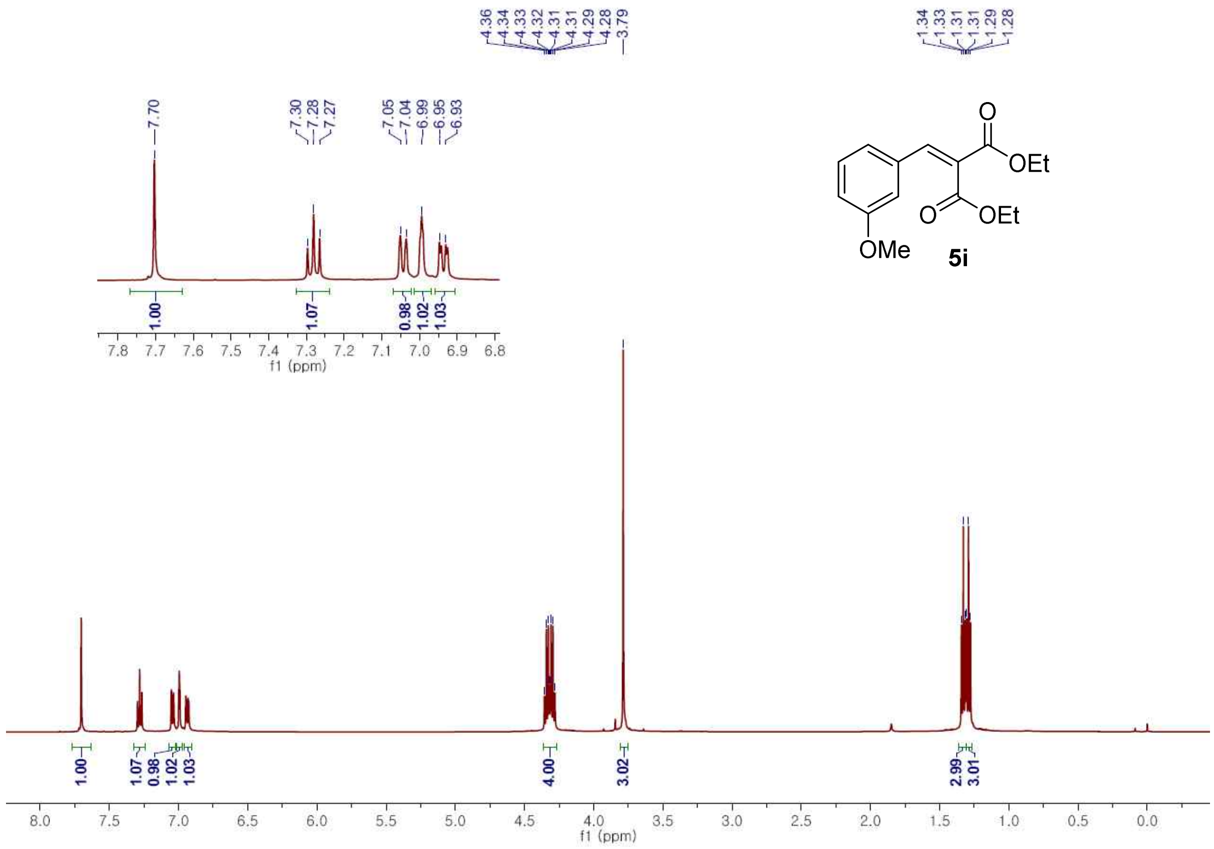
4.36 4.34 4.33 4.32 4.31 4.31 4.29 4.28 3.79
1.34 1.33 1.31 1.31 1.29 1.28
7.70
7.30 7.28 7.27
7.05 7.04 6.99 6.95 6.93
1.00 1.07 0.98 1.02 1.03
7.8 7.7 7.6 7.5 7.4 7.3 7.2 7.1 7.0 6.9 6.8
f1 (ppm)
O
OEt
O
OEt
OMe
5i
1.00 1.07 0.98 1.02 1.03 4.00 3.02 2.99 3.01
8.0 7.5 7.0 6.5 6.0 5.5 5.0 4.5 4.0 3.5 3.0 2.5 2.0 1.5 1.0 0.5 0.0
f1 (ppm)

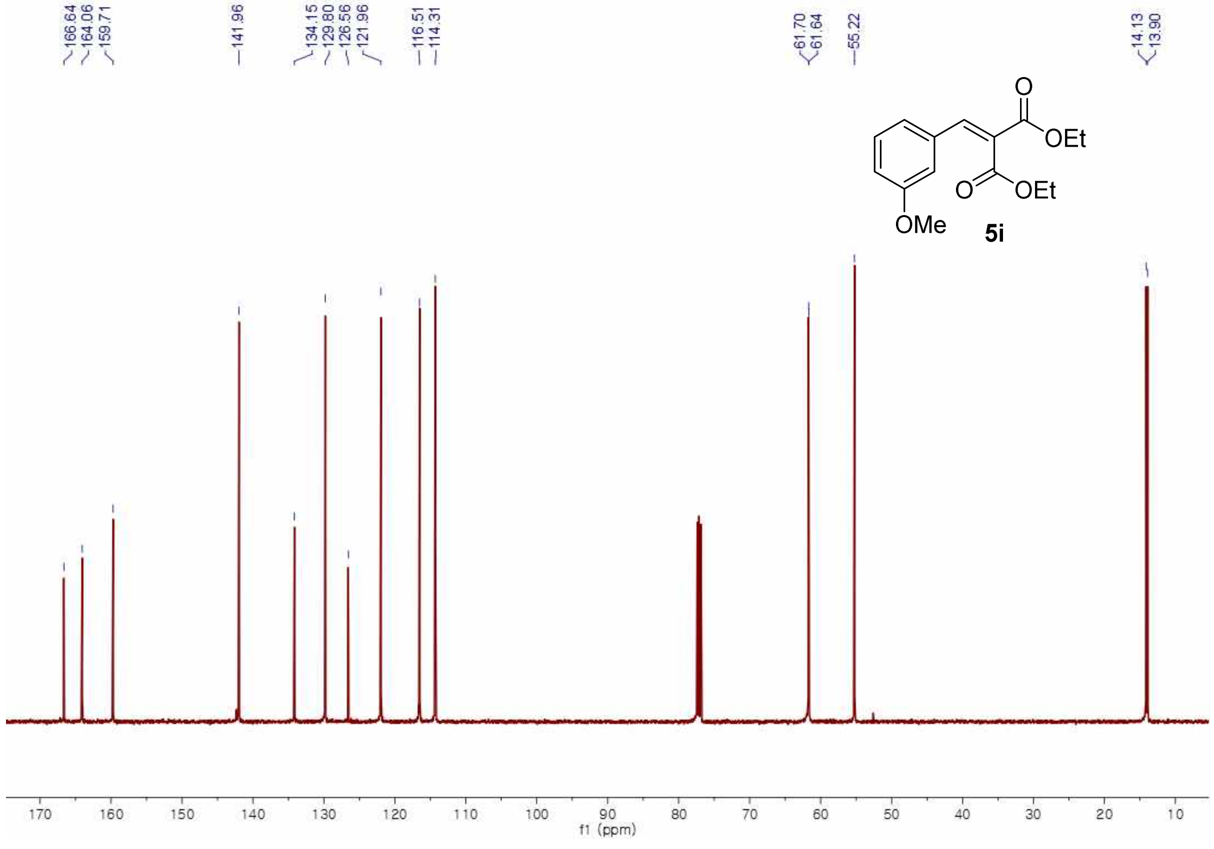
166.64 164.06 159.71
141.96
134.15 129.80 126.56 121.96
116.51 114.31
61.70 61.64
55.22
14.13 13.90
O
OEt
O
OEt
OMe
5i
170 160 150 140 130 120 110 100 90 80 70 60 50 40 30 20 10
f1 (ppm)

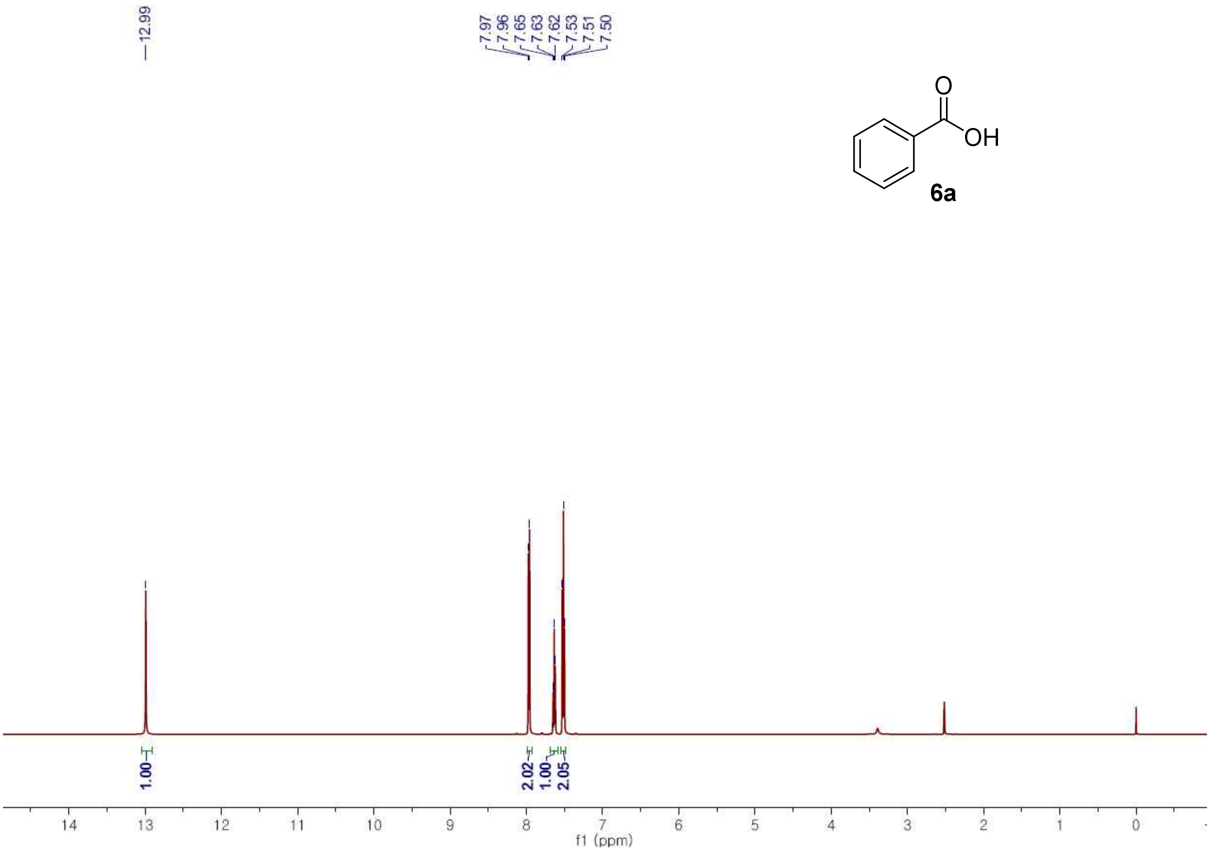
12.99
7.97
7.96
7.65
7.63
7.62
7.53
7.51
7.50
OH
O
6a
1.00
2.02
1.00
2.05
14
13
12
11
10
9
8
7
6
5
4
3
2
1
0
f1 (ppm)

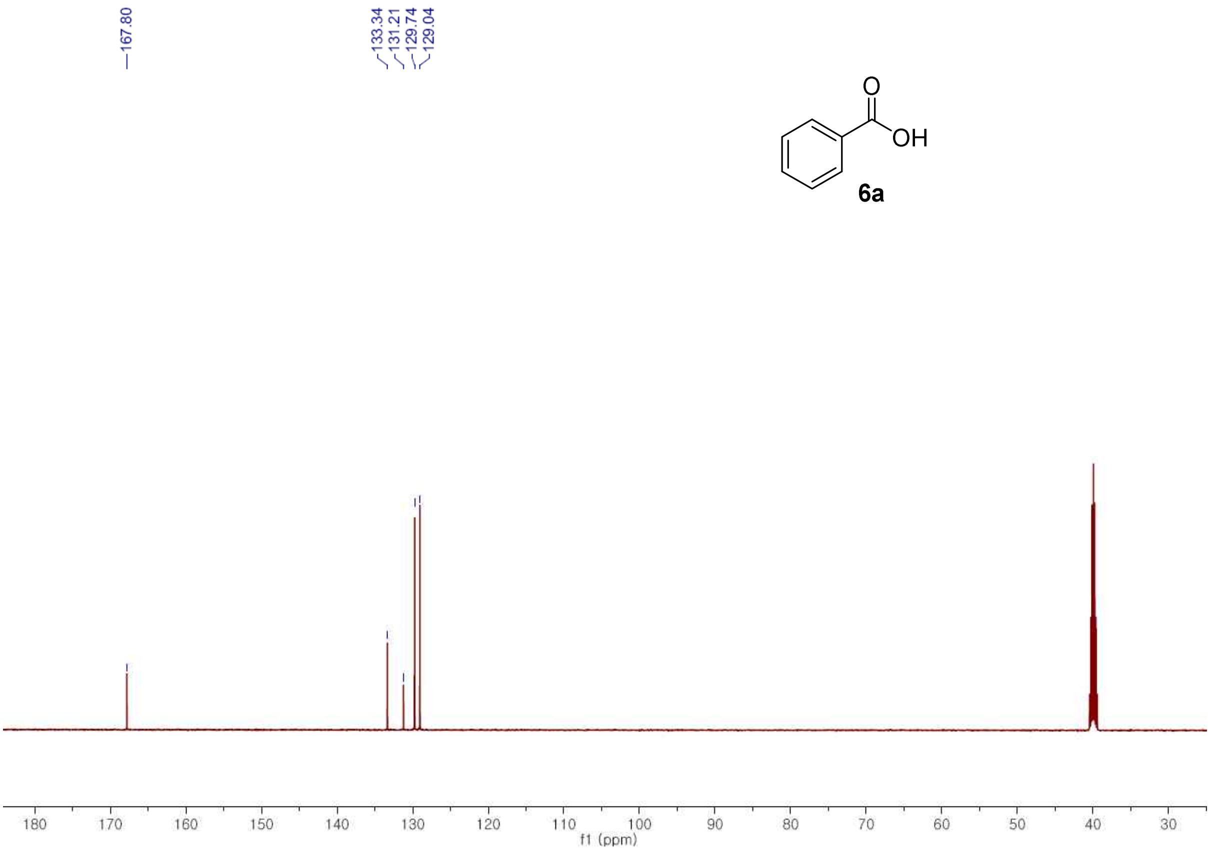
167.80
133.34
131.21
129.74
129.04
O
OH
6a
180
170
160
150
140
130
120
110
100
90
80
70
60
50
40
30
f1 (ppm)

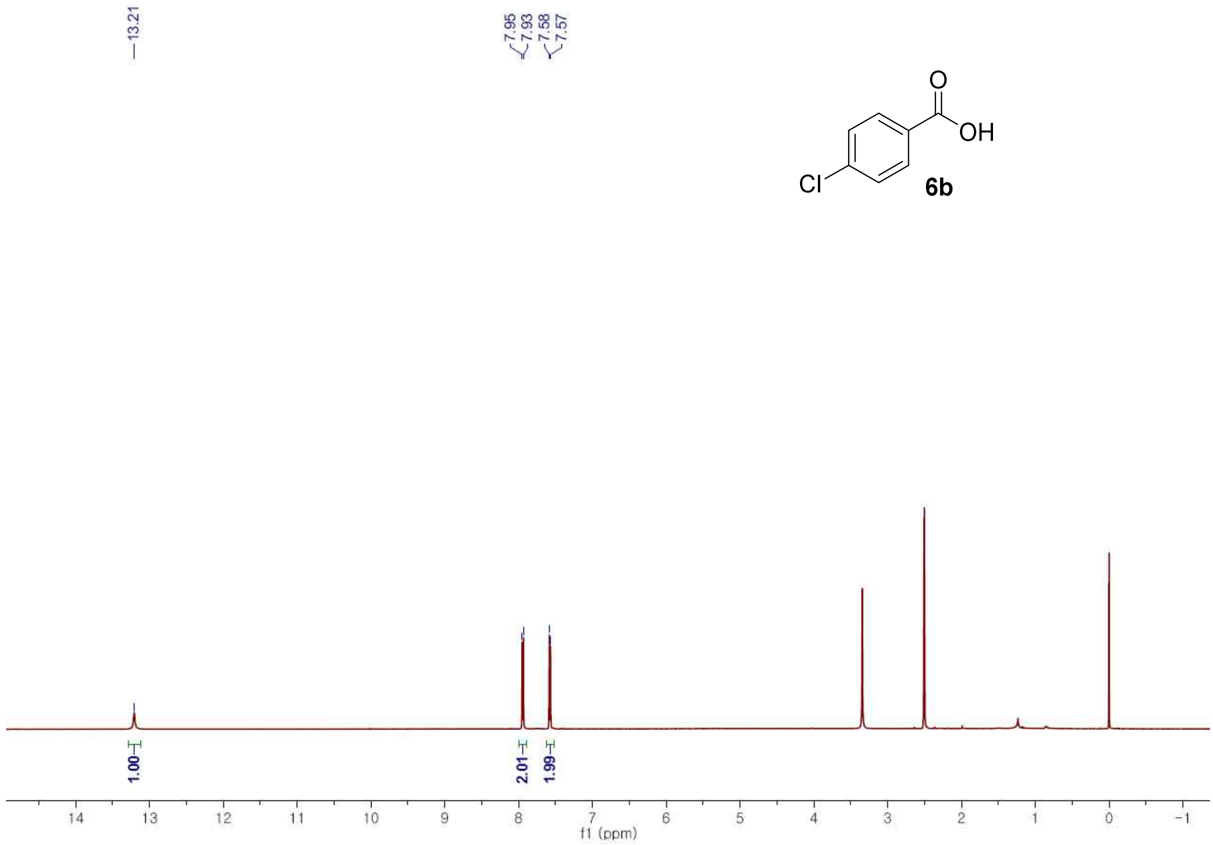
13.21
7.95
7.93
7.58
7.57
O
OH
Cl
6b
1.00
2.01
1.99
f1 (ppm)

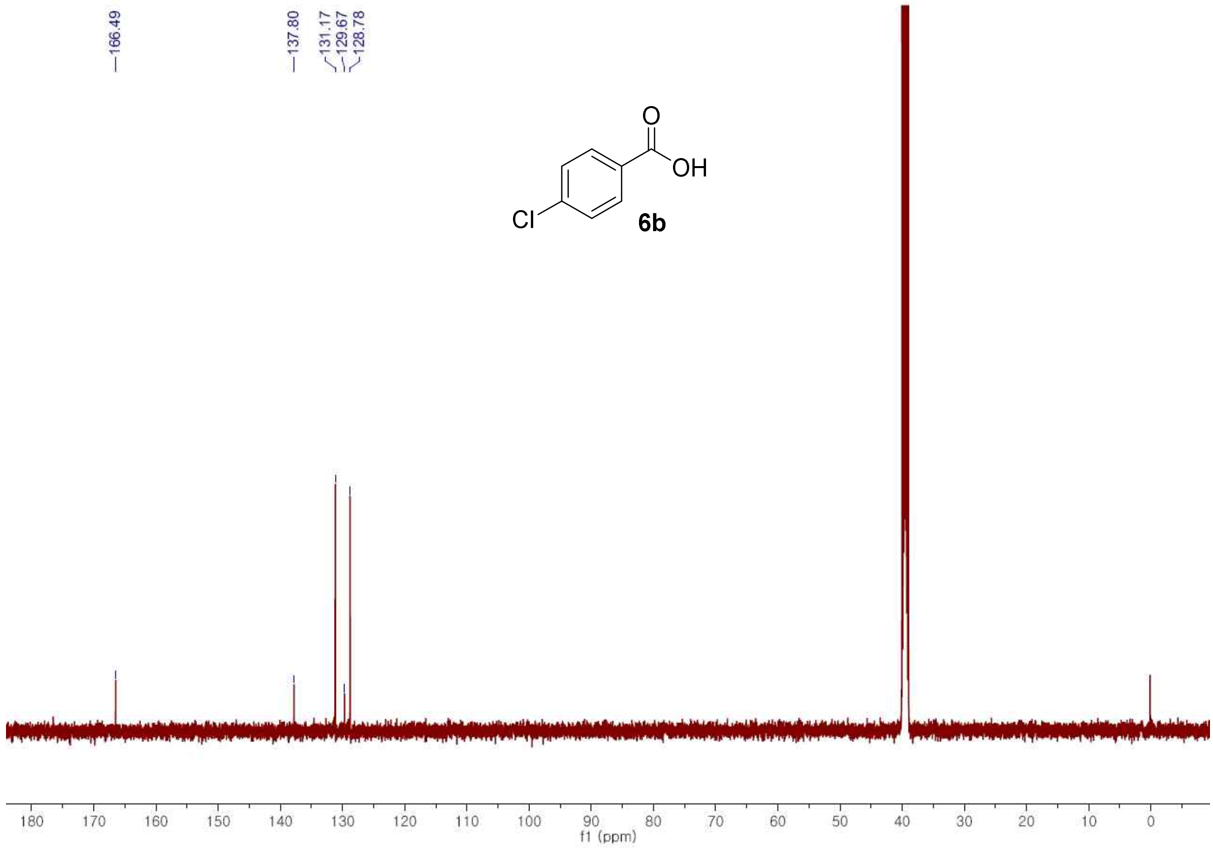
166.49
137.80
131.17
129.67
128.78
O
OH
Cl
6b
f1 (ppm)

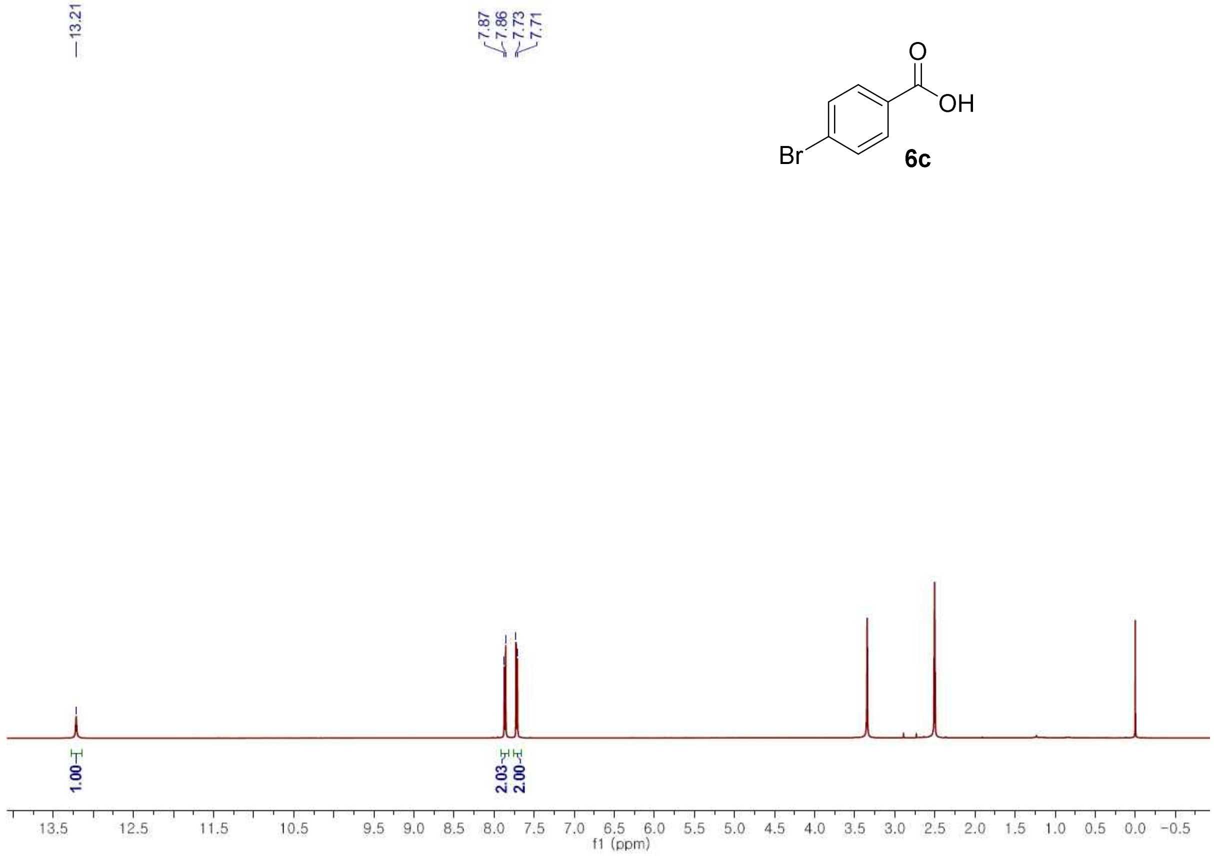

13.21
7.87
7.86
7.73
7.71
1.00
2.03
2.00
f1 (ppm)

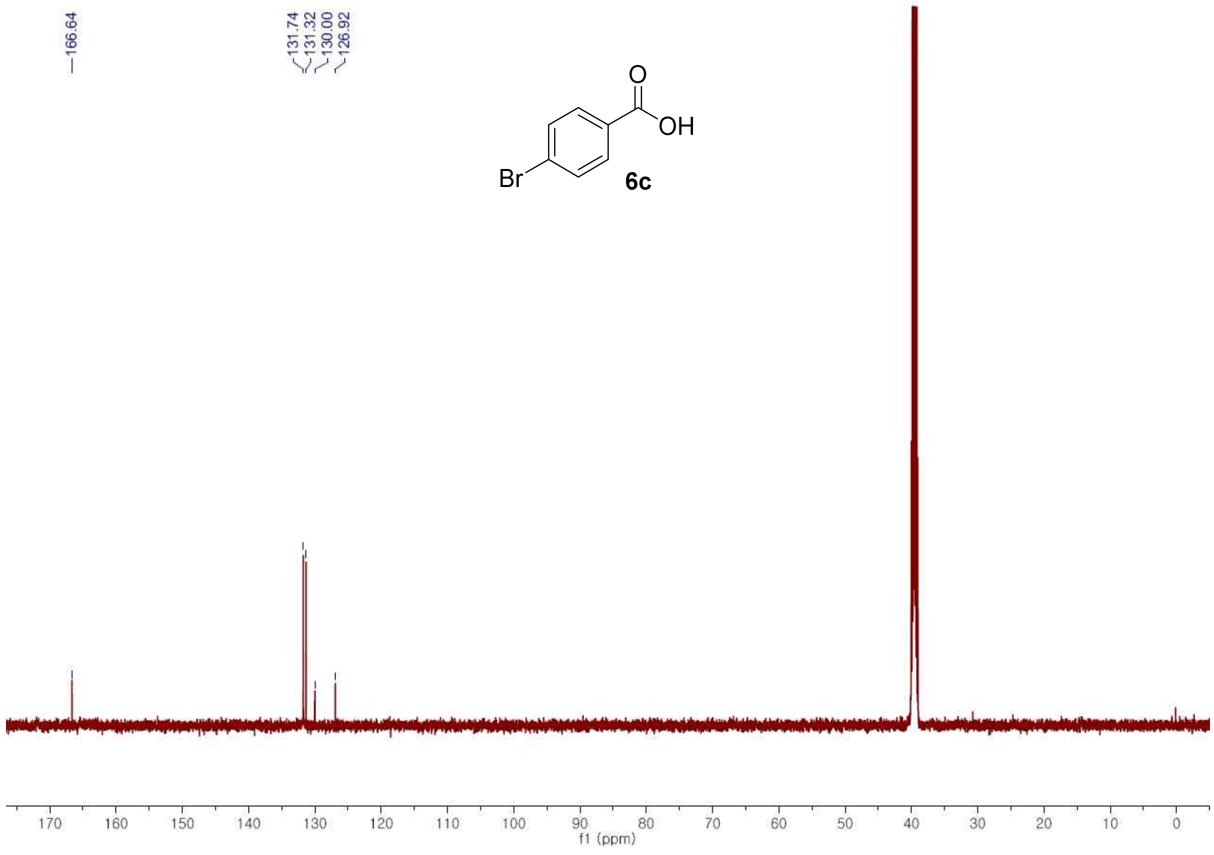

166.64
131.74
131.32
130.00
126.92
f1 (ppm)

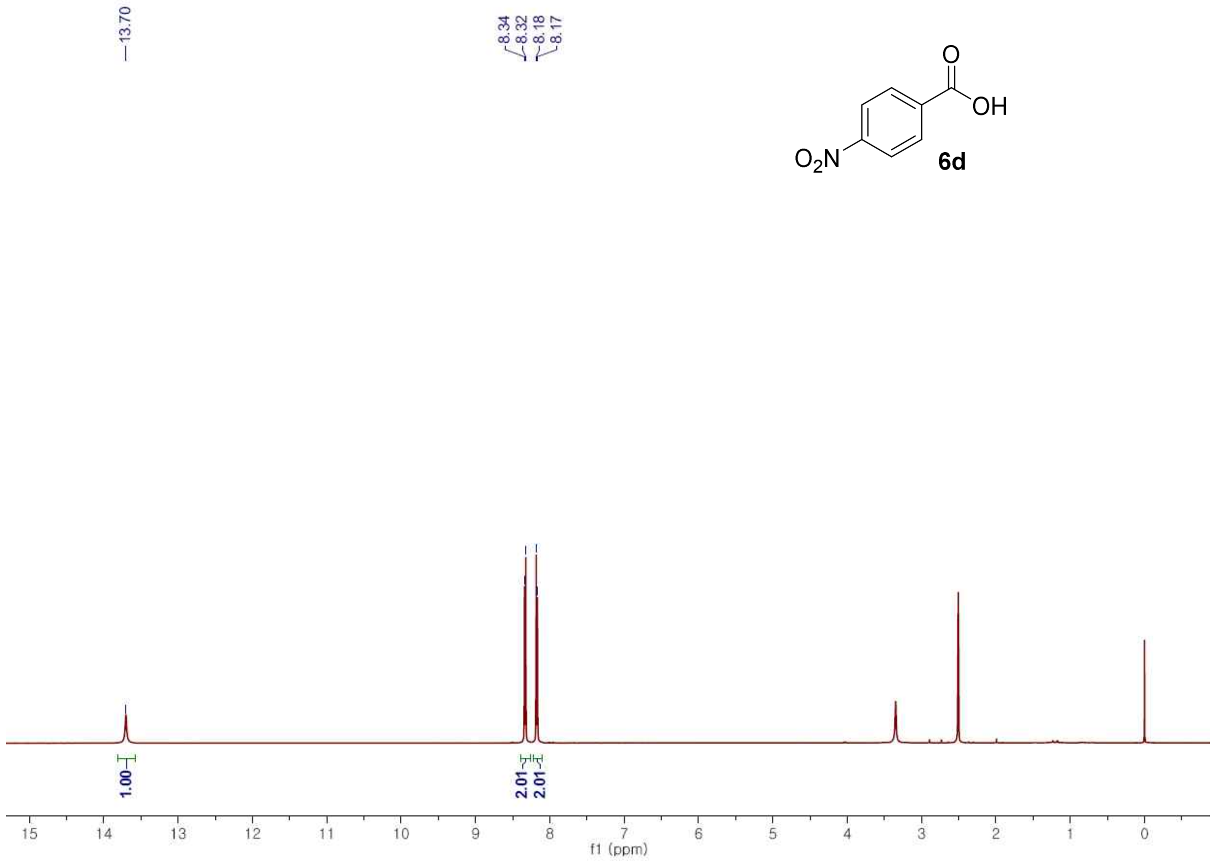
13.70
8.34
8.32
8.18
8.17
O
OH
O_2N
6d
1.00
2.01
2.01
f1 (ppm)

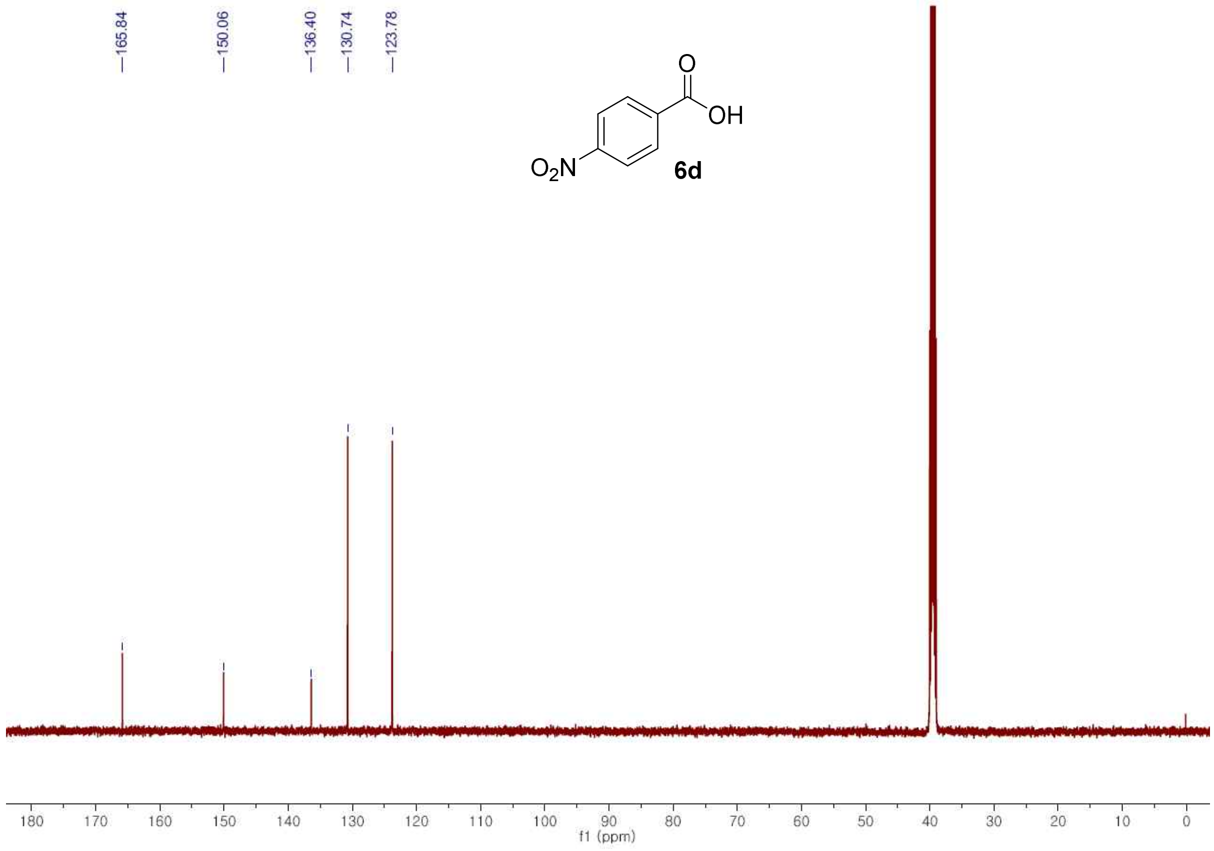
165.84
150.06
136.40
130.74
123.78
O
OH
O_2N
6d
f1 (ppm)

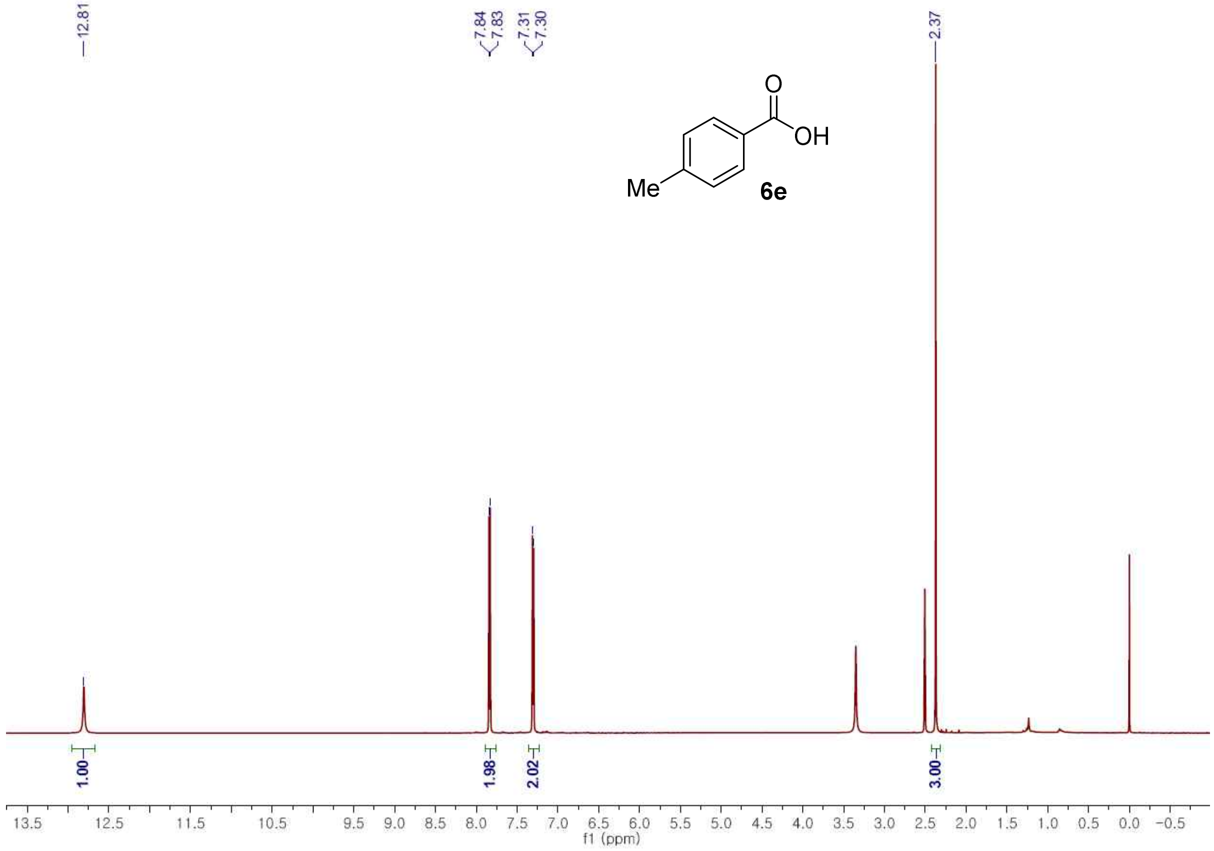
12.81
7.84
7.83
7.31
7.30
2.37
O
OH
Me
6e
1.00
1.98
2.02
3.00
13.5 12.5 11.5 10.5 9.5 9.0 8.5 8.0 7.5 7.0 6.5 6.0 5.5 5.0 4.5 4.0 3.5 3.0 2.5 2.0 1.5 1.0 0.5 0.0 -0.5
f1 (ppm)

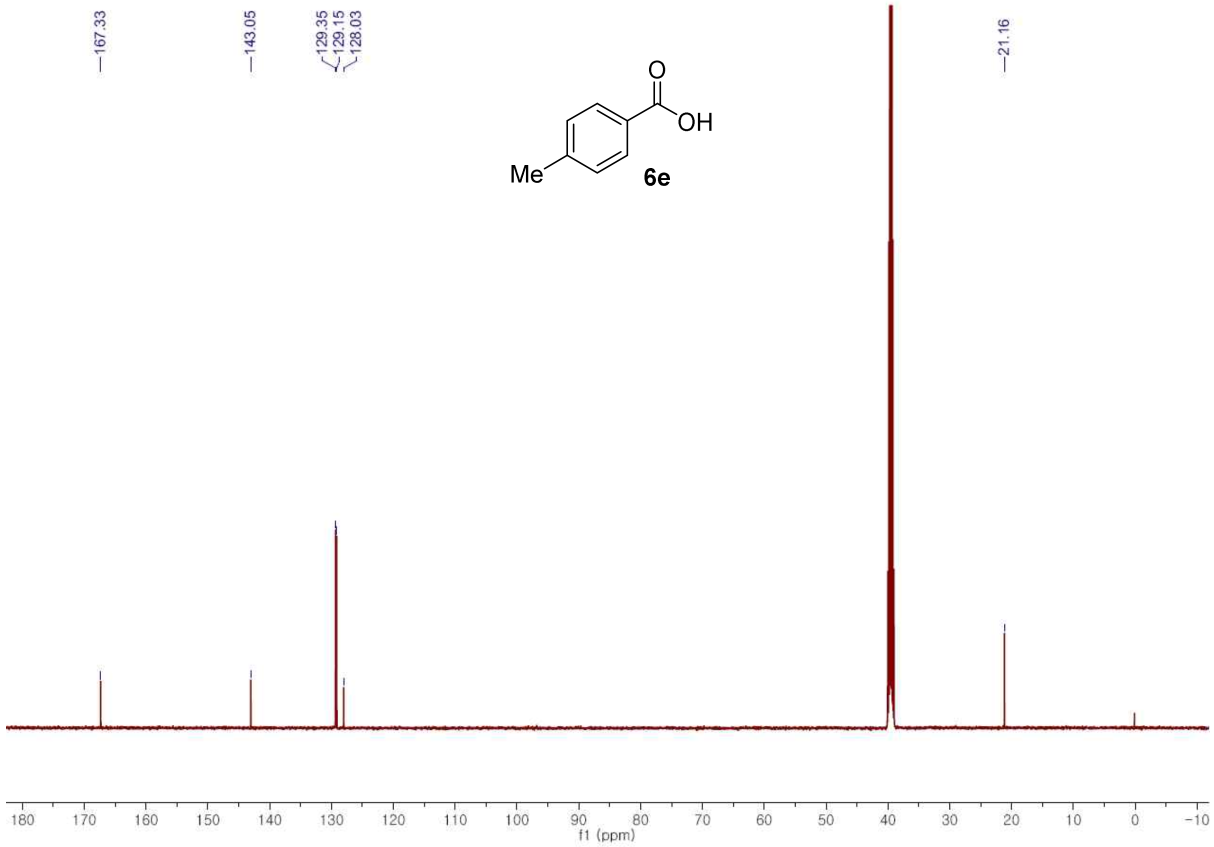
167.33
143.05
129.35
129.15
128.03
21.16
O
OH
Me
6e
180 170 160 150 140 130 120 110 100 90 80 70 60 50 40 30 20 10 0 -10
f1 (ppm)

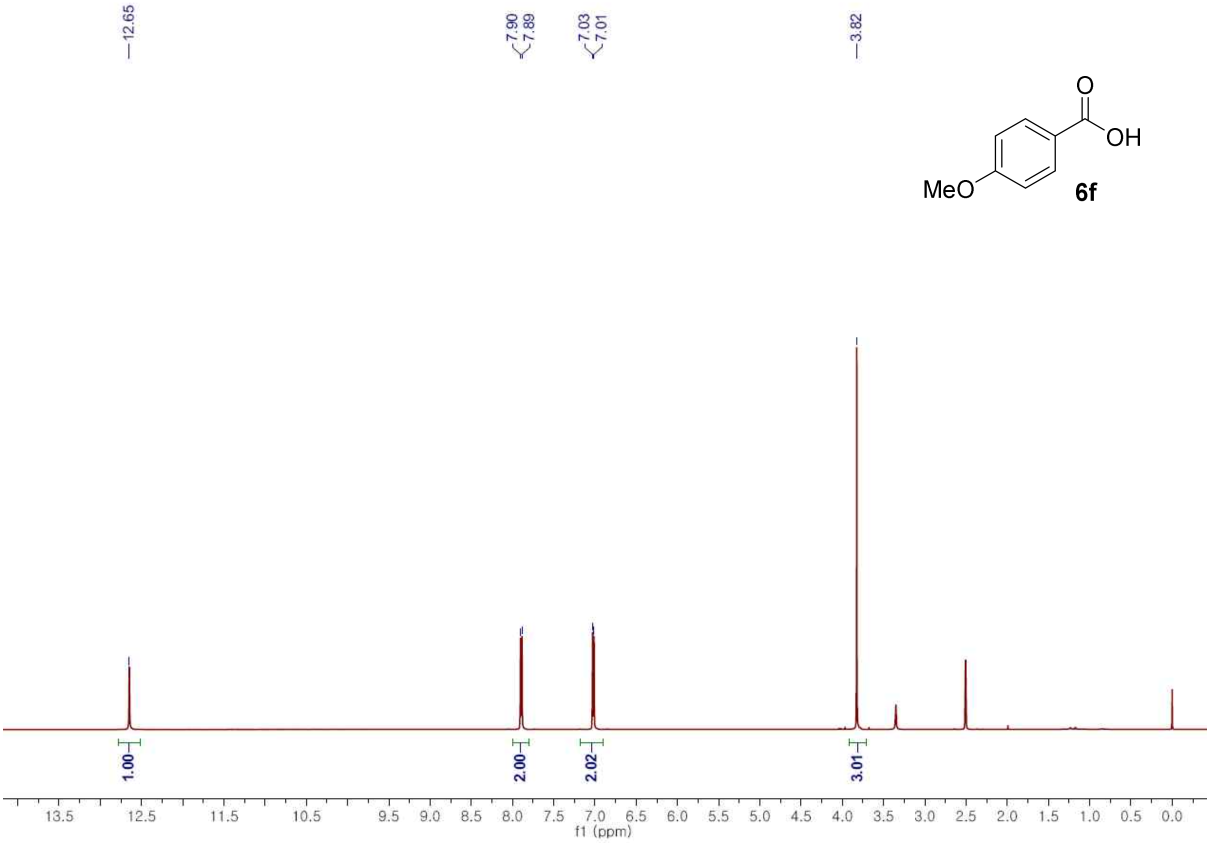
12.65
7.90
7.89
7.03
7.01
3.82
O
OH
MeO
6f
1.00
2.00
2.02
3.01
13.5 12.5 11.5 10.5 9.5 9.0 8.5 8.0 7.5 7.0 6.5 6.0 5.5 5.0 4.5 4.0 3.5 3.0 2.5 2.0 1.5 1.0 0.5 0.0
f1 (ppm)

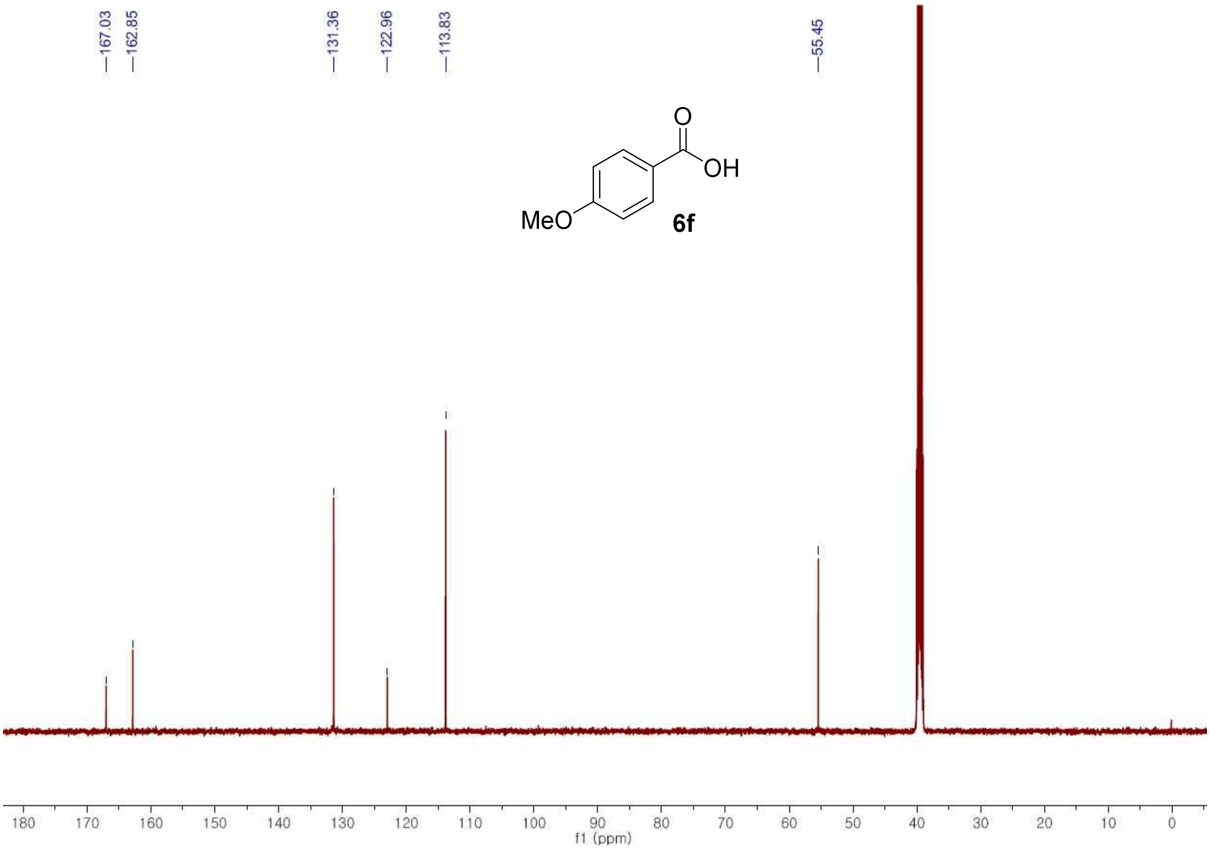
167.03
162.85
131.36
122.96
113.83
55.45
O
OH
MeO
6f
180 170 160 150 140 130 120 110 100 90 80 70 60 50 40 30 20 10 0
f1 (ppm)

Analysis of Graphene Oxide (GO) and Reduced Graphene Oxide (rGO)

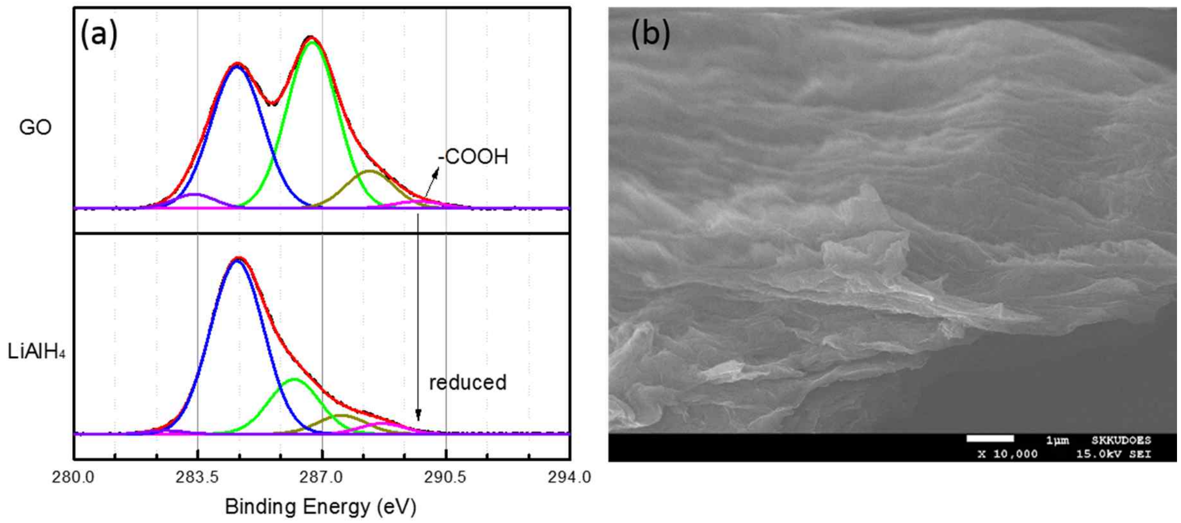

(a) XPS spectra of GO and rGO (b) SEM cross section image of GO

Test for NO_2 Absorption using GO

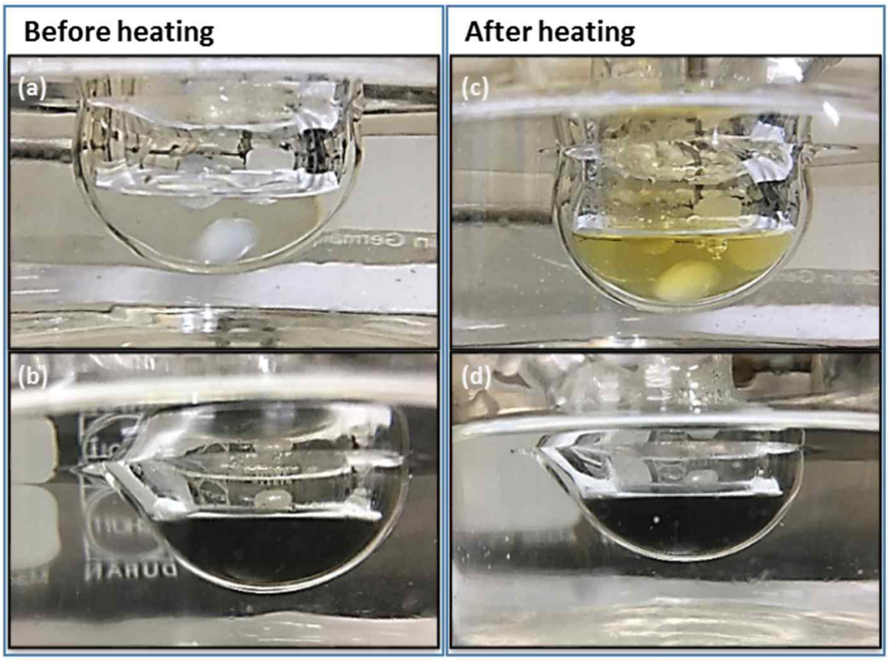

Before heating (a,b): The oxidation of benzyl alcohol with HNO_3 was carried out either in the absence (a) or presence of GO (b) at room temperature; **After heating** (c,d): The oxidation of benzyl alcohol with HNO_3 was carried out either in the absence (c) or presence of GO (d) at 90 ^{o}C. The brown gas NO_2 did not observe under reaction condition d, it indicated that the decomposed NO_2 gas can easily be adsorbed by GO because of vacancies or small holes on the GO surface.

Recyclability of the GO Carbocatalyst

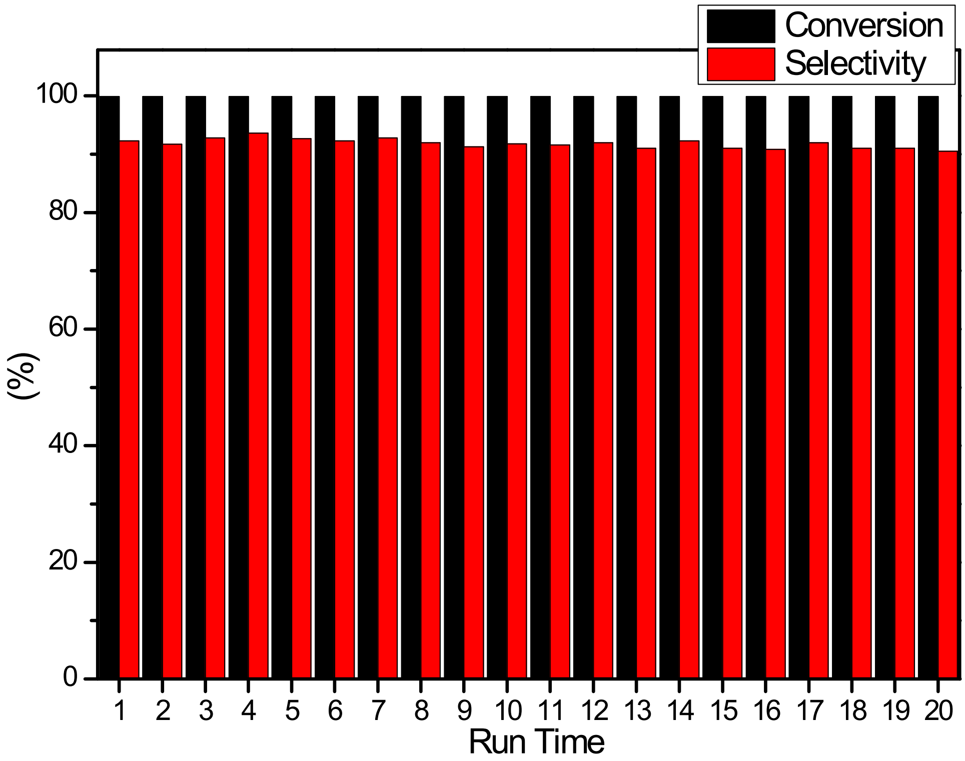

Supplement: Supplementary file 1 — Supplementary Information [file 41598_2017_3468_MOESM1_ESM.pdf]
